# Diet and biliary tract cancer risk in Shanghai, China

**DOI:** 10.1371/journal.pone.0173935

**Published:** 2017-03-13

**Authors:** Shakira M. Nelson, Yu-Tang Gao, Leticia M. Nogueira, Ming-Chang Shen, Bingsheng Wang, Asif Rashid, Ann W. Hsing, Jill Koshiol

**Affiliations:** 1 Cancer Prevention Fellowship Program, Division of Cancer Prevention, National Cancer Institute, Rockville, Maryland, United States of America; 2 Metabolic Epidemiology Branch, Division of Cancer Epidemiology and Genetics, National Cancer Institute, Rockville, Maryland, United States of America; 3 Department of Epidemiology, Shanghai Cancer Institute, Shanghai, China; 4 Texas Cancer Registry, Cancer Epidemiology and Surveillance Branch, Texas Department of State Health Services, Austin, Texas, United States of America; 5 Department of Pathology, Shanghai Cancer Center, Fudan University, Shanghai, China; 6 Department of General Surgery, Zhongshan Hospital, School of Medicine, Fudan University, Shanghai, China; 7 Department of Pathology, The University of Texas MD Anderson Cancer Center, Houston, Texass, United States of America; 8 Stanford Cancer Institute, Palo Alto, California, United States of America; 9 Stanford Prevention Research Center, Stanford School of Medicine, Palo Alto, California, United States of America; 10 Infectious and Immunoepidemiology Branch, Division of Cancer Epidemiology and Genetics, National Cancer Institute, Rockville, MD, United States of America; Digestive Disease Research Center, Scott & White Healthcare, UNITED STATES

## Abstract

Trends in biliary tract cancer incidence rates have increased in Shanghai, China. These trends have coincided with economic and developmental growth, as well as a shift in dietary patterns to a more Westernized diet. To examine the effect of dietary changes on incident disease, we evaluated associations between diet and biliary tract cancers amongst men and women from a population-based case-control study in Shanghai, China. Biliary tract cancer cases were recruited from 42 collaborating hospitals in urban Shanghai, and population-based controls were randomly selected from the Shanghai Household Registry. Food frequency questionnaire data were available for 225 gallbladder, 190 extrahepatic bile duct, and 68 ampulla of Vater cancer cases. A total of 39 food groups were created and examined for associations with biliary tract cancer. Interestingly, only four food groups demonstrated a suggested association with gallbladder, extrahepatic bile duct, or ampulla of Vater cancers. The allium food group, consisting of onions, garlic, and shallots showed an inverse association with gallbladder cancer (OR: 0.81, 95% CI: 0.68–0.97). Similar trends were seen in the food group containing seaweed and kelp (OR: 0.79, 95% CI: 0.67–0.96). In contrast, both preserved vegetables and salted meats food groups showed positive associations with gallbladder cancer (OR:1.27, 95% CI: 1.06–1.52; OR: 1.18, 95% CI: 1.02–1.37, respectively). Each of these four food groups showed similar trends for extrahepatic bile duct and ampulla of Vater cancers. The results of our analysis suggest intake of foods with greater anti-inflammatory properties may play a role in decreasing the risk of biliary tract cancers. Future studies should be done to better understand effects of cultural changes on diet, and to further examine the impact diet and inflammation have on biliary tract cancer incidence.

## Introduction

A relatively rare disease, biliary tract cancer is divided into three anatomic types: gallbladder, extrahepatic bile duct, and ampulla of Vater [1, 2]. Regional variations in biliary tract cancer incidence have been well documented, with higher rates in Central and South America, and parts of Asia, including India and Shanghai, China [3, 4, 5]. Biliary tract cancer incidence rates in Shanghai increased from 1977–2010, in both men and women [6]. Women, however, have seen greater increases in incidence and number.

Diet has likely affected general disease incidence, as China has seen major economic and developmental growth, accompanied with an emerging obesity epidemic [7, 8]. Studies have shown that following a westernized dietary pattern high in red and processed meats, fats, simple carbohydrates and sugars shows positive associations with inflammatory markers [9, 10, 11, 12]. Today, Shanghai has significantly increased animal based diets, where pork is consumed at higher rates than processed and grilled meats [13]. Conversely, foods such as fruits, vegetables, fish, nuts, and whole grains, and dietary patterns such as the Mediterranean diet, are inversely associated with inflammation and inflammatory markers like C-reactive protein (CRP) and IL-6 [9, 14, 15, 16, 17]. Moreover, a recent study in Nepal demonstrated high intake of fruits to be a protective factor for gallbladder cancer [18]. These pro- and anti-inflammatory foods may be associated with cancer risk.

Diet has been associated with many cancers in China. For example, consumption of salted fish, a staple in many Chinese homes, has been linked to increased inflammation and incidence of nasopharyngeal carcinoma [19], while consumption of red meat, fish, and eggs have been associated with increased incidence of colon cancer [20]. The link between diet and biliary tract cancer is less clear, however. Although epidemiological case-control studies, and a recent meta-analysis, have shown associations between diet, obesity, and biliary tract cancer [21, 22, 23, 24], the World Cancer Research Fund and the American Institute of Cancer Research (WCRF/AICR) have determined associations between diet, supplement intake and biliary tract cancer are “limited-no conclusion” [25], indicating that more research is needed. In this report, we evaluated the role of diet and its associations with biliary tract cancers amongst men and women from a population-based case-control study in Shanghai, China.

## Materials and methods

### Study population

In a population-based study conducted in Shanghai, China, incident biliary tract cancers were identified between June 1997 and May 2001. Cases, ages 35–74, were identified using a rapid reporting system established across 42 collaborating hospitals in urban Shanghai [3, 26]. Collectively, these hospitals captured over 95% of the eligible biliary tract cancer cases in Shanghai during the study period. We enrolled a total of 627 cases, including 368 gallbladder, 191 extrahepatic bile duct, and 68 ampulla of Vater cancer patients (ICD9 156). To assess and identify unique and shared risk factors associated with biliary tract cancers, 1,037 biliary stone cases without a history of cancer (774 with gallstones and 263 with bile duct stones) were selected from the same hospital as the cancer cases and frequency-matched on age (5-year groups) and sex. Moreover, a total of 959 permanent residents of Shanghai, with no history of cancer, were randomly selected from the Shanghai Household Registry as population controls and frequency-matched to cases based on age (5-year groups) and sex. Written informed consent was provided by all study subjects. The Institutional Review Boards of the National Cancer Institute and Shanghai Cancer Institute approved the study protocol.

### Clinical and pathology review

Surgical and pathology reports from local hospitals were used to diagnose all biliary tract cancer cases. Each was confirmed as previously described [3] by a panel of clinicians. Histological confirmation for each cancer was completed by expert review [3, 27]. Only 70% of the cancer cases had available materials to use for confirmation due to the late-stage diagnosis of most biliary tract cancers. For cases without pathology material, image data, medical records, and surgical reports were reviewed. Biliary stone cases were confirmed clinically, through review of abdominal ultrasound data or ERCP films [22].

### Interviews

In-person interviews were conducted for all study subjects as previously described [3]. Briefly, subjects were interviewed using a structured questionnaire, collecting information on demographics, diet, and life-style factors. Information on history of gallstones was also collected. Each interview was taped, reviewed, and verified to ensure study protocol adherence. The interview response rate for cases was > 95% and 82% for population controls. To check interview reproducibility, 5% of controls were randomly selected and re-interviewed 3 months after the original. Concordance was over 95%.

### Creation of food groups

We used a food frequency questionnaire with portion size to assess dietary intake. Portion size was capture by the liang (with one liang equivalent to 50 grams). The questionnaire included 115 dietary food items, posing questions such as: ‘Five years ago, how many times per day/week/month did you eat the following foods, and how many liang of each food did you eat?’. Each food items in the FFQ is treated as a continuous variable, representing amount consumed in a day. Using the median as the cut-off, each food item was divided into high and low intake. Foods identified in the questionnaire were categorized into 39 food groups using information gathered from previous studies and AICR, WCRF, and International Agency for Research on Cancer (IARC) reports including food likeness, association with inflammatory markers and cancer, and food type [19, 25, 28]. Vegetables, fish, red meat, poultry, beans, fats, and proteins were categorized into representative groups. For example, bacon, pork chops, spare ribs, pig feet, pork, and fried pork were categorized together (S1 Table).

### Statistical analysis

To determine associations between biliary tract cancers and diet, we performed unconditional logistic regression to calculate odds ratios (ORs) and 95% confidence intervals (CIs). Biliary tract cancer cases were compared to controls within each food group category (low intake = reference group). Population-based controls without a history of cholecystectomy (n = 884) were compared to gallbladder cases. Extrahepatic bile duct and ampulla of Vater cancer cases were compared with all population controls (n = 947). Gallstone controls (n = 774) were also compared with all population controls. Forward selection was used to create the final regression model, where age, sex, education, gallstones and body mass index (BMI; weight (kg)/height (m^2^)) accounted for at least a 10% change in the association between diet and each cancer. Additionally, hepatitis, coronary heart disease (CHD), liver cirrhosis, and liver cancer were evaluated. However, each variable showed no change in the ORs of any food group by 10% or more, and therefore were not included in the final model. Total energy intake was adjusted for using the residuals method [29]. Within each food group, high and low food intake were compared, with low intake as the referent group. Differences between cases and controls were detected using Fisher’s exact test. Stratification by sex was used to investigate effect modification of the associations between biliary tract cancers and diet by sex for gallbladder cancer and extrahepatic bile duct cancer. Numbers were too limited to evaluate ampulla of Vater cancer. The Wald test was used to statistically test for interaction on the multiplicative scale. To test for multiple comparisons, we applied a Bonferroni correction based on 39 food groups (adjusted p-value = 0.001).

All statistical tests were two-sided, and p-values < 0.05 were considered statistically significant. Analyses were performed using SAS software, version 9.3.

## Results

Most biliary tract cancer patients in this case-control study were married with a normal BMI (18.5–25 kg/m^2^), did not report diabetes, and were never smokers (Table 1). Population controls were more likely to be never drinkers, while gallbladder, extrahepatic bile duct, and ampulla of Vater cases were more likely to be drinkers. Moreover, gallbladder cancer cases were more likely to be women, while extrahepatic bile duct and ampulla of Vater cases were both more likely to be men.

**Table 1:** Select characteristics of study participants.

	Controls	Cases			
	PBC^a^	GBC	PBC^b^	EBC	AV
	(n = 884)	(n = 225)	(n = 947)	(n = 190)	(n = 68)
Age, mean ± SD	63.6 ± 8.5	63.9 ± 8.7	63.8 ± 8.4	63.6 ± 8.4	65.2 ± 7.1
Sex					
Male, N (%)	350 (39.6)	72 (32.0)	366 (38.6)	98 (51.6)	37 (54.4)
Female, N (%)	534 (60.4)	153 (68.0)	581 (61.3)	92 (48.4)	31 (45.6)
Education, N (%)					
None/Primary	355 (40.1)	119 (52.9)	393 (41.5)	85 (44.7)	29 (42.6)
Jr. Middle	220 (24.9)	53 (23.6)	231 (24.4)	43 (22.6)	16 (23.5)
Sr. Middle	180 (20.4)	30 (13.3)	186 (19.6)	31 (16.3)	15 (22.1)
≥ Community college	129 (14.6)	23 (10.2)	137 (14.5)	30 (15.8)	8 (11.7)
Marital status, N (%)					
Married	690 (78.1)	174 (77.3)	741 (78.2)	160 (84.2)	55 (80.9)
Divorced	17 (1.9)	4 (1.8)	18 (1.8)	1 (0.5)	1 (1.5)
Widowed	162 (18.3)	45 (20.0)	173 (18.3)	27 (14.2)	12 (17.6)
Single	12 (1.4)	2 (0.89)	12 (1.3)	2 (1.1)	0 (0.0)
Body mass index^#^, N (%)*					
Underweight	74 (8.4)	9 (4.0)	75 (7.9)	8 (4.2)	1 (1.5)
Normal	549 (62.1)	112 (49.8)	579 (61.1)	126 (66.3)	42 (61.8)
Overweight	206 (23.3)	73 (32.4)	229 (24.2)	39 (20.5)	22 (32.6)
Obese	31 (3.5)	11 (4.9)	37 (3.9)	3 (1.6)	0 (0.0)
Ever smoker, N (%)*					
No	610 (69.0)	161 (71.6)	654 (69.1)	114 (60.0)	37 (54.4)
Yes	274 (31.0)	63 (28.0)	293 (30.9)	76 (40.0)	31 (45.6)
Ever drinker, N (%)*					
No	704 (79.6)	34 (15.1)	755 (79.7)	49 (25.7)	15 (22.1)
Yes	180 (20.4)	191 (84.9)	192 (20.3)	141 (74.2)	53 (77.9)
Self-reported diabetes, N (%)*					
No	820 (92.7)	194 (86.2)	872 (92.1)	170 (89.5)	63 (92.6)
Yes	64 (7.2)	30 (13.3)	75 (7.9)	20 (10.5)	5 (7.4)

*Not all variable columns add up to 100% due to missing values.

^a^population controls (PBC) without a history of cholecystectomy compared to gallbladder cancer (GBC) cases.

^b^all-population controls (PBC) compared with extrahepatic bile duct (EBC) and Ampulla of Vater (AV) cancer case.

^#^self-reported BMI 5 years prior to interview.

For most of the 39 food groups created, we did not see consistent associations within the cancer sites, nor across the cancer sites (S1 Table). Four food groups, however, showed interesting patterns. The allium vegetable group, including onions, shallots, and garlic, showed an inverse association with gallbladder (OR: 0.81, 95% CI: 0.68–0.97), extrahepatic bile duct (OR: 0.77, 95% CI: 0.64–0.92), and ampulla of Vater cancers (OR: 0.74, 95% CI: 0.49–1.14), compared to the appropriate population-based controls (Table 2). Similar trends were seen in the food group that consisted of seaweed and kelp. Both of these food groups showed a 20% or greater reduction in biliary tract cancer risk. In contrast, preserved vegetables were positively associated across the three cancer sites; the strongest association was with extrahepatic bile duct cancer (OR: 1.37, 95% CI: 1.14–1.65). Salted meats and salted fish were also positively associated across all three cancer sites and showed the strongest association with ampulla of Vater cancer; ampulla of Vater cancer cases were 74% more likely to have consumed salted meats and fish than population-based controls (OR: 1.74, 95% CI: 1.16–2.61). However, none of the associations for the four food groups were significant at the Bonferroni-corrected level.

**Table 2:** Adjusted ORs (95% CIs) for associations between diet food groups (Fg) and biliary tract cancers^a^.

	Gallbladder Cancer			Extrahepatic Bile Duct			Ampulla of Vater		
	OR	95% CI	p-value	OR	95% CI	p-value	OR	95% CI	p-value
Fg1: Onions, shallots, and garlic	0.81	0.68–0.97	*0*.*02*	0.77	0.64–0.92	*0*.*01*	0.74	0.49–1.14	*0*.*17*
Fg2: Seaweed and kelp	0.79	0.67–0.96	*0*.*02*	0.68	0.56–0.84	*0*.*01*	0.65	0.39–1.09	*0*.*10*
Fg3: Preserved vegetables	1.27	1.06–1.52	*0*.*01*	1.37	1.14–1.65	*0*.*01*	1.34	0.89–2.02	*0*.*17*
Fg4: Salted meat, salted fish	1.18	1.02–1.37	*0*.*03*	1.19	1.01–1.39	*0*.*03*	1.74	1.16–2.61	*0*.*01*

^a^Models adjusted for: age, sex, education, gallstones, kcal and body mass index.

Comparing gallbladder cancer to gallstone controls, associations with the four food groups were in the same direction as the associations for gallbladder cancer compared to population controls. However, only the association with the seaweed and kelp food group was statistically significant (OR: 0.82, 95% CI: 0.70–0.96, p-value = 0.03). Upon examining associations with gallstones, the food group containing onions, shallots, and garlic was inversely associated with gallstones compared to population controls (OR: 0.78, 95% CI: 0.64–0.96, p-value = 0.02) (S2 Table). Both the preserved vegetables food group and the salted meats and salted fish food group showed positive associations with gallstones compared to population-based controls (OR: 1.18, 95% CI: 0.94–1.49; OR: 1.21, 95% CI: 0.99–1.46, respectively).

In our stratified models, associations between gallbladder cancer and each food group showed some differences between men and women (Table 3). In women, gallbladder cancer was inversely associated with the onions, shallots, and garlic food group (OR: 0.69, 95% CI: 0.54–0.88), and the preserved vegetables and salted meats/fish food groups had positive associations (OR: 1.48, 95% CI: 1.19–1.84; OR: 1.12, 95% CI: 1.01–1.45, respectively). Conversely, in men only the seaweed and kelp food group showed an inverse association with gallbladder cancer (OR: 0.65, 95% CI: 0.47–0.91), while it was null in women (OR: 0.88, 95% CI: 0.70–1.09). However, there was no statistically significant modification by sex for gallbladder cancer across all four food groups on the multiplicative scale (P-interaction range = 0.07–0.25). Furthermore, stratification revealed no modification by sex of the associations between extrahepatic bile duct cancer and any of the four food groups (P-interaction range: 0.46–0.52) (Table 3).

**Table 3:** Adjusted ORs (95% CIs) for associations between diet food groups (Fg), gallbladder cancer, and extrahepatic bile duct cancer, stratified by sex^a^.

	Gallbladder Cancer					Extrahepatic bile duct		
**Men**	# cases	OR	95% CI	p-value	# cases	OR	95% CI	p-value
Fg1	72	1.01	0.78–1.32	*0*.*94*	98	0.86	0.68–1.08	*0*.*19*
Fg2	72	0.65	0.47–0.91	*0*.*01*	98	0.8	0.62–1.03	*0*.*09*
Fg3	72	0.88	0.62–1.24	*0*.*47*	98	1.40	1.08–1.81	*0*.*01*
Fg4	72	1.14	0.86–1.49	*0*.*35*	98	1.27	1.01–1.61	*0*.*05*
**Women**	# cases	OR	95% CI	p-value	# cases	OR	95% CI	p-value
Fg1	153	0.69	0.54–0.88	*0*.*01*	92	0.63	0.46–0.86	*0*.*01*
Fg2	153	0.88	0.70–1.09	*0*.*25*	92	0.54	0.38–0.76	*0*.*01*
Fg3	153	1.48	1.19–1.84	*0*.*01*	92	1.35	1.04–1.76	*0*.*02*
Fg4	153	1.12	1.01–1.45	*0*.*04*	92	1.14	0.92–1.41	*0*.*24*

^a^Models adjusted for: age, education, gallstones, kcal and body mass index.

## Discussion

In this population-based study, we evaluated 39 food groups and their association with gallbladder, extrahepatic bile duct, and ampulla of Vater cancer. In general, the associations between most dietary factors, food items and food groups, and biliary tract cancer were null. However, four food groups produced interesting results. Risk at all three biliary tract cancer sites was reduced with higher consumption of onions, shallots, and garlic and of seaweed and kelp. Conversely, consumption of preserved vegetables and salted and preserved meats and fish were associated with increased the risk of biliary tract cancer.

Animal studies have shown dietary garlic and onions reduce diet-induced cholesterol gallstones [30], a well-established risk factor for gallbladder cancer [3, 31, 32, 33]. Evidence from our study supports this finding, as consumption of both garlic and onions was associated with a moderately decreased risk of gallbladder cancer. In addition, seaweed, kelp, garlic, and onions may have anti-inflammatory properties [34, 35, 36]. To date, many of the findings from the Shanghai Study have been related to inflammation [27, 37]. Recent publications have shown increasing levels of CRP are associated with increased risk of gallbladder cancer [38] and other bile duct cancers [39]. Given these trends, if foods such as seaweed, kelp, garlic, and onions can reduce CRP and other inflammatory marker levels, as shown in previous studies [40, 41], they may also help reduce the risk of biliary tract cancers. Furthermore, results from a study from the EPIC cohort show increasing amounts of fiber decrease the risk of biliary tract cancer [42]. High amounts of fiber are contained in many foods, including seaweed and kelp, which were associated with decreased biliary tract cancer risk in our study.

In contrast, foods such as preserved vegetables and salted and preserved meats have been shown to have pro-inflammatory properties [19, 28]. The process of preserving vegetables consists of pickling with salt and brine. Evidence from epidemiological studies shows a strong association between esophageal cancer and consumption of preserved and pickled vegetables [43]. A recent meta-analysis showed a 52% increase in gastric cancer risk from consumption of pickled vegetables [44]; an 86% increase in gastric cancer risk was seen in those from mainland China [30]. In a study of Japanese men and women, high intake of salted foods including salted fish, increased the risk of gastric cancer in men (RR _Q5vs.Q1_: 2.23, p-value = 0.001); however, similar trends were not seen in women (RR_Q5vs.Q1_:1.32, p-value = 0.48) [45]. Very few studies have examined consumption of preserved vegetables and salted food in relation to biliary tract cancer risk, but in one study, the relative risk of gallstones decreased by 81% among those who consumed salted fish (RR: 0.19, 95% CI: 0.05–0.68) [46]. These findings are inconsistent with our study, since a decreased risk of gallstones would presumably be associated with a decreased risk of gallbladder cancer, while we found that increased salted fish was associated with an increased risk of gallbladder cancer. These differences may be due to environmental or genetic factors involved in the development of gallstones, and progression to gallbladder cancer. Differences in the kind and quantity of salted foods could also be related. For example, higher quantities of salted fish are consumed in the south of China than in Shanghai [19], while allium vegetables are more frequently consumed in Shanghai than other populations [47]. The positive association between preserved vegetables and salted meats is also supported by studies implicating nitrates and nitrites in carcinogenesis [48], and a cross-sectional study in India showed that nitrate concentrations in bile were significantly higher in gallbladder cancer cases compared to gallstone patients (mean nitrate concentration in gallbladder cancer cases = 79.33 mg/L vs. choleslithiasis cases = 3.37 mg/L) [49]. This potential biological plausibility provides compelling support for future investigations of these food components and biliary tract cancer risk to improve our understanding of the biologic mechanisms involved and potential cancer prevention strategies.

Although we did not see statistically significant effect modification by sex on a multiplicative scale with respect to gallbladder and extrahepatic bile duct cancer, there was some evidence of differences in associations with specific food groups for gallbladder cancer. Given the small number of cases in our study, additional studies are necessary to determine whether sex may modify the effect of dietary components on biliary tract cancer risk.

Our study has a number of strengths. It is a population-based study with high participation rates (> 80%). Although not terribly large, the study has sufficient numbers for us to examine associations between diet and the three biliary anatomic subsites separately, allowing us to evaluate patterns within and across these subsites. Moreover, we established food groups with known biological relevance supported by evidence from AICR and the WCRF. This study was also able to evaluate foods that are more commonly consumed in Shanghai than in other parts of the world. For example, seaweed, kelp, and salted fish are staples in Chinese kitchens, with less consumption in Western populations. This specificity has limited other studies from examining these foods in the context of biliary tract cancer risk, highlighting the novelty of our findings.

Limitations of the study include the case-control design, which may have influenced the observed associations through the introduction of recall bias (or reverse causation). Respondents were asked to report food consumption five years prior to the study in order to assess exposure prior to development of cancer. However, we cannot rule out potential associations due to chance. The small sample size also limited our ability to examine possible effect modification by sex, especially for ampulla of Vater cancer cases.

In conclusion, while most food groups were not associated with biliary tract cancers, we found modest to moderate associations between vegetables with anti-oxidant properties and decreased risk of biliary tract cancer. Conversely, consumption of meats and vegetables that have been salted or preserved, were associated with increased risk of biliary tract cancer. These results suggest that future studies of diet and inflammation may be warranted to better understand the impact of diet on biliary tract cancers.

## Supporting information

S1 Table A complete list of the 39 food groups, depicting associations between diet food groups three biliary tract cancer types. (PDF)

S2 Table Adjusted ORs (95% CIs) for associations between diet food groups (Fg) and gallstones. Models adjusted for: age, gender, education, kcal and body mass index.
(PDF)

S1 File Shanghai Cancer Institute Health Study questionnaire. (PDF)

S2 File The Nutrition and Health Study in Shanghai Enrollment interview guide. (PDF)
