# Supplementary material for: Diet and biliary tract cancer risk in Shanghai, China
Source: PLoS One. 2017 Mar 13;12(3):e0173935. doi: 10.1371/journal.pone.0173935 (PMC5348031; doi:10.1371/journal.pone.0173935)
Supplement: S1 Table — (PDF) [file pone.0173935.s001.pdf]

Supplemental Table 1: A complete list of the 39 food groups, depicting associations between diet food groups three biliary tract cancer types.

Food Groups	Gallbladder Cancer			Bile Duct Cancer			Ampulla of Vater		
	OR	95% CI	p-value	OR	95% CI	p-value	OR	95% CI	p-value
Fg1: onions, shallots, and garlic	0.81	0.68-0.97	0.02	0.77	0.64-0.92	0.004	0.74	0.49-1.14	0.17
Fg2: seaweed and kelp	0.79	0.67-0.96	0.02	0.68	0.56-0.84	0.003	0.65	0.39-1.09	0.10
Fg3: preserved vegetables	1.27	1.06-1.52	0.01	1.37	1.14-1.65	0.001	1.34	0.89-2.02	0.17
Fg4: salted meats and salted fish	1.18	1.02-1.37	0.03	1.19	1.01-1.39	0.03	1.74	1.16-2.61	0.01
Fg5: bacon, pork chops, spare ribs, pig feet, fat pork, pork, lean pork, beef, lard, and fried pork	1.13	0.91-1.41	0.25	0.88	0.69-1.10	0.26	1.15	0.85-2.62	0.16
Fg6: Chicken, fried chicken, duck	0.88	0.74-1.05	0.16	0.88	0.73-1.05	0.15	1.03	0.68-1.56	0.91
Fg7: saltwater fish	0.89	0.78-1.02	0.11	0.93	0.81-1.06	0.28	1.09	0.78-1.52	0.59
Fg8: Freshwater Fish, salted fish, fried fish	1.04	0.88-1.21	0.66	1.15	0.98-1.35	0.09	1.23	0.84-1.81	0.28
Fg9: eel	0.77	0.62-0.95	0.01	0.93	0.76-1.13	0.45	0.89	0.57-1.42	0.65
Fg10: shrimp, shellfish	0.87	0.75-1.02	0.08	1.01	0.87-1.19	0.82	1.01	0.69-1.46	0.98
Fg11: carrot, pumpkin, sweet potatoes	0.89	0.80-0.99	0.04	0.82	0.73-0.93	0.002	0.80	0.59-1.09	0.16
Fg12: spinach, kale, mustard greens	0.84	0.73-0.95	0.007	0.99	0.87-1.15	0.98	1.42	0.99-2.02	0.05
Fg13: Tomatoes, green peppers, eggplant, winter melon, bottle gourd, cucumber, luffa	0.92	0.74-1.13	0.41	1.24	0.98-1.56	0.07	1.69	0.95-3.03	0.08
Fg14: Pale sweet potato, radishes, turnip, lotus root, taro root, potato	0.83	0.72-0.97	0.02	0.91	0.77-1.07	0.24	0.95	0.66-1.35	0.76
Fg15: scallions, chives, garlic stalk	0.85	0.72-1.01	0.07	0.98	0.82-1.18	0.86	0.79	0.51-1.23	0.29
Fg16: celery cabbage, common cabbage, swamp cabbage, Chinese flat cabbage, chick feather cabbage, cauliflower	0.98	0.76-1.26	0.93	0.99	0.77-1.28	0.96	0.72	0.46-1.14	0.16
Fg17: snow peas, common peas, cowpea	0.91	0.79-1.05	0.21	1.18	1.01-1.37	0.04	1.53	1.04-2.24	0.03
Fg18: shiitake, button, woodear	0.89	0.77-1.05	0.17	1.11	0.94-1.32	0.22	1.41	0.92-2.14	0.11
Fg19: ginger	0.64	0.52-0.79	<0.001	0.83	0.66-1.04	0.11	0.59	0.34-1.04	0.07
Fg20: total vegetable	0.91	0.69-1.19	0.48	1.03	0.78-1.38	0.82	0.98	0.51-1.92	0.97
Fg21: snow pea leaves, celtuce, lettuce	0.98	0.86-1.12	0.80	0.98	0.85-1.12	0.75	1.27	0.92-1.76	0.15
Fg22: hot pepper	0.89	0.73-1.11	0.32	0.94	0.76-1.16	0.55	0.73	0.40-1.31	0.29

Fg23: apples, pears, oranges, bananas, grapes, peaches, watermelon, muskmelon	0.99	0.86-1.16	0.98	0.99	0.85-1.16	0.93	0.98	0.73-1.33	0.92
Fg24: fava beans, red beans, mung beans, green beans, hyacinth	0.89	0.76-1.07	0.22	1.08	0.89-1.31	0.43	1.23	0.78-1.96	0.38
Fg25: mung bean sprout, soybean sprouts, bamboo shoots	0.86	0.74-0.99	0.05	0.89	0.76-1.03	0.12	1.09	0.75-1.58	0.66
Fg26: rice, noodles, buns, dumplings, fried dough, spring rolls, bread sticks	1.56	0.91-2.66	0.11	0.75	0.45-1.26	0.27	0.72	0.22-2.39	0.59
Fg27: Fried tofu, tofu, other soybean products, soybeans, soy milk, soybeans	1.05	0.86-1.27	0.64	1.07	0.88-1.32	0.49	0.97	0.61-1.54	0.90
Fg28: boiled egg	0.99	0.88-1.13	0.93	0.88	0.77-1.01	0.07	1.07	0.88-1.32	0.49
Fg29: preserved eggs	0.86	0.68-1.05	0.12	0.95	0.77-1.17	0.63	1.29	0.96-1.73	0.09
Fg30: fermented Tofu	1.31	1.08-1.59	0.01	1.22	0.98-1.51	0.06	1.33	0.84-2.09	0.23
Fg31: salted duck egg	1.05	0.93-1.19	0.40	1.08	0.95-1.23	0.25	1.26	1.01-1.53	0.04
Fg32: peanut butter, peanuts	0.82	0.69-0.96	0.02	0.97	0.83-1.14	0.71	1.20	0.86-1.69	0.29
Fg33: dessert	0.97	0.87-1.08	0.59	0.95	0.85-1.06	0.34	1.08	0.83-1.41	0.55
Fg34: milk, ice cream	0.98	0.92-1.05	0.62	1.01	0.94-1.09	0.79	0.97	0.80-1.16	0.71
Fg35: butter and margarine	0.89	0.74-1.08	0.43	1.0	0.88-1.14	0.99	1.10	0.82-1.48	0.52
Fg36: chicken (non-fried)	0.85	0.70-1.02	0.08	0.83	0.68-1.00	0.05	1.07	0.68-1.66	0.78
Fg37: fried chicken	0.95	0.74-1.21	0.67	0.99	0.78-1.27	0.98	0.81	0.44-1.47	0.48
Fg38: preserved and pickled foods: eggs, vegetables, hot pickled mustard	1.10	0.92-1.32	0.30	1.23	1.02-1.48	0.03	1.51	0.97-2.33	0.06
Fg39: fried foods: chicken, egg, tofu, dumplings, fish, pork, dough	1.13	0.96-1.33	0.14	0.97	0.82-1.15	0.74	1.82	1.17-2.82	0.007
