# Supplementary material for: Diet and biliary tract cancer risk in Shanghai, China
Source: PLoS One. 2017 Mar 13;12(3):e0173935. doi: 10.1371/journal.pone.0173935 (PMC5348031; doi:10.1371/journal.pone.0173935)
Supplement: S2 Table — Models adjusted for: age, gender, education, kcal and body mass index. (PDF) [file pone.0173935.s002.pdf]

Supplemental Table 2: Adjusted ORs (95% CIs) for associations between diet food groups (Fg) and gallstones[a]

	OR	95% CI	p-value
Fg1: Onions, shallots, and garlic	0.78	0.64-0.96	0.02
Fg2: Seaweed and kelp	0.96	0.78-1.19	0.72
Fg3: Preserved vegetables	1.18	0.94-1.49	0.15
Fg4: Salted meat, salted fish	1.21	0.99-1.46	0.06

[a]Models adjusted for: age, gender, education, kcal and body mass index
