# Supplementary material for: Diet and biliary tract cancer risk in Shanghai, China
Source: PLoS One. 2017 Mar 13;12(3):e0173935. doi: 10.1371/journal.pone.0173935 (PMC5348031; doi:10.1371/journal.pone.0173935)
Supplement: S1 File — (PDF) [file pone.0173935.s003.pdf]

SECTION A. DEMOGRAPHIC INFORMATION

INTRODUCTION:

Hello, my name is _______________ from the Shanghai Cancer Institute. May I speak to (__________)? (IF ____________________ IS TEMPORARILY UNAVAILABLE TRY TO FIND OUT WHEN HE/SHE WILL BE AVAILABLE. IF ____________________ IS PERMANENTLY UNAVAILABLE, TRY TO FIND OUT WHO IS THE NEXT-OF-KIN AND HOW TO REACH HIM/HER. IF ____________________ IS AVAILABLE, CONTINUE.)

The Shanghai Cancer Institute is doing a health study. (You have/________ has) been selected (at random) to take part in this study. I will be asking questions about (your/______'s) diet, medical history, physical activity, smoking habits, and other topics. The answers you provide will be kept confidential and used only for the purposes of this study.

Let's begin with some questions about (your/__________'s) background.

A1. (ASK ONLY IF NECESSARY.) Was ______ male or female?

MALE 1
FEMALE .... 2 |__|

A2. What (is your/was _____'s) date of birth?

|__|__| - |__|__| - |__|__|
YR MO DA
DON'T KNOW 989898

A3. Is this date based on the lunar or Western calendar?

LUNAR 1
WESTERN .... 2 |__|
DON'T KNOW 8

A4. What was (your/_____'s) age on (your/______'s) last birthday?

|__|__|
AGE
DON'T KNOW .... 98 |__|__|

A5. In what province, city, and county (were you/was _____) born?

PROVINCE: ________________________ |__|__|
CITY: ______________________________ |__|__|__|
COUNTY ___________________________ |__|__|__|__|

A6. How many years of schooling (have you/had _____) completed? (CIRCLE HIGHEST YEAR COMPLETED)

NONE	0	
PRIMARY SCHOOL	1	
JUNIOR MIDDLE SCHOOL	2	
SENIOR MIDDLE SCHOOL (POLYTECHNIC SCHOOL)	3	
COLLEGE (TELE-, NIGHT-, TRAINING COLLEGE)	4	
ABOVE COLLEGE	5	
OTHER (SPECIFY) ________________________________	6	
DON'T KNOW	8	|__|

A7. (What is your current marital status?/What was _____'s marital status at the time of death?) (Are you currently/Was _____): (READ)

Married,	1	
Living together but not married,	2	
Widowed,	3	
Divorced,	4	
Separated, or	5	
Never married?	6	
DON'T KNOW	8	|__|

SECTION B. SMOKING HISTORY

Now I have some questions about smoking.

B1. (Have you/Had _____) ever smoked a total of 100 cigarettes or more in (your/his/her) lifetime?

YES 1
NO .... 2 } (SECT. C)
DON'T KNOW .... 8 } (SECT. C) |__|

B2. Did (you/_____) ever smoke cigarettes regularly, that is, at least one per day for six months or longer?

YES 1
NO .... 2 } (SECT. C)
DON'T KNOW .... 8 } (SECT. C) |__|

B3. How old (were you/was _____) when (you/_____) first started smoking at least one cigarette per day?

|__|__|
AGE STARTED
DON'T KNOW .... 98 |__|__|

B4. (Do you currently/Did _____) smoke at least one cigarette per day (at the time of his/her death)?

YES.. 1 (B6)
NO 2
DON'T KNOW .... 8 (B6) |__|

B5. How old (were you/was _____) when (you/_____) last stopped smoking cigarettes regularly?

|__|__|
AGE STOPPED
DON'T KNOW .... 98 |__|__|

B6. For how many years in total, not counting the years (you/_____) stopped, did (you/_____) smoke cigarettes regularly?

|__|__|
YEARS
DON'T KNOW .... 98 |__|__|

B7. Thinking about all of the years when (you/_____) smoked, how many cigarettes did (you/_____) usually smoke in a day?

|__|__|
CIGARETTES/DAY
DON'T KNOW .... 98 |__|__|

SECTION C. BEVERAGE CONSUMPTION

Now I will ask questions about beverages (you/_____) consumed.

C1. Did (you/_____) ever drink any alcoholic beverages, such as beer, wine, or hard liquor, on a regular basis, that is, once a week or more for six months or longer?

YES 1
NO .... 2 } (C8)
DON'T KNOW .... 8 } (C8) |__|

C2. At what age did (you/_____) start drinking alcoholic beverages regularly?

|__|__|
AGE STARTED
DON'T KNOW .... 98 |__|__|

C3. (Do you still drink alcoholic beverages regularly?/Did ______ drink alcoholic beverages regularly until the time of his/her death?)

YES... 1 (C5)
NO 2
DON'T KNOW .... 8 (C5) |__|

C4. At what age did (you/_____) stop drinking alcoholic beverages regularly?

|__|__|
AGE STOPPED
DON'T KNOW .... 98 |__|__|

C5. For how many years in total did (you/_____) drink alcoholic beverages regularly, excluding the periods when (you/he/she) didn't drink?

|__|__|
YEARS
DON'T KNOW .... 98 |__|__|

C6. On the average, during (your/_____'s) adult life, how many (bottles/liang) of (BEVERAGE) did (you/he/she) drink per week?			C7. For how many years did (you/_____) drink (BEVERAGE)?
1. Beer	|__|__|__| #	Bottles .... 1<br>Liang .... 2<br>NONE .... 0<br>DON'T KNOW ... 8 } (C6.2) |__|	|__|__| # YEARS<br>DON'T KNOW 98
2. Yellow wine	|__|__|__| #	Bottles .... 1<br>Liang .... 2<br>NONE .... 0<br>DON'T KNOW ... 8 } (C6.3) |__|	|__|__| # YEARS<br>DON'T KNOW 98
3. Sweet wine	|__|__|__| #	Bottles .... 1<br>Liang .... 2<br>NONE .... 0<br>DON'T KNOW ... 8 } (C6.4) |__|	|__|__| # YEARS<br>DON'T KNOW 98
4. Hard liquor	|__|__|__| #	Bottles .... 1<br>Liang .... 2<br>NONE .... 0<br>DON'T KNOW ... 8 } (C8) |__|	|__|__| # YEARS<br>DON'T KNOW 98

C8. As an adult, did (you/_____) ever drink at least one cup of tea per day for 6 months or longer?

YES 1
NO .... 2 } (C15)
DON'T KNOW .... 8 } (C15) |__|

C9. How old (were you/was _____) when (you/_____) started drinking tea regularly?

|__|__|
AGE STARTED
DON'T KNOW .... 98 |__|__|

C10. (Do you still drink tea regularly?/Did _____ drink tea regularly until the time of his/her death?)

YES... 1
NO 2
DON'T KNOW .... 8 |__|

C11. For how many years in total did (you/_____) drink tea regularly, excluding the periods when (you/_____) didn't drink?

|__|__|
YEARS
DON'T KNOW .... 98 |__|__|

C12. As an adult, what kind of tea did (you/_____) usually drink?

GREEN TEA	1	
BLACK TEA	2	
BOTH OF THE ABOVE	3	
OTHER (SPECIFY) __________________		
____________________________________	6	
DON'T KNOW	8	|__|

C13. (Do you/Did _____) usually drink tea: (READ)

Strong,...	1	
Medium, or	2	
Weak?	3	
DON'T KNOW	8	|__|

C14. On the average, how many liang of tea (do you/did _____) drink per week, per month or per year?

|__|__|	PER WEEK	1	
# LIANG	PER MONTH	2	
	PER YEAR	3	
	DON'T KNOW	988	|__|__|__|

C15. As an adult, did (you/______) ever regularly drink ginseng tea or use ginseng for at least 6 months or longer?

YES	1		
NO	2	} (SEC. D)	|__|
DON'T KNOW	8		

C16. Did (you/_____) usually use . . .

Ginseng tea, ...	1	(SEC. D)	
Ginseng, or..	2		
Both?...	3		
DON'T KNOW	8		|__|

C17. What kind of ginseng did (you/______) usually have? Did (you/_____) have . . .

White ginseng, ...	1	
Red ginseng, ...	2	
Foreign ginseng, or	3	
Another type of ginseng?............................	4	
(SPECIFY)___________________________		
DON'T KNOW	8	|__|

C18. When (you/______) had ginseng, how many liang per week, month or year of ginseng did (you/_____) usually have?

|__|__|	PER WEEK	1
# LIANG	PER MONTH	2
	PER YEAR	3
	DON'T KNOW	988

|__|__|__|

SECTION D. MEDICAL HISTORY

Now I'm going to ask some questions about (your/________'s) medical history and medications (you've/________ has) taken.

ASK D2-D3, AS INDICATED FOR EACH CONDITION REPORTED IN D1.

D1. Did a doctor ever tell (you/________) that (you/_____) had (CONDITION)?	YES	NO	DK		D2. How old were (you/ ____) when the doctor first told (you/____) that (you/_____) had (CONDITION)? AGE	DK	D3. (Have you/Had__________) ever been hospitalized for this condition? YES	NO	DK	
a. High blood pressure	1	2	8	|__|	|__|__|	98	1	2	8	|__|
b. High cholesterol or other blood fat	1	2	8	|__|	|__|__|	98	1	2	8	|__|
c. Coronary heart disease	1	2	8	|__|	|__|__|	98	1	2	8	|__|
d. Hirsutism (excessive body hair)	1	2	8	|__|	|__|__|	98	1	2	8	|__|
e. Kidney or bladder infections	1	2	8	|__|	|__|__|	98	1	2	8	|__|
f. Tuberculosis	1	2	8	|__|	|__|__|	98	1	2	8	|__|
g. Chronic gastritis	1	2	8	|__|	|__|__|	98	1	2	8	|__|
h. Gastric ulcer	1	2	8	|__|	|__|__|	98	1	2	8	|__|
i. Duodenal ulcer	1	2	8	|__|	|__|__|	98	1	2	8	|__|
j. Acute pancreatitis	1	2	8	|__|	|__|__|	98	1	2	8	|__|
k. Chronic pancreatitis	1	2	8	|__|	|__|__|	98	1	2	8	|__|
l. Appendicitis	1	2	8	|__|	|__|__|	98	1	2	8	|__|
m. Crohn's disease	1	2	8	|__|	|__|__|	98	1	2	8	|__|
n. Ulcerative colitis	1	2	8	|__|	|__|__|	98	1	2	8	|__|
o. Other inflammatory bowel disease	1	2	8	|__|	|__|__|	98	1	2	8	|__|
p. Colon polyps	1	2	8	|__|	|__|__|	98	1	2	8	|__|
q. Familial polyposis	1	2	8	|__|	|__|__|	98	1	2	8	|__|
r. Any other digestive tract diseases (SPECIFY)___________	1	2	8	|__|	|__|__|	98	1	2	8	|__|
s. Obesity	1	2	8	|__|	|__|__|	98	1	2	8	|__|
t. Cystic fibrosis	1	2	8	|__|	|__|__|	98	1	2	8	|__|

ASK D5-D8, AS INDICATED FOR EACH CONDITION REPORTED IN D4.

D4. Did a doctor ever tell (you/________) that (you/_____) had (CONDITION)? YES NO DK	D5. How old were (you/ ____) when the doctor first told (you/____) that (you/_____) had (CONDITION)? AGE DK	D6. (Have you/Had____) ever been hospitalized for this condition? YES NO DK	D7. (Have you/Had____) ever been prescribed medicine including herbal medications for this condition? YES NO DK	D8. (ASK IF ANY MEDICATION IS INDICATED IN D7) What medications were prescribed for this condition?
a. Jaundice .... 1 2 8 |__|	|__|__| 98	1 2 8 |__|	1 2 8 |__|	______________ ______________
b. Schistosomiasis or liver flukes .... 1 2 8 |__|	|__|__| 98	1 2 8 |__|	1 2 8 |__|	______________ ______________
c. Acute hepatitis .... 1 2 8 |__|	|__|__| 98	1 2 8 |__|	1 2 8 |__|	______________ ______________
d. Chronic hepatitis......... 1 2 8 |__|	|__|__| 98	1 2 8 |__|	1 2 8 |__|	______________ ______________
e. Cirrhosis of liver......... 1 2 8 |__|	|__|__| 98	1 2 8 |__|	1 2 8 |__|	______________ ______________
f. Other disease of liver (SPECIFY)___________ 1 2 8 |__|	|__|__| 98	1 2 8 |__|	1 2 8 |__|	______________ ______________
g. Biliary cirrhosis .... 1 2 8 |__|	|__|__| 98	1 2 8 |__|	1 2 8 |__|	______________ ______________

BOX D-1

IF YES TO D4d, ASK D9.
OTHERWISE, CHECK HERE ☐ AND SKIP TO D10.

D9. You indicated that a doctor told (you/him/her) that (you/_____) had chronic hepatitis. (Are you/was _____) a chronic carrier of hepatitis?

YES	1	
NO	2	
DON'T KNOW	8	|__|

D9a. What type of hepatitis was it? (CIRCLE ALL THAT APPLY)

HEPATITIS A	1	|__|
HEPATITIS B	2	|__|
HEPATITIS C	3	|__|
DON'T KNOW	8	|__|

D10. Did a doctor ever tell (you/___) that (you/_____) had gallstones?

YES	1		
NO	2	} (D28)	|__|
DON'T KNOW	8	} (D28)	

D11. How old (were you/was ___) when the doctor first told (you/___) that (you/_____) had gallstones?

|__|__|
AGE

DON'T KNOW	98	|__|__|

D12. How were the gallstones diagnosed? Were they diagnosed using (METHOD)...

	YES	NO	DK	
a. Ultrasound?	1	2	8	|__|
b. X-ray?	1	2	8	|__|
c. CT scan?	1	2	8	|__|
d. MRI?	1	2	8	|__|
e. Dye or oral cholecystogram?	1	2	8	|__|
f. Injected radio-isotopes?	1	2	8	|__|
g. Physical examination only?	1	2	8	|__|
h. Any other method? (SPECIFY) ____________________________	1	2	8	|__|

D13. Did (you/_______) ever have any of the following symptoms associated with the gallstones? Did (you/_____) have (SYMPTOM)...

	YES	NO	DK	
a. Fever?	1	2	8	|__|
b. Jaundice?	1	2	8	|__|
c. Vomiting?	1	2	8	|__|
d. Nausea?	1	2	8	|__|
e. Abdominal pain?	1	2	8	|__|
f. Abdominal pain after eating?	1	2	8	|__|
g. Loss of appetite?	1	2	8	|__|
h. Dark urine?	1	2	8	|__|
i. Light-colored stools?	1	2	8	|__|

D14. (Have you/Had ____) ever been hospitalized for gallstones?

YES 1
NO .... 2 } (D18)
DON'T KNOW .... 8 } (D18) |__|

D15. How many times (were you/was ____) hospitalized for gallstones?

|__|__|
OF TIMES HOSPITALIZED
DON'T KNOW .... 98 |__|__|

D16. What was the date when (you were/_____ was) first hospitalized for gallstones?

|__|__| - |__|__| - |__|__|
YR MO DA
DON'T KNOW 989898

D17. What is the name and province of the hospital where (you were/____ was) first hospitalized for this condition?

NAME: ______________________________

PROVINCE: ______________________________

D18. Did a doctor ever tell (you/____) that (you/_____) had an obstruction of the bile duct caused by gallstones?

YES 1
NO .... 2 } (D21)
DON'T KNOW .... 8 } (D21) |__|

D19. At what age (were you/was _____) first told that (you/_____) had an obstruction of the bile duct caused by gallstones?

|__|__|
AGE
DON'T KNOW .... 98 |__|__|

D20. How many episodes of bile duct obstruction (have you/had ___) experienced?

|__|__|
OF EPISODES
DON'T KNOW .... 98 |__|__|

ASK D22-D23 AS INDICATED FOR EACH SURGERY REPORTED IN D21.

D21. Did (you/_____) have (SURGERY)? YES NO DK	D22. What was the date of the surgery?	D23. What was the name and province of the hospital where the surgery was done?
a. Surgery to remove (your/_____'s) gallbladder? .... 1 2 8 |__|	|__|__| - |__|__| - |__|__| YR MO DA<br>DON'T KNOW . 989898	NAME: ____________________<br>PROVINCE: ________________
b. Surgery on (your/_____'s) extrahepatic bile ducts? .... 1 2 8 |__|	|__|__| - |__|__| - |__|__| YR MO DA<br>DON'T KNOW . 989898	NAME: ____________________<br>PROVINCE: ________________
c. Any other surgery involving the gallbladder or extrahepatic bile ducts? .... 1 2 8 |__|	|__|__| - |__|__| - |__|__| YR MO DA<br>DON'T KNOW . 989898	NAME: ____________________<br>PROVINCE: ________________

D24. (Were you/Was _____) put on a specific diet due to gallstones?

YES... 1
NO 2
DON'T KNOW .... 8 |__|

D25. Did a doctor ever prescribe medication, including herbal medications, to (you/___) to dissolve gallstones?

YES 1
NO .... 2 } (D27)
DON'T KNOW .... 8 } (D27) |__|

D26. What medications were prescribed?

____

____

__________________________________ |__|

D27. Did (you/___) ever have a catheter placed directly into the gallbladder and receive an injection of medicine to dissolve gallstones?

YES.. 1
NO 2
DON'T KNOW .... 8 |__|

D28. Did a doctor ever tell (you/___) that (you/_____) had gallbladder diseases other than gallstones?

YES 1
NO .... 2 } (D31)
DON'T KNOW .... 8 } |__|

ASK D30 AS INDICATED FOR EACH DISEASE REPORTED IN D29.

D29. What type of gallbladder disease did (you/_____) have? Did (you/_____) have (DISEASE)?	YES	NO	DK		D30. How old (were you/was _____) when (you were/_____ was) first told that (you/_____) had this disease? AGE	DK	
a. Inflammation?	1	2	8	|__|	|__|__|	98	|__|__|
b. Infection?.....................................	1	2	8	|__|	|__|__|	98	|__|__|
c. Acute or chronic cholecystitis?................................	1	2	8	|__|	|__|__|	98	|__|__|
d. Any other gallbladder disease? .... (SPECIFY: ______________________________	1	2	8	|__|	|__|__|	98	|__|__|

ASK D32 AS INDICATED FOR EACH DISEASE REPORTED IN D31.

D31. Did a doctor ever tell (you/_____) that (you/_____) had (DISEASE)?	YES	NO	DK		D32. How old (were you/was _____) when (you were/_____ was) first told that (you/_____) had this disease? AGE	DK	
a. Typhoid fever?	1	2	8	|__|	|__|__|	98	|__|__|
b. Paratyphoid fever?........................	1	2	8	|__|	|__|__|	98	|__|__|

D33. Did a doctor ever tell (you/___) that (you were/he/she was) a chronic carrier of the typhoid germ?

YES.. 1
NO 2
DON'T KNOW .... 8 |__|

D34. Did a doctor ever tell (you/_____) that (you/she/he) had diabetes?

YES 1
NO .... 2 } (SKIP TO D47)
DON'T KNOW .... 8 } |__|

D35. How old (were you/was _____) when the doctor first told (you/_____) that (you/_____) had diabetes?

|__|__|
AGE
DON'T KNOW .... 98 |__|__|

D36. What type of diabetes did the doctor tell (you/_____) that (you/_____) had when you were first diagnosed?

INSULIN-DEPENDENT (TYPE I)... 1
INSULIN-INDEPENDENT (TYPE II) 2
PREGNANCY OR GESTATIONAL... 3
DON'T KNOW... 8 |__|

D37. (Were you/Was ___) ever treated with insulin?

YES 1
NO .... 2 } (D40)
DON'T KNOW .... 8 } |__|

D38. At what age (were you/was ____) first given insulin?

|__|__|
AGE
DON'T KNOW .... 98 |__|__|

D39. How many years in total (were you/was _____) treated with insulin?

|__|__|
YEARS
DON'T KNOW .... 98 |__|__|

D40. (Were you/Was _____) ever treated with pills for diabetes?

YES 1
NO .... 2 } (D42)
DON'T KNOW .... 8 } |__|

D41. What specific pills (were you/was _____) treated with? (CIRCLE ALL THAT APPLY)

GLIBENCLAMIDE .... 1 |__|
TOLLOUTUMIDE (D860) .... 2 |__|
GLIPIZIDE.. 3 |__|
DIAMICROS... 4 |__|
PHENFORMIN (DB1) .... 5 |__|
METHORMIN (DIAFORMIN) .... 6 |__|
OTHER (SPECIFY) .... 7 |__|
____
DON'T KNOW .... 8 |__|

D42. (Were you/Was _____) ever hospitalized for complications or treatment of diabetes?

YES 1
NO .... 2 } (D45)
DON'T KNOW .... 8 } |__|

D43. How many times (were you/was _____) hospitalized for complications or treatment of diabetes?

|__|__|
TIMES
DON'T KNOW .... 98 |__|__|

D44. If hospitalized, please list name and province of hospitals, and year of all hospitalizations.

NAME	PROVINCE	YEAR
____________	____________	19 |__|__|
____________	____________	19 |__|__|
____________	____________	19 |__|__|
____________	____________	19 |__|__|
____________	____________	19 |__|__|

ASK D46 AS INDICATED FOR EACH COMPLICATION REPORTED IN D45.

D45. Did a doctor ever tell (you/_____) that (you/_____) had complications of diabetes involving the (ORGAN/CONDITION)?	YES	NO	DK		D46. Did (you/_____) require hospitalization for this condition? YES	NO	DK	
a. Eye?..	1	2	8	|__|	1	2	8	|__|
b. Kidney?	1	2	8	|__|	1	2	8	|__|
c. Circulatory system?......................	1	2	8	|__|	1	2	8	|__|
d. Nervous system?	1	2	8	|__|	1	2	8	|__|
e. Acidosis or ketoacidosis?	1	2	8	|__|	1	2	8	|__|
f. Infections related to diabetes?	1	2	8	|__|	1	2	8	|__|
g. Coma?	1	2	8	|__|	1	2	8	|__|

ASK D48-D50, AS INDICATED, FOR EACH SURGERY REPORTED IN D47.

D47. Did (you/_____) ever have (SURGERY)?	YES	NO	DK		D48. For what condition?	D49. What was the year of the first surgery?	D50. What is the name and province of the hospital where (you/_____) had the surgery?
a. Complete or partial gastrectomy?`	1	2	8	|__|	____________	|__|__| YR DK 98	____________ ____________
b. Surgery on the small bowel?	1	2	8	|__|	____________	|__|__| YR DK 98	____________ ____________

c. Surgery on the large bowel?	1	2	8	|__|	____________	|__|__| YR<br>DK 98	____________________<br>____________________
d. Surgery on the liver?	1	2	8	|__|	____________	|__|__| YR<br>DK 98	____________________<br>____________________

D51. Before one year ago, did a doctor ever tell (you/_____) that (you/_____) had any thyroid disease?

YES 1
NO .... 2 } (D55)
DON'T KNOW .... 8 } (D55) |__|

D52. What was the specific type of thyroid disease that (you were/____________ was) diagnosed with?

GRAVE'S DISEASE (HYPERTHYROIDISM) 1
HASHIMOTO'S DISEASE 2
HYPOTHYROIDISM 3
BENIGN ADENOMA OR NODULE 4
THYROID CARCINOMA 5
GOITER 6
DON'T KNOW .... 8 |__|

D53. How old (were you/was _____) when the doctor first told (you/_____) that (you/_____) had a thyroid disease?

|__|__|
AGE
DON'T KNOW .... 98 |__|__|

D54. What type of treatment did (you/_____) receive for the thyroid disease? Did (you/_____) receive (TREATMENT) . . .

	YES	NO	DK	
a. Prescribed medicine?	1	2	8	|__|
b. Surgery?	1	2	8	|__|
c. Radiation?	1	2	8	|__|

D55. Before one year ago, did a doctor ever tell (you/_____) that (you/_____) had cancer or a tumor?

YES 1
NO .... 2 } (D59)
DON'T KNOW .... 8 } (D59) |__|

D56. How old (were you/was ______) when a doctor first told (you/_____) this?

|__|__|
AGE
DON'T KNOW .... 98 |__|__|

D57. What part of (your/_____'s) body was affected, that is, where was this cancer or tumor located?

____

D58. What was the name and province of the hospital where the cancer or tumor was diagnosed?

NAME: ____________________________

PROVINCE: ________________________

ASK D60-D63, AS INDICATED, FOR EACH MEDICATION REPORTED IN D59.

D59. Did (you/______) ever use pills or shots containing (MEDICINE)? YES NO DK	D60. For what condition did (you/_____) take (MEDICINE)?	D61. At what age did (you/_____) start taking (MEDICINE)?	D62. At what age did (you/_____) last take (MEDICINE)?	D63. In total, how many days, weeks, months, or years did (you/_____) take (MEDICINE)?
a. Cortisone .... 1 2 8 |__|	______________ ______________	|__|__| AGE DK............ 98	|__|__| AGE DK............98	|__|__| # DAYS...... 1 WEEKS... 2 MONTHS 3 YEARS.... 4 DK........... 8 |__|
b. Male hormones ... 1 2 8 |__|	______________ ______________	|__|__| AGE DK............ 98	|__|__| AGE DK............98	|__|__| # DAYS...... 1 WEEKS... 2 MONTHS 3 YEARS.... 4 DK........... 8 |__|
c. Any ulcer medication .... 1 2 8 |__| SPECIFY _______________	______________ ______________	|__|__| AGE DK............ 98	|__|__| AGE DK............98	|__|__| # DAYS...... 1 WEEKS... 2 MONTHS 3 YEARS.... 4 DK........... 8 |__|

D64. Before one year ago, did (you/_____) ever take any aspirin or aspirin-containing products such as Bayer aspirin or Bufferin on a regular basis, that is, at least 2 or more times a week for one month or longer?

YES 1
NO .... 2 } (D69)
DON'T KNOW .... 8 } (D69) |__|

D65. How old (were you/was _____) when (you/_____) started taking aspirin or aspirin-containing products on a regular basis?

|__|__|
AGE
DON'T KNOW .... 98 |__|__|

D66. How old (were you/was _____) when (you/_____) stopped taking aspirin or aspirin-containing products on a regular basis?

|__|__|
AGE
STILL TAKING PRODUCTS 00
DON'T KNOW .... 98 |__|__|

D67. How many pills per day or week did (you/_____) usually take?

|__|__| # PILLS
PER DAY 1
PER WEEK 2
DON'T KNOW .... 988 |__|__|__|

D68. In total, for how many months or years did (you/_____) take aspirin or aspirin-containing products on a regular basis?

|__|__| #
MONTHS 1
YEARS 2
DON'T KNOW .... 988 |__|__|__|

D69. As an adult, would you say (your/______'s) facial skin was: (READ)

Oily, ... 1
Dry, ... 2
A combination, or 3
Normal? 4
DON'T KNOW .... 8 |__|

D70. As an adult, did (you/______) have severe chronic acne?

YES 1
NO .... 2 } (D73)
DON'T KNOW .... 8 } (D73) |__|

D71. Did (you/______) ever take medication for acne?

YES 1
NO .... 2 } (D73)
DON'T KNOW .... 8 } (D73) |__|

D72. In total, how many days, weeks, months or years did (you/_____) take medication for acne?

|__|__| #
DAYS 1
WEEKS.. 2
MONTHS 3
YEARS .... 4 |__|
DON'T KNOW 988

D73. Would you say (your/______'s) earwax (is/was): (READ)

Dry, brittle and gray, or	1	
Wet, sticky and yellowish brown?	2	
DON'T KNOW	8	|__|

D74. As an adult, would you say (your/______'s) hair (is/was): (READ)

Oily, ...	1	
Dry, ...	2	
A combination, or	3	
Normal?	4	
DON'T KNOW	8	|__|

D75. As an adult, do you think (you/______) sweat more or less than the average (man/woman)?

MORE..	1	
LESS	2	
SAME	3	
DON'T KNOW	8	|__|

D76. In the summertime, (do your/did ______'s) palms sweat a lot?

YES..	1	
NO	2	
DON'T KNOW	8	|__|

D77. In the summertime, how often (do you/did ______) use handkerchiefs to wipe sweat off (your/his/her) face? Would you say: (READ)

Frequently, ...	1	
Sometimes, ...	2	
Seldom, or..	3	
Never?..	4	
DON'T KNOW	8	|__|

D78. Compared to other (men/women) (your/his/her) age, would you say (your/______'s) body odor (is/was): (READ)

Stronger, ...	1	
About the same, or.......................................	2	
Less strong?	3	
DON'T KNOW	8	|__|

D79. As an adult, did (you/______) use deodorant?

YES	1	
NO	2	
DON'T KNOW	8	|__|

SECTION E. DIET HISTORY

I'd like to ask some questions about (your/_____'s) usual dietary habits during (your/_____'s) adult years before 5 years ago. If (you have/_____ has) had a change in (your/his/her) dietary habits in the past 5 years, please do not include these changes. The first questions ask about the way (your/_____'s) family prepared foods. Again, please think about what (you/_____) usually ate as an adult before 5 years ago. If (you/_____) did not eat a particular type of food I ask about, please tell me.

E1. Before 5 years ago, as an adult, how often did (you/_____) eat (FOOD)?

	NEVER	LESS THAN ONCE A MONTH	1-3 TIMES A MONTH	ONCE A WEEK OR MORE	DK	
1. Deep fried foods	1	2	3	4	8	|__|
2. Smoked foods	1	2	3	4	8	|__|
3. Cured foods	1	2	3	4	8	|__|
4. Grilled foods	1	2	3	4	8	|__|

E2. Please look at this card and tell me, before 5 years ago, as an adult, when (you/_____) ate meat, how was it usually cooked? (CODE ALL THAT APPLY.)

SHOW CARD A

STIR FRIED	01		|__|__|
PAN FRIED	02		|__|__|
BOILED/STEWED	03		|__|__|
STEAMED	04		|__|__|
ROASTED	05		|__|__|
DEEP FRIED	06		|__|__|
OTHER (SPECIFY) __________________ ________________________________	96		|__|__|
DON'T KNOW	98	} (E7)	|__|__|
DID NOT EAT MEAT	00	} (E7)	|__|__|

E3. (IF MORE THAN ONE METHOD CIRCLED IN E2, ASK:) Which of these methods (READ) was used <u>most often</u>? (CIRCLE ONE ONLY)

STIR FRIED	01	
PAN FRIED	02	
BOILED/STEWED	03	
STEAMED	04	
ROASTED	05	
DEEP FRIED	06	
OTHER (SPECIFY) __________________ ________________________________	96	
DON'T KNOW	98	|__|__|

E4. Before 5 years ago, as an adult, when (you/_____) ate beef or pork, did (you/_____) usually eat the fat or not?

YES	1	
NO	2	
DID NOT EAT BEEF/PORK	0	
DON'T KNOW	8	|__|

E5. Before 5 years ago, as an adult, how many times per day, week, month, or year did (you/_____) usually eat vegetables cooked with salt pork, ham hocks, bacon fat, or fat-back?

	PER DAY	1	
|___|___|	PER WEEK	2	
NUMBER	PER MONTH	3	
	PER YEAR	4	
	NEVER	000	
	DON'T KNOW	988	|__|__|__|

E6. Before 5 years ago, as an adult, how many times per day, week, month, or year did (you/_____) usually eat meat drippings mixed with rice?

	PER DAY	1	
|___|___|	PER WEEK	2	
NUMBER	PER MONTH	3	
	PER YEAR	4	
	NEVER	000	
	DON'T KNOW	988	|__|__|__|

E7. Please look at this card and tell me, before 5 years ago, as an adult, when (you/_____) ate poultry, how was it usually cooked? (CODE ALL THAT APPLY.)

SHOW CARD A

STIR FRIED	01		|__|__|
PAN FRIED	02		|__|__|
BOILED/STEWED	03		|__|__|
STEAMED	04		|__|__|
ROASTED	05		|__|__|
DEEP FRIED	06		|__|__|
OTHER (SPECIFY) ____________________	96		|__|__|
DON'T KNOW	98	(E10)	|__|__|
DID NOT EAT MEAT	00	(E10)	|__|__|

E8. (IF MORE THAN ONE METHOD CIRCLED IN E7, ASK:) Which of these methods (READ) was used most often? (CIRCLE ONE ONLY)

STIR FRIED 01
PAN FRIED 02
BOILED/STEWED 03
STEAMED 04
ROASTED 05
DEEP FRIED 06
OTHER (SPECIFY) __________ 96

____

DON'T KNOW .... 98 |__|__|

E9. Before 5 years ago, as an adult, did (you/______) usually eat poultry with or without the skin?

EAT WITH SKIN 1
EAT WITHOUT SKIN 2
BOTH EQUALLY 3
DON'T KNOW .... 8 |__|

E10. Please look at this card and tell me, before 5 years ago, as an adult, when (you/_____) ate cooked fish, how was it usually cooked? (CODE ALL THAT APPLY.)

SHOW CARD A

STIR FRIED .... 01 |__|__|
PAN FRIED .... 02 |__|__|
BOILED/STEWED .... 03 |__|__|
STEAMED .... 04 |__|__|
ROASTED .... 05 |__|__|
DEEP FRIED .... 06 |__|__|
OTHER (SPECIFY) __________ 96 |__|__|

____

DON'T KNOW .... 98 } (E12) |__|__|
DID NOT EAT MEAT .... 00 } (E12) |__|__|

E11. (IF MORE THAN ONE METHOD CIRCLED IN E10, ASK:) Which of these methods (READ) was used most often? (CIRCLE ONE ONLY)

STIR FRIED 01
PAN FRIED 02
BOILED/STEWED 03
STEAMED 04
ROASTED 05
DEEP FRIED 06
OTHER (SPECIFY) __________ 96

____

DON'T KNOW .... 98 |__|__|

E12. Before 5 years ago, when (you/_____) ate meat, poultry, or fish cooked with vegetables, what was the proportion of meat, poultry, or fish to vegetables? Was it: (READ)

About equal, ... 1
Somewhat more meat, poultry, or fish than vegetables, or 2
Somewhat more vegetables than meat, poultry, or fish? 3
DON'T KNOW 8
DID NOT EAT MEAT/FISH/POULTRY COOKED WITH VEGETABLES 0
|__|

E13. Before 5 years ago, as an adult, how were vegetables usually prepared when (you/_____) ate them by themselves? Were they usually cooked with: (READ)

Water, ... 1
Oil, ... 2
Something else? (SPECIFY) 6
____
DON'T KNOW 8
NEVER EATEN BY THEMSELVES .... 0 |__|

E14. Before 5 years ago, as an adult, what type of milk did (you/_____) usually drink? (DO NOT READ CATEGORIES. PROBE FOR ONE TYPE.)

WHOLE MILK 1
EVAPORATED/CANNED/CONDENSED MILK 2
POWDERED MILK 3
SOYBEAN MILK 4
OTHER (SPECIFY) 6
____
DON'T KNOW 8
DID NOT DRINK MILK .... 0 |__|

In this section I will ask about how many times per day, week, month or year (you/_____) ate certain foods before 5 years ago and how many liang of each food (you/_____) ate. Please be as specific as possible, but if you don't remember exactly, please give me an approximate answer. If a food was available only during certain seasons, please tell me how often (you/_____) ate it in season.

E15. First I have some questions about salted and fermented foods. Before 5 years ago, how many times per day, week, month or year did (you/_____) usually eat (FOOD)?								E16. (IF EATEN): How many liang did (you/ ____) eat each time?	
	1 TIMES PER DAY	2 TIMES PER WEEK	3 TIMES PER MONTH	4 TIMES PER YEAR	5 NEVER	8 DK		# LIANG	DON'T KNOW
1. Salted pork							|__|__|__|	|__|__|__|	
2. Salted fish							|__|__|__|	|__|__|__|	
IF SALTED FISH WAS EVER EATEN, ASK<br>2a. What kind of salted fish did (you/____________) eat most often? ____________________________________									

3. Salted vegetables							|__|__|__|	|__|__|__|	
4. Hot pickled mustard tuber							|__|__|__|	|__|__|__|	
5. Fermented bean curd							|__|__|__|	|__|__|__|	
6. Salted duck eggs							|__|__|__|	|__|__|__|	
7. Preserved eggs							|__|__|__|	|__|__|__|	

E17. Now I will ask you about what kinds of fresh meats, poultry, and fish (you/_____) may have eaten. Before 5 years ago, how many times per day, week, month or year did (you/_____) usually eat (FOOD)?								E18a. (IF EATEN): How many liang did (you/ ____) eat each time?		E18b. Is that weight before or after cooking?
	1	2	3	4	5	8				
	TIMES PER DAY	TIMES PER WEEK	TIMES PER MONTH	TIMES PER YEAR	NEVER	DK		# LIANG	DK	BEFORE AFTER
1. Pork chops							|__|__|__|	|__|__|__|		1 2 |__|
2. Spareribs							|__|__|__|	|__|__|__|		1 2 |__|
3. Pig feet							|__|__|__|	|__|__|__|		1 2 |__|
4. Fresh pork, fat							|__|__|__|	|__|__|__|		1 2 |__|
5. Fresh pork, lean							|__|__|__|	|__|__|__|		1 2 |__|
6. Fresh pork, fat and lean							|__|__|__|	|__|__|__|		1 2 |__|
7. Liver from pork or chicken							|__|__|__|	|__|__|__|		1 2 |__|
8. Other organ meats including heart, brains, tongue, stomach, kidney, intestines							|__|__|__|	|__|__|__|		1 2 |__|
9. Beef, lamb or mutton							|__|__|__|	|__|__|__|		1 2 |__|
10. Chicken							|__|__|__|	|__|__|__|		1 2 |__|
11. Duck							|__|__|__|	|__|__|__|		1 2 |__|

E17. Before 5 years ago, how many times per day, week, month or year did (you/_____) usually eat (FOOD)?	1 TIMES PER DAY	2 TIMES PER WEEK	3 TIMES PER MONTH	4 TIMES PER YEAR	5 NEVER	8 DK		E18a. (IF EATEN): How many liang did (you/ ____) eat each time? # LIANG	DK	
12. Saltwater fish							|__|__|__|	|__|__|__|		
13. Fresh water fish, such as silver carp, grass carp, or golden carp							|__|__|__|	|__|__|__|		
14. Rice field eel or Japanese eel							|__|__|__|	|__|__|__|		
15. Shrimp							|__|__|__|	|__|__|__|		
16. Other shellfish, including crab, snail, conch or whelk, squid, sea cucumber, cuttlefish, oysters, mussels, or clams							|__|__|__|	|__|__|__|		
17. Scrambled or fried poultry eggs							|__|__|__|	|__|__|__|		
18. Raw, steamed, poached, or boiled poultry eggs							|__|__|__|	|__|__|__|		
19. Fresh whole milk							|__|__|__|	|__|__|__|		
20. Ice cream							|__|__|__|	|__|__|__|		
21. Soybean milk							|__|__|__|	|__|__|__|		
22. Deep fried bean curd							|__|__|__|	|__|__|__|		
23. Fresh bean curd (tofu)							|__|__|__|	|__|__|__|		
24. Other soybean foods such as low water content bean curd, soybean curd sheet, vegetarian chicken							|__|__|__|	|__|__|__|		
25. Dry mung beans							|__|__|__|	|__|__|__|		
26. Dry soybeans							|__|__|__|	|__|__|__|		
27. Dry red beans							|__|__|__|	|__|__|__|		

E17. Before 5 years ago, how many times per day, week, month or year did (you/_____) usually eat (FOOD)?	1 TIMES PER DAY	2 TIMES PER WEEK	3 TIMES PER MONTH	4 TIMES PER YEAR	5 NEVER	8 DK		E18a. (IF EATEN): How many liang did (you/ ____) eat each time? # LIANG	DK	
28. Wheat gluten							|__|__|__|	|__|__|__|		
29. Peanut butter							|__|__|__|	|__|__|__|		
30. Peanuts							|__|__|__|	|__|__|__|		
31. Butter							|__|__|__|	|__|__|__|		
32. Margarine							|__|__|__|	|__|__|__|		

E19. Now I would like to ask you about some staple foods (you/_____) may have eaten. Before 5 years ago, how many times per day, week, month, or year did (you/_____) usually eat (FOOD)?	1 TIMES PER DAY	2 TIMES PER WEEK	3 TIMES PER MONTH	4 TIMES PER YEAR	5 NEVER	8 DK		E20a. (IF EATEN): How many liang did (you/ ____) eat each time? # LIANG	DK	E20b. Is that weight <u>before</u> or <u>after</u> cooking? BEFORE AFTER
1. White rice, steamed or in congee							|__|__|__|	|__|__|__|		1 2 |__|
2. Noodles							|__|__|__|	|__|__|__|		1 2 |__|
3. Steamed buns							|__|__|__|	|__|__|__|		
4. Dumplings, steamed or boiled							|__|__|__|	|__|__|__|		
5. Fried dumplings							|__|__|__|	|__|__|__|		

<table>
<tr><td colspan="8">E21.
Now I would like to ask you about some vegetables (you/_____) may have eaten. Before 5 years ago, how many times per day, week, month, or year did (you/_____) usually eat (FOOD)?</td><td colspan="2">E22.
(IF EATEN): How many liang did (you/____) eat each time?</td></tr>
<tr><td></td><td>1
TIMES PER DAY</td><td>2
TIMES PER WEEK</td><td>3
TIMES PER MONTH</td><td>4
TIMES PER YEAR</td><td>5
NEVER</td><td>8
DK</td><td></td><td># LIANG</td><td>DON'T KNOW</td></tr>
<tr><td>1. Bok choy, napa</td><td></td><td></td><td></td><td></td><td></td><td></td><td>|__|__|__|</td><td>|__|__|__|</td><td></td></tr>
<tr><td>2. Spinach</td><td></td><td></td><td></td><td></td><td></td><td></td><td>|__|__|__|</td><td>|__|__|__|</td><td></td></tr>
<tr><td>3. Common cabbage</td><td></td><td></td><td></td><td></td><td></td><td></td><td>|__|__|__|</td><td>|__|__|__|</td><td></td></tr>
<tr><td>4. Chinese cabbage (Shanghai)</td><td></td><td></td><td></td><td></td><td></td><td></td><td>|__|__|__|</td><td>|__|__|__|</td><td></td></tr>
<tr><td>5. Swamp cabbage, ung choy, water spinach</td><td></td><td></td><td></td><td></td><td></td><td></td><td>|__|__|__|</td><td>|__|__|__|</td><td></td></tr>
<tr><td>6. Rape, Chinese flowering cabbage, "olive" cabbage</td><td></td><td></td><td></td><td></td><td></td><td></td><td>|__|__|__|</td><td>|__|__|__|</td><td></td></tr>
<tr><td>7. Fresh mustard greens</td><td></td><td></td><td></td><td></td><td></td><td></td><td>|__|__|__|</td><td>|__|__|__|</td><td></td></tr>
<tr><td>8. Cauliflower</td><td></td><td></td><td></td><td></td><td></td><td></td><td>|__|__|__|</td><td>|__|__|__|</td><td></td></tr>
<tr><td>9. Celery</td><td></td><td></td><td></td><td></td><td></td><td></td><td>|__|__|__|</td><td>|__|__|__|</td><td></td></tr>
<tr><td>10. Flat cabbage</td><td></td><td></td><td></td><td></td><td></td><td></td><td>|__|__|__|</td><td>|__|__|__|</td><td></td></tr>
<tr><td>11. Chinese cabbage seedlings</td><td></td><td></td><td></td><td></td><td></td><td></td><td>|__|__|__|</td><td>|__|__|__|</td><td></td></tr>
<tr><td>12. Mung bean sprouts</td><td></td><td></td><td></td><td></td><td></td><td></td><td>|__|__|__|</td><td>|__|__|__|</td><td></td></tr>
<tr><td>13. Soybean sprouts</td><td></td><td></td><td></td><td></td><td></td><td></td><td>|__|__|__|</td><td>|__|__|__|</td><td></td></tr>
<tr><td>14. Green beans (knife)</td><td></td><td></td><td></td><td></td><td></td><td></td><td>|__|__|__|</td><td>|__|__|__|</td><td></td></tr>
<tr><td>15. Hyacinth beans (flat)</td><td></td><td></td><td></td><td></td><td></td><td></td><td>|__|__|__|</td><td>|__|__|__|</td><td></td></tr>
<tr><td>16. Peas</td><td></td><td></td><td></td><td></td><td></td><td></td><td>|__|__|__|</td><td>|__|__|__|</td><td></td></tr>
<tr><td>17. Snow pea pods</td><td></td><td></td><td></td><td></td><td></td><td></td><td>|__|__|__|</td><td>|__|__|__|</td><td></td></tr>
<tr><td>18. Cow peas</td><td></td><td></td><td></td><td></td><td></td><td></td><td>|__|__|__|</td><td>|__|__|__|</td><td></td></tr>
<tr><td>19. Fresh fava beans (broad beans)</td><td></td><td></td><td></td><td></td><td></td><td></td><td>|__|__|__|</td><td>|__|__|__|</td><td></td></tr>
<tr><td>20. Eggplant</td><td></td><td></td><td></td><td></td><td></td><td></td><td>|__|__|__|</td><td>|__|__|__|</td><td></td></tr>
<tr><td>21. Wild rice stems</td><td></td><td></td><td></td><td></td><td></td><td></td><td>|__|__|__|</td><td>|__|__|__|</td><td></td></tr>
<tr><td>22. Fresh soybeans (yellow)</td><td></td><td></td><td></td><td></td><td></td><td></td><td>|__|__|__|</td><td>|__|__|__|</td><td></td></tr>
<tr><td>23. Snow pea seedlings (leaves)</td><td></td><td></td><td></td><td></td><td></td><td></td><td>|__|__|__|</td><td>|__|__|__|</td><td></td></tr>
</table>

24. Winter melon, wax gourd							|__|__|__|	|__|__|__|	

E21. Before 5 years ago, how many times per day, week, month, or year did (you/_____) usually eat (FOOD)?	1 TIMES PER DAY	2 TIMES PER WEEK	3 TIMES PER MONTH	4 TIMES PER YEAR	5 NEVER	8 DK		E22. (IF EATEN): How many liang did (you/____) eat each time? # LIANG	DON'T KNOW
25. Celtuce							|__|__|__|	|__|__|__|	
26. Lettuce							|__|__|__|	|__|__|__|	
27. Red sweet potatoes or yams							|__|__|__|	|__|__|__|	
28. White sweet potatoes or yams							|__|__|__|	|__|__|__|	
29. White potatoes							|__|__|__|	|__|__|__|	
30. Bottle gourd							|__|__|__|	|__|__|__|	
31. Cucumber							|__|__|__|	|__|__|__|	
32. Carrot							|__|__|__|	|__|__|__|	
33. Pumpkin							|__|__|__|	|__|__|__|	
34. Shiitake mushrooms							|__|__|__|	|__|__|__|	
35. Fresh button mushrooms							|__|__|__|	|__|__|__|	
36. Wood-ear fungus							|__|__|__|	|__|__|__|	
37. Green sweet pepper							|__|__|__|	|__|__|__|	
38. Tomatoes							|__|__|__|	|__|__|__|	
39. Fresh bamboo shoots							|__|__|__|	|__|__|__|	
40. Radish							|__|__|__|	|__|__|__|	
41. Turnip, rutabaga							|__|__|__|	|__|__|__|	
42. Lotus root							|__|__|__|	|__|__|__|	
43. Taro root							|__|__|__|	|__|__|__|	
44. Luffa, sponge or rag gourd							|__|__|__|	|__|__|__|	
45. Garlic							|__|__|__|	|__|__|__|	
46. Onions							|__|__|__|	|__|__|__|	
47. Spring onions, scallions							|__|__|__|	|__|__|__|	
48. Chinese chives							|__|__|__|	|__|__|__|	
49. Garlic stalk							|__|__|__|	|__|__|__|	
50. Hot pepper, chili							|__|__|__|	|__|__|__|	
51. Ginger root							|__|__|__|	|__|__|__|	
52. Seaweed							|__|__|__|	|__|__|__|	

53. Kelp							|__|__|__|	|__|__|__|	

	E23. Now I would like to ask you about some fruits (you/_____) may have eaten. Before 5 years ago, how many times per day, week, month, or year did (you/_____) usually eat (FOOD)?							E24a. (IF EATEN): How many liang did (you/ ____) eat each time?		E24b. Does that weight include the skin or peel?
	1	2	3	4	5	8				
	TIMES PER DAY	TIMES PER WEEK	TIMES PER MONTH	TIMES PER YEAR	NEVER	DK		# LIANG	DK	YES NO
1. Apples							|__|__|__|	|__|__|__|		
2. Pears							|__|__|__|	|__|__|__|		
3. Oranges or tangerines							|__|__|__|	|__|__|__|		1 2 |__|
4. Bananas							|__|__|__|	|__|__|__|		1 2 |__|
5. Grapes							|__|__|__|	|__|__|__|		
6. Peaches							|__|__|__|	|__|__|__|		
7. Watermelon							|__|__|__|	|__|__|__|		1 2 |__|
8. Cantaloupe							|__|__|__|	|__|__|__|		1 2 |__|

	E25. Now I would like to ask you about some desserts (you/_____) may have eaten. Before 5 years ago, how many times per day, week, month, or year did (you/_____) usually eat (FOOD)?							E26. (IF EATEN): How many liang did (you/ ____) eat each time?	
	1	2	3	4	5	8			
	TIMES PER DAY	TIMES PER WEEK	TIMES PER MONTH	TIMES PER YEAR	NEVER	DK		# LIANG	DON'T KNOW
1. Cakes and pastries							|__|__|__|	|__|__|__|	
2. Sweet bread							|__|__|__|	|__|__|__|	
3. Cookies							|__|__|__|	|__|__|__|	
4. Red bean soup							|__|__|__|	|__|__|__|	
5. Green bean soup							|__|__|__|	|__|__|__|	

E27. Before 5 years ago, how many liang of (FOOD) did (you/_____) eat per month?

	# LIANG PER MONTH	DON'T KNOW	
1. Sugar	|__|__|	98	|__|__|
2. Salt	|__|__|	98	|__|__|
3. Soy sauce	|__|__|	98	|__|__|

E28. Now I will ask about how many liang of certain oils (you/____) ate each month. Before 5 years ago, how many liang of (FOOD) did (you/_____) eat per month?

	# LIANG PER MONTH	DON'T KNOW	
1. Rapeseed oil	|__|__|	98	|__|__|
2. Soybean oil	|__|__|	98	|__|__|
3. Peanut oil	|__|__|	98	|__|__|
4. Lard	|__|__|	98	|__|__|
5. Sesame oil	|__|__|	98	|__|__|

E29. Now I would like to ask you about certain fried foods (you/_____) may have eaten. Before 5 years ago, how many times per day, week, month, or year did (you/_____) usually eat (FOOD)?	1 TIMES PER DAY	2 TIMES PER WEEK	3 TIMES PER MONTH	4 TIMES PER YEAR	5 NEVER	8 DK		E30. (IF EATEN): How many liang did (you/ ____) eat each time? # LIANG	DON'T KNOW
1. Fried chicken							|__|__|__|	|__|__|__|	
2. Breaded fried fish (batter-fish)							|__|__|__|	|__|__|__|	
3. Breaded fried pork chops (batter-pork)							|__|__|__|	|__|__|__|	
4. Spring rolls							|__|__|__|	|__|__|__|	
5. Deep fried dough stick							|__|__|__|	|__|__|__|	

E31. Now I'd like you to think about (your/_____'s) diet over the past 5 years and compare it to (your/_____'s) usual diet before 5 years ago. Did (your/_____'s) diet change significantly over the past 5 years?

YES 1
NO .... 2 (E35) |__|

E32. How did (your/______'s) diet change over the past 5 years? (CIRCLE ALL THAT APPLY)

EAT MORE VEGETABLES NOW .... 1 |__|
EAT MORE MEAT NOW .... 2 |__|
EAT MORE FRUITS NOW .... 3 |__|
EAT LESS VEGETABLES NOW .... 4 |__|
EAT LESS MEAT NOW .... 5 |__|
EAT LESS FRUIT NOW .... 6 |__|
OTHER (SPECIFY) .... 7 |__|
____
DON'T KNOW .... 8 |__|

E33. Why did (your/______'s) diet change over the past 5 years?

DOCTOR'S RECOMMENDATION........... 1
SWITCH TO A MORE HEALTHY DIET... 2
NOT AS HUNGRY 3
MORE HUNGRY.. 4
OTHER (SPECIFY) 5
____
DON'T KNOW .... 8 |__|

E34. In what year did (your/____'s) diet change? 19|__|__|
YEAR
DON'T KNOW .... 98 |__|__|

Next I'd like to ask about (your/____'s) use of vitamins.

E35. During (your/____'s) adult life, did (you/he/she) take (VITAMIN) at least once a week for one year or more:	E36. How many years did (you/he/she) take (VITAMIN) regularly?	E37. Not counting the last 12 months, how many times per day or week did (you/he/she) usually take (VITAMIN)?
a. Multivitamin pills, such as One-A-Day or Theragran? YES........... 1 → NO .... 2 (b) DK .... 8 |__| a.1. IF YES: What was the name of the multi-vitamin that (you/he/she) usually took? ____________________________	|___|___| YEARS DON'T KNOW............ 98	|__|__| NO. PER DAY....... 1 PER WEEK ... 2 |__| DK 988
b. Vitamin A capsules? YES .... 1 → NO .... 2 (c) DK .... 8 |__|	|___|___| YEARS DON'T KNOW............ 98	|__|__| NO. PER DAY....... 1 PER WEEK ... 2 |__| DK 988
c. Vitamin B complex? YES .... 1 → NO .... 2 (d) DK .... 8 |__|	|___|___| YEARS DON'T KNOW............ 98	|__|__| NO. PER DAY....... 1 PER WEEK ... 2 |__| DK 988
d. Vitamin E? YES .... 1 → NO .... 2 (e) DK .... 8 |__|	|___|___| YEARS DON'T KNOW............ 98	|__|__| NO. PER DAY....... 1 PER WEEK ... 2 |__| DK 988
e. Folic acid? YES .... 1 → NO .... 2 (g) DK .... 8 |__|	|___|___| YEARS DON'T KNOW............ 98	|__|__| NO. PER DAY....... 1 PER WEEK ... 2 |__| DK 988
f. Vitamin C pills? YES .... 1 → NO .... 2 (h) DK .... 8 |__|	|___|___| YEARS DON'T KNOW............ 98	|__|__| NO. PER DAY....... 1 PER WEEK ... 2 |__| DK 988
g. Cod liver oil? YES .... 1 → NO .... 2 (i) DK .... 8 |__|	|___|___| YEARS DON'T KNOW............ 98	|__|__| NO. PER DAY....... 1 PER WEEK ... 2 |__| DK 988
h. EPA (Omega-3 fish oil? YES .... 1 → NO .... 2 (j) DK .... 8 |__|	|___|___| YEARS DON'T KNOW............ 98	|__|__| NO. PER DAY....... 1 PER WEEK ... 2 |__| DK 988

E35.<br>During (your/____'s) adult life, did (you/he/she) take any of the following at least once a week for one year or more:	E36.<br>How many years did (you/he/she) take (VITAMIN) regularly?	E37.<br>Not counting the last 12 months, how many times per day or week did (you/he/she) usually take (VITAMIN)?
i. Selenium?<br>YES .... 1 →<br>NO .... 2 (k)<br>DK .... 8 |__|	|___|___| YEARS<br>DON'T KNOW 98	|__|__| PER DAY .... 1<br>NO. PER WEEK ... 2 |__| DK 988
j. Zinc?<br>YES .... 1 →<br>NO .... 2 (l)<br>DK .... 8 |__|	|___|___| YEARS<br>DON'T KNOW 98	|__|__| PER DAY .... 1<br>NO. PER WEEK ... 2 |__| DK 988
k. Garlic supplement?<br>YES .... 1 →<br>NO .... 2 (m)<br>DK .... 8 |__|	|___|___| YEARS<br>DON'T KNOW 98	|__|__| PER DAY .... 1<br>NO. PER WEEK ... 2 |__| DK 988

SECTION F. MENSTRUATION AND MENOPAUSE HISTORY

BOX F-1
COMPLETE SECTION G FOR FEMALE SUBJECTS ONLY.
SKIP TO SECTION K FOR MALE SUBJECTS.

The next questions are about (your/_____'s) menstrual periods and surgery that (you/_____) may have had.

F1. At what age did (you/_____) have (your/her) first menstrual period?

AGE: |___|___|
NEVER 00 (F6)
DON'T KNOW .... 98 |__|__|

F2. The next question is about when (your/_____'s) menstrual periods became regular. "Regular" means your periods come about once a month, you can usually predict when they will come plus or minus 4 days, and each time they last about the same number of days. At what age did (your/_____'s) menstrual periods become established at regular intervals?

AGE: |___|___|
NEVER BECAME REGULAR 00 (F4)
DON'T KNOW.. 98 |__|__|

F3. How many days did (you/_____) usually have between the <u>beginning</u> of one period and the <u>beginning</u> of the next? Please do not include any times that (you were/she was) taking birth control pills. (RECORD SINGLE NUMBER OR RANGE OF DAYS.)

OF DAYS: |___|___|.................. TO DAYS: |___|___|
DON'T KNOW.. 9898

F4. During what year and month did (you/_____) have (your/her) most recent period?

19 |___|___| - |___|___|
YEAR MONTH
DON'T KNOW.. 999898

BOX F-2
IF SIX MONTHS OR MORE SINCE MOST RECENT PERIOD, ASK F5.<br>IF LESS THAN SIX MONTHS SINCE MOST RECENT PERIOD, SKIP TO F6.

F5. Is the reason (you have/_____ has) not had a menstrual period since (MO/YR FROM F4) due to natural menopause, surgery, (a recent pregnancy) or some other reason?

NATURAL MENOPAUSE (CHANGE OF LIFE)	1	
SURGERY (HYSTERECTOMY AND/OR REMOVAL OF OVARIES)	2	
RECENT PREGNANCY	3	
OTHER (SPECIFY) ____	6	
DON'T KNOW	8	|__|

F6. Did (you/_______) ever have any surgery involving removal, either partial or total, of: (READ)

	YES	NO	DK	
One ovary?..	1	2	8	|__|
Both ovaries?	1	2	8	|__|
The uterus?	1	2	8	|__|

SECTION G. CONTRACEPTIVE HISTORY

BOX G-1
COMPLETE SECTION G FOR FEMALE SUBJECTS ONLY.
SKIP TO SECTION K FOR MALE SUBJECTS.

Now we'll talk about any birth control or methods of family planning (you/_____) and a partner have ever used.

G1. First, did (you/_____) and a partner ever use a method of birth control for family planning?

YES 1
NO .... 2 } (G6)
DON'T KNOW .... 8 } |__|

G2. Please look at this card and tell me which of these methods of birth control (you/_____) and a partner ever used. (CIRCLE CODES OF ALL METHODS USED.)

SHOW CARD B

CODES	METHOD	
01	BIRTH CONTROL PILL OR ORAL CONTRACEPTIVES	|__|__|
02	INJECTIONS (DEPO-PROVERA)	|__|__|
03	NORPLANT	|__|__|
04	MORNING-AFTER PILL OR SHOTS	|__|__|
05	OPERATION - FEMALE STERILIZATION, TUBES TIED	|__|__|
06	OPERATION - MALE STERILIZATION, VASECTOMY	|__|__|
07	INTRAUTERINE DEVICE (IUD)	|__|__|
08	NONE OF THESE METHODS	|__|__|
98	DON'T KNOW.....|__|__|	

**BOX G-2**
IF SUBJECT EVER USED BIRTH CONTROL PILLS (CODE 01 IN G2 IS CIRCLED), CONTINUE WITH G3.
IF SUBJECT NEVER USED BIRTH CONTROL PILLS, SKIP TO G6.

BIRTH CONTROL PILLS

You mentioned that (you/_____) had used birth control pills. Now I'd like to record which pills (you have/_____ has) taken.

	G3.<br>At what age did (you/_____) (first/ next) start using birth control pills?	G4.<br>What is the name of the (first/next) pill (you/_____) started using when you were (AGE in G3)? (ENTER COMPLETE NAME AND CODE.)	G5.<br>At what age did (you/_____) stop taking (PILL)?
1ST PILL USE	|__|__| AGE<br>DK....................98	NAME: _____________<br>DON'T KNOW .... 98<br>|__|__|	|__|__| AGE<br>DK 98
2ND PILL USE	|__|__| AGE<br>DK....................98	NAME: _____________<br>DON'T KNOW .... 98<br>|__|__|	|__|__| AGE<br>DK 98
3RD PILL USE	|__|__| AGE<br>DK....................98	NAME: _____________<br>DON'T KNOW .... 98<br>|__|__|	|__|__| AGE<br>DK 98
4TH PILL USE	|__|__| AGE<br>DK....................98	NAME: _____________<br>DON'T KNOW .... 98<br>|__|__|	|__|__| AGE<br>DK 98
5TH PILL USE	|__|__| AGE<br>DK....................98	NAME: _____________<br>DON'T KNOW .... 98<br>|__|__|	|__|__| AGE<br>DK 98

G6. Sometimes women are given birth control pills for reasons other than birth control; for example, for irregular menstrual periods or acne. Did (you/_____) ever take birth control pills for a reason other than birth control?

YES 1
NO .... 2 } (SEC. H)
DON'T KNOW .... 8 } (SEC. H) |__|

	G7. At what age did (you/_____) (first/next) start using birth control pills for reasons other than birth control?	G8. What is the name of the pill (you/_____) started using when you were (AGE IN G7)? (ENTER COMPLETE NAME AND CODE.)	G9. What was the reason (you/_____) took (PILL)? (CIRCLE ALL THAT APPLY.)	G10. At what age did (you/_____) stop taking (PILL) for (this reason/these reasons)?
1ST PILL USE	|__|__| AGE DK...................98	NAME: _______________ DON'T KNOW.............98 |__|__|	REGULATE PERIODS..1 |__| ACNE ....2 |__| OTHER (SPECIFY)......6 |__| __________________ __________________ DON'T KNOW..............8 |__|	|__|__| AGE DK 98
2ND PILL USE	|__|__| AGE DK...................98	NAME: _______________ DON'T KNOW.............98 |__|__|	REGULATE PERIODS..1 |__| ACNE ....2 |__| OTHER (SPECIFY)......6 |__| __________________ __________________ DON'T KNOW..............8 |__|	|__|__| AGE DK 98
3RD PILL USE	|__|__| AGE DK...................98	NAME: _______________ DON'T KNOW.............98 |__|__|	REGULATE PERIODS..1 |__| ACNE ....2 |__| OTHER (SPECIFY)......6 |__| __________________ __________________ DON'T KNOW..............8 |__|	|__|__| AGE DK 98
4TH PILL USE	|__|__| AGE DK...................98	NAME: _______________ DON'T KNOW.............98 |__|__|	REGULATE PERIODS..1 |__| ACNE ....2 |__| OTHER (SPECIFY)......6 |__| __________________ __________________ DON'T KNOW..............8 |__|	|__|__| AGE DK 98
5TH PILL USE	|__|__| AGE DK...................98	NAME: _______________ DON'T KNOW.............98 |__|__|	REGULATE PERIODS..1 |__| ACNE ....2 |__| OTHER (SPECIFY)......6 |__| __________________ __________________ DON'T KNOW..............8 |__|	|__|__| AGE DK 98

SECTION H. PREGNANCY HISTORY

> BOX H-1
>
> COMPLETE SECTION H FOR FEMALE SUBJECTS ONLY.
>
> SKIP TO SECTION K FOR MALE SUBJECTS.

In this next section, I want to ask you some questions about all of (your/_____'s) pregnancies.

H1. (Have you/Has _____) ever been pregnant?

YES 1
NO .... 2 } (H6)
DON'T KNOW .... 8 } (H6) |__|

H2. What is the total number of pregnancies (you have/_____ has) had, including live births, stillbirths, miscarriages, induced abortions, molar pregnancies, and tubal or other ectopic pregnancies? (INCLUDE A CURRENT PREGNANCY IN THE TOTAL.)

|__|__|
TOTAL PREGNANCIES
DON'T KNOW .... 98 |__|__|

Now, I would like to ask some specific information about (each time (you/_____) became pregnant/(your/_____'s) pregnancy). As we go through, if you remember a pregnancy you forgot to tell me about, please let me know.

	PREG 1	PREG 2
H3. Was the outcome of (your/_____'s) (1st/2nd/etc.) pregnancy, a live birth, stillbirth, miscarriage, abortion, tubal pregnancy, (or) molar pregnancy, (or are you currently pregnant)?		
a. LIVE BIRTH	1	1
b. STILLBIRTH..	.................2 (NEXT PREG OR BOX H-3)	.................2 (NEXT PREG OR BOX H-3)
c. MISCARRIAGE	.................3 (NEXT PREG OR BOX H-3)	.................3 (NEXT PREG OR BOX H-3)
d. INDUCED ABORTION	.................4 (NEXT PREG OR BOX H-3)	.................4 (NEXT PREG OR BOX H-3)
e. TUBAL OR ECTOPIC PREGNANCY	.................5 (NEXT PREG OR BOX H-3)	.................5 (NEXT PREG OR BOX H-3)
f. MOLAR PREGNANCY	.................6 (NEXT PREG OR BOX H-3)	.................6 (NEXT PREG OR BOX H-3)
g. CURRENT PREGNANCY	.................7 (NEXT PREG OR BOX H-3)	.................7 (NEXT PREG OR BOX H-3)
h. DON'T KNOW	.................8 (NEXT PREG OR BOX H-3) |__|	.................8 (NEXT PREG OR BOX H-3) |__|
H4. How many months did (you/_____) breast feed this child?	|__|__| MONTHS<br>NEVER BREAST FED ..00<br>DON'T KNOW 98	|__|__| MONTHS<br>NEVER BREAST FED ..00<br>DON'T KNOW 98

PREG 3	PREG 4	PREG 5	PREG 6	PREG 7
.........1<br>.........2<br>.........3<br>.........4<br>.........5<br>.........6<br>.........7<br>.........8<br>(NEXT PREG OR BOX H-3)<br>|__|	.........1<br>.........2<br>.........3<br>.........4<br>.........5<br>.........6<br>.........7<br>.........8<br>(NEXT PREG OR BOX H-3)<br>|__|	.... 1<br>........ 2<br>........ 3<br>........ 4<br>........ 5<br>........ 6<br>........ 7<br>........ 8<br>(NEXT PREG OR BOX H-3)<br>|__|	.........1<br>.........2<br>.........3<br>.........4<br>.........5<br>.........6<br>.........7<br>.........8<br>(NEXT PREG OR BOX H-3)<br>|__|	.... 1<br>........ 2<br>........ 3<br>........ 4<br>........ 5<br>........ 6<br>........ 7<br>........ 8<br>(NEXT PREG OR BOX H-3)<br>|__|
|__|__| MONTHS<br><br>NEVER BREAST<br>FED......................00<br>DON'T KNOW 98	|__|__| MONTHS<br><br>NEVER BREAST<br>FED...................... 00<br>DON'T KNOW 98	|__|__| MONTHS<br><br>NEVER BREAST<br>FED......................00<br>DON'T KNOW 98	|__|__| MONTHS<br><br>NEVER BREAST<br>FED...................... 00<br>DON'T KNOW 98	|__|__| MONTHS<br><br>NEVER BREAST<br>FED......................00<br>DON'T KNOW 98

BOX H-2

IF ADDITIONAL SPACE IS NEEDED, CHECK BOX ☐
AND GO TO SUPPLEMENT SHEET 1.

BOX H-3

CHECK H3 FOR ANY LIVE BIRTH. IF ANY LIVE BIRTH, ASK H5.
OTHERWISE, CHECK HERE ☐ AND SKIP TO H6.

H5. How old (were you/was _____) when (you/_____) first had a live birth?

|__|__|
AGE
DON'T KNOW .... 98 |__|__|

Now I have some questions about difficulties in becoming pregnant and maintaining pregnancies.

H6. (Have you/Did _____) ever visit(ed) a doctor, clinic, or hospital because of difficulty becoming pregnant?

YES 1
NO .... 2 } (H14)
DON'T KNOW .... 8 } (H14) |__|

H7. At what age did (you/_____) first visit a doctor, clinic, or hospital because of difficulty becoming pregnant?

|__|__|
AGE
DON'T KNOW .... 98 |__|__|

H8. Was any doctor able to tell (you/_____) why (you were/she was) having difficulty?

YES 1
NO .... 2 } (H10)
DON'T KNOW .... 8 } (H10) |__|

H9. What was the nature of the problem? (CIRCLE ALL THAT APPLY.)

PARTNER INFERTILE/SUBFERTILE ...... 01 |__|__|
CERVICAL FACTOR .... 02 |__|__|
TUBAL FACTOR .... 03 |__|__|
OVULATION FACTOR .... 04 |__|__|
HORMONAL PROBLEM .... 05 |__|__|
ENDOMETRIOSIS .... 06 |__|__|
OTHER (SPECIFY) .... 96 |__|__|
____
DON'T KNOW .... 98 |__|__|

H10. Did (you/_____) ever take any medications or hormones to help (you/her) become pregnant?

YES 1
NO .... 2 } (H14)
DON'T KNOW .... 8 } (H14) |__|

ASK H12 THROUGH H13 FOR EACH MEDICATION REPORTED IN H11.	H11. What is the name of the (first/next) medication or hormone (you/_____) took?	H12. At what age did (you/_____) start taking (MEDICATION)?	H13. For how many weeks, months, or years, in total, did (you/_____) take (MEDICATION)?
1ST MEDICATION	__________________<br>DON'T KNOW........ 98	|__|__| AGE<br>DON'T KNOW 98	|___|___| #<br>WKS.................. 1<br>MOS .... 2<br>YRS .... 3<br>DK .... 8 |__|
2ND MEDICATION	__________________<br>DON'T KNOW........ 98	|__|__| AGE<br>DON'T KNOW 98	|___|___| #<br>WKS.................. 1<br>MOS .... 2<br>YRS .... 3<br>DK .... 8 |__|
3RD MEDICATION	__________________<br>DON'T KNOW........ 98	|__|__| AGE<br>DON'T KNOW 98	|___|___| #<br>WKS.................. 1<br>MOS .... 2<br>YRS .... 3<br>DK .... 8 |__|

H14. (Have you/Did _____) ever visit(ed) a doctor, clinic, or hospital because of difficulty maintaining a pregnancy?

YES 1
NO .... 2 } (BOX H-4)
DON'T KNOW .... 8 } (BOX H-4) |__|

H15. At what age did (you/_____) first visit a doctor, clinic, or hospital because of difficulty maintaining a pregnancy?

|__|__|
AGE
DON'T KNOW .... 98 |__|__|

H16. Was any doctor able to tell (you/_____) why (you were/she was) having difficulty?

YES 1
NO .... 2 } (H18)
DON'T KNOW .... 8 } (H18) |__|

H17. What was the nature of the problem? (CIRCLE ALL THAT APPLY.)

CERVICAL FACTOR	1	|__|
TUBAL FACTOR	2	|__|
HORMONAL PROBLEM	3	|__|
ENDOMETRIOSIS	4	|__|
INFECTION	5	|__|
RUPTURE OF THE MEMBRANES	6	|__|
OTHER (SPECIFY)	7	|__|
___________________________________		
DON'T KNOW	8	|__|

H18. Did (you/_____) ever take any medications or hormones to help (you/her) maintain a pregnancy?

YES	1		
NO	2	} (BOX H-4)	|__|
DON'T KNOW	8		

ASK H20 THROUGH H21 FOR EACH MEDICATION REPORTED IN H19.	H19. What is the name of the (first/next) medication or hormone (you/_____) took?	H20. At what age did (you/_____) start taking (MEDICATION)?	H21. For how many weeks, months, or years, in total, did (you/_____) take (MEDICATION)?
1ST MEDICATION	NAME: ________________ DON'T KNOW 98	|__|__| AGE DON'T KNOW 98	|___|___| # WKS .... 1 MOS .... 2 YRS .... 3 DK .... 8 |__|
2ND MEDICATION	NAME: ________________ DON'T KNOW 98	|__|__| AGE DON'T KNOW 98	|___|___| # WKS .... 1 MOS .... 2 YRS .... 3 DK .... 8 |__|
3RD MEDICATION	NAME: ________________ DON'T KNOW 98	|__|__| AGE DON'T KNOW 98	|___|___| # WKS .... 1 MOS .... 2 YRS .... 3 DK .... 8 |__|

BOX H-4

CHECK H1, PAGE H-1.

IF NEVER PREGNANT OR DON'T KNOW (H1=2 OR 8) GO TO H24.
OTHERWISE ASK H22.

H22. Did (you/_____) ever take any medication to suppress lactation (that is, dry up milk supply)?

YES 1
NO .... 2 } (H24)
DON'T KNOW .... 8 } |__|

H23. What medication did (you/_____) take?

NAME: ____________________________

DON'T KNOW .... 8 |__|

H24. Did a doctor ever tell (you/________) that (you/she) had endometriosis?

YES.. 1
NO .... 2 } (SECTION J)
DON'T KNOW.. 8 } |__|

H25. How old (were you/ was ____) when the doctor first told (you/her) that (you/she) had endometriosis?

|__|__|
AGE
DON'T KNOW .... 98 |__|__|

H26. For endometriosis (have you/has__________) ever (TREATMENT)...

	YES	NO	DK	
Been hospitalized?......................................	1	2	8	|__|
Had surgery?...	1	2	8	|__|
Been prescribed medication?........................	1	2	8	|__|
(SPECIFY): ________________________				

SECTION J. HORMONE MEDICATION HISTORY

BOX J-1
**COMPLETE SECTION J FOR FEMALE SUBJECTS ONLY, SKIP TO SECTION K FOR MALE SUBJECTS.**

Now I have some questions about the use of female hormone medications.

J1. Did (you/_____) ever take any type of estrogen for relief of menopausal symptoms, irregular periods, or prevention of disease such as bone loss?

YES	1		
NO	2	} (SEC. L)	|__|
DON'T KNOW	8	} (SEC. L)	

J2. Were these estrogens in the form of a ... (READ EACH CATEGORY ONE AT A TIME.)

		YES	NO	DK	
a.	Pill?	1	2	8	|__|
b.	Shot?	1	2	8	|__|
c.	Hormonal vaginal cream or suppository?	1	2	8	|__|
d.	Patch or implant?	1	2	8	|__|
e.	Ring?	1	2	8	|__|
f.	Other form? (SPECIFY) _________________________________	1	2	8	|__|

BOX J-2
CHECK J2a.<br>IF ESTROGEN PILLS REPORTED, ASK J3-J8.<br>OTHERWISE, SKIP TO BOX J-4.

ESTROGEN PILLS

ASK J3-J8 FOR EACH PILL USE REPORTED.	J3.<br>At what age did (you/_____) (first/next) start taking estrogen pills?	J4.<br>What is the name of the (first/next) estrogen pill (you/_____) took? (ENTER COMPLETE NAME AND CODE. PROBE IF NECESSARY)	J5.<br>Why did (you/_____) take (PILL NAME)? Was it for:
1ST PILL USE	|__|__| AGE<br>DK 98	NAME: ________________<br>DON'T KNOW .... 98<br>|__|__|	Relief of menopausal symptoms, ...1<br>Irregular periods, ...2<br>Prevention of disease<br>such as bone loss, or ....3<br>Other reason? ....6<br>SPECIFY: _______________________<br>DON'T KNOW ....8 |__|
2ND PILL USE	|__|__| AGE<br>DK 98	NAME: ________________<br>DON'T KNOW .... 98<br>|__|__|	Relief of menopausal symptoms, ...1<br>Irregular periods, ...2<br>Prevention of disease<br>such as bone loss, or ....3<br>Other reason? ....6<br>SPECIFY: _______________________<br>DON'T KNOW ....8 |__|
3RD PILL USE	|__|__| AGE<br>DK 98	NAME: ________________<br>DON'T KNOW .... 98<br>|__|__|	Relief of menopausal symptoms, ...1<br>Irregular periods, ...2<br>Prevention of disease<br>such as bone loss, or ....3<br>Other reason? ....6<br>SPECIFY: _______________________<br>DON'T KNOW ....8 |__|
4TH PILL USE	|__|__| AGE<br>DK 98	NAME: ________________<br>DON'T KNOW .... 98<br>|__|__|	Relief of menopausal symptoms, ...1<br>Irregular periods, ...2<br>Prevention of disease<br>such as bone loss, or ....3<br>Other reason? ....6<br>SPECIFY: _______________________<br>DON'T KNOW ....8 |__|
5TH PILL USE	|__|__| AGE<br>DK 98	NAME: ________________<br>DON'T KNOW .... 98<br>|__|__|	Relief of menopausal symptoms, ...1<br>Irregular periods, ...2<br>Prevention of disease<br>such as bone loss, or ....3<br>Other reason? ....6<br>SPECIFY: _______________________<br>DON'T KNOW ....8 |__|

J6.<br>At what age did (you/_____) stop taking (PILL)?	J7.<br>While (you were/_____ was) taking (PILL), did (you/she) also take a progesterone pill?	J8.<br>What is the name of the progesterone pill that (you/_____) took with (PILL)? (ENTER COMPLETE NAME AND CODE. PROBE IF NECESSARY.)
|__|__| AGE<br><br>CURRENTLY TAKING .... 00<br>DK 98	YES .... 1<br>NO .... 2 } (NEXT PILL OR<br>DK .... 8 } BOX J-3) |__|	NAME: ___________________<br><br>DON'T KNOW .... 98 |__|__|
|__|__| AGE<br><br>CURRENTLY TAKING .... 00<br>DK 98	YES .... 1<br>NO .... 2 } (NEXT PILL OR<br>DK .... 8 } BOX J-3) |__|	NAME: ___________________<br><br>DON'T KNOW .... 98 |__|__|
|__|__| AGE<br><br>CURRENTLY TAKING .... 00<br>DK 98	YES .... 1<br>NO .... 2 } (NEXT PILL OR<br>DK .... 8 } BOX J-3) |__|	NAME: ___________________<br><br>DON'T KNOW .... 98 |__|__|
|__|__| AGE<br><br>CURRENTLY TAKING .... 00<br>DK 98	YES .... 1<br>NO .... 2 } (NEXT PILL OR<br>DK .... 8 } BOX J-3) |__|	NAME: ___________________<br><br>DON'T KNOW .... 98 |__|__|
|__|__| AGE<br><br>CURRENTLY TAKING .... 00<br>DK 98	YES .... 1<br>NO .... 2 } (NEXT PILL OR<br>DK .... 8 } BOX J-3) |__|	NAME: ___________________<br><br>DON'T KNOW .... 98 |__|__|

ESTROGEN SHOTS

BOX J-3

CHECK J2b.
IF ESTROGEN SHOTS REPORTED, ASK J9-J11.
OTHERWISE, SKIP TO SECTION L

J9. At what age did (you/_____) first start receiving estrogen shots?	J10. At what age did (you/_____) last receive estrogen shots?	J11. How many times per day, week, month, or year did (you/_____) receive estrogen shots between ages (AGES IN J9 AND J10)?
|__|__| AGE<br><br>DON'T KNOW 98	|__|__| AGE<br><br>CURRENTLY RECEIVING ...... 00<br>DON'T KNOW 98	|___|___| TIMES PER<br>DAY .... 1<br>WEEK .... 2<br>MONTH .... 3<br>YEAR .... 4<br>DK .... 988 |__|

SKIP TO SECTION L FOR ALL FEMALE SUBJECTS.

SECTION K.   MALE FERTILITY

**BOX K-1**
**COMPLETE SECTION K FOR MALE SUBJECTS ONLY,** **SKIP TO SECTION L FOR FEMALE SUBJECTS.**

Now I have some questions about (your/_____'s) fertility.

K1. How many live-born children (have you fathered/did _____ father)? Please exclude any children for whom you are not the biological father, such as stepchildren, foster children, or adopted children.

|__|__|
NUMBER

NONE 00
DONT KNOW .... 98 |__|__|

K2. Before one year ago, did (you/_____) ever try for a year or longer to have a child without success?

YES 1
NO .... 2 } (K5)
DON'T KNOW .... 8 } (K5) |__|

K3. Did (you/_____) or (your/_____'s) wife or partner seek medical advice for this problem?

YES 1
NO .... 2 } (K5)
DON'T KNOW .... 8 } (K5) |__|

K4. What was the main reason for this problem?

PARTNER INFERTILE/SUBFERTILE	1		
SUBJECT INFERTILE/SUBFERTILE (LOW/ABNORMAL SPERM COUNT)	2		
SUBJECT AND PARTNER INFERTILE/SUBFERTILE	3		
OTHER (SPECIFY) ____	6		
DON'T KNOW	8		|__|

K5. (Have you/Had ______) had a vasectomy, that is, a sterilization operation for men?

YES	1		
NO	2	} (SEC. L)	|__|
DON'T KNOW	8	} (SEC. L)	

K6. How old (were you/was _______) when (you/he) had a vasectomy?

|__|__|
AGE

DON'T KNOW	98	|__|__|

SECTION L.   FAMILY HISTORY OF DISEASE

**BOX L-1**
CHECK A7 MARITAL STATUS. IF A7 = 1, 3, 4, OR 5 (EVER MARRIED), ASK L1.<br>OTHERWISE, CHECK BOX ☐ AND SKIP TO L4.

Now I have a few questions about (your/_________'s) spouse.

L1. In what year was (your/_____'s) spouse born?

19|__|__|
YEAR
DON'T KNOW .... 98 |__|__|

L2. Is (he/she) alive now?

YES... 1 (L4)
NO 2
DON'T KNOW .... 8 (L4) |__|

L3. At what age did (he/she) die?

|__|__|
AGE
DON'T KNOW .... 98 |__|__|

Now I have a few questions about some of (your/_________'s) blood relatives. I am only interested in (your/his/her) relatives who are related by blood. Do not include relatives not related by blood, such as adopted or foster relatives. I will be asking about (your/his/her) mother, father, sisters, brothers, and children.

L4. Can you answer questions about the health of (your/______'s) biological parents, or brothers or sisters with whom (you have/_____ has) at least one biological parent in common?

YES... 1
NO .... 2 (SECTION M) |__|

L5. (Were you/Was ______) born a twin (or triplet)?

YES... 1
NO .... 2 } (L7) |__|
DON'T KNOW .... 8 }

L6. (Were you/Was ______) born an identical or fraternal twin (or triplet)?
[Translation note: Identical twins (or triplets) develop from a single fertilized egg. Fraternal twins (or triplets) develop from separate fertilized eggs.]

IDENTICAL 1
FRATERNAL 2
DON'T KNOW .... 8 |__|

L7. In what year was (your/______'s) mother born?

|__|__|__|__|
YEAR
DON'T KNOW .... 9998 |__|__|__|__|

L8. Is she alive now?

YES.. 1 (L10)
NO 2
DON'T KNOW .... 8 (L10) |__|

L9. At what age did she die?

|__|__|__|
AGE
DON'T KNOW .... 998 |__|__|__|

L10. In what year was (your/______'s) father born?

|__|__|__|__|
YEAR
DON'T KNOW .... 9998 |__|__|__|__|

L11. Is he alive now?

YES.. 1 (L13)
NO 2
DON'T KNOW .... 8 (L13) |__|

L12. At what age did he die?

|__|__|__|
AGE
DON'T KNOW .... 998 |__|__|__|

L13. How many (RELATIONSHIP) (do you/does _______) have, both living and deceased?

a. Full sisters |__|__|
#
NONE .... 00 (b)
DON'T KNOW .... 98 (b) |__|__|

b. Full brothers |__|__|
#
NONE .... 00 (c)
DON'T KNOW .... 98 (c) |__|__|

c. Half sisters |__|__|
#
NONE .... 00 (d)
DON'T KNOW .... 98 (d) |__|__|

[Translation note: Sisters with whom (you share/_____ shares) a biological mother or a biological father.]

d. Half brothers |__|__|
#
NONE .... 00 (e)
DON'T KNOW .... 98 (e) |__|__|

[Translation note: Brothers with whom (you share/_____ shares) a biological mother or a biological father.]

e. Daughters |__|__|
#
NONE .... 00 (f)
DON'T KNOW .... 98 (f) |__|__|

f. Sons |__|__|
#
NONE .... 00 (BOX L-2)
DON'T KNOW .... 98 (BOX L-2) |__|__|

BOX L-2

FOR EACH RELATIONSHIP WITH A NUMBER ENTERED IN THE BOX, GO TO L14, ENTER THE NAMES, CIRCLE THE RELATIONSHIP CODES AND ASK L15 THROUGH L18, AS APPROPRIATE.

L14.<br><br>Could I please have the name(s) of (your/his/her) (RELATIONSHIP)?	L15.<br>Which parent (do you/does _____) share with (NAME)?	L16.<br><br>In what year was (NAME) born?	L17.<br><br><br>Is (he/she) alive now?	L18.<br><br>At what age did (he/she) die?
a. NAME:<br>_______________<br>FULL SISTER .... 1 (L16)<br>FULL BROTHER..... 2 (L16)<br>HALF SISTER.......... 3<br>HALF BROTHER .... 4<br>DAUGHTER .... 5 (L16)<br>SON 6 (L16)	MOTHER....... 1<br><br>FATHER .... 2<br><br>DK.................. 8<br><br>|__|	19 |__|__| YEAR<br><br>DK.........98	YES.......1 (b)<br>NO ....2<br>DK ....8 (b) |__|	|__|__|__| AGE<br>DK........... 998
b. NAME:<br>_______________<br>FULL SISTER .... 1 (L16)<br>FULL BROTHER..... 2 (L16)<br>HALF SISTER.......... 3<br>HALF BROTHER .... 4<br>DAUGHTER .... 5 (L16)<br>SON 6 (L16)	MOTHER....... 1<br><br>FATHER .... 2<br><br>DK.................. 8<br><br>|__|	19 |__|__| YEAR<br><br>DK.........98	YES.......1 (c)<br>NO ....2<br>DK ....8 (c) |__|	|__|__|__| AGE<br>DK........... 998
c. NAME:<br>_______________<br>FULL SISTER .... 1 (L16)<br>FULL BROTHER..... 2 (L16)<br>HALF SISTER.......... 3<br>HALF BROTHER .... 4<br>DAUGHTER .... 5 (L16)<br>SON 6 (L16)	MOTHER....... 1<br><br>FATHER .... 2<br><br>DK.................. 8<br><br>|__|	19 |__|__| YEAR<br><br>DK.........98	YES.......1 (d)<br>NO ....2<br>DK ....8 (d) |__|	|__|__|__| AGE<br>DK........... 998
d. NAME:<br>_______________<br>FULL SISTER .... 1 (L16)<br>FULL BROTHER..... 2 (L16)<br>HALF SISTER.......... 3<br>HALF BROTHER .... 4<br>DAUGHTER .... 5 (L16)<br>SON 6 (L16)	MOTHER....... 1<br><br>FATHER .... 2<br><br>DK.................. 8<br><br>|__|	19 |__|__| YEAR<br><br>DK.........98	YES.......1 (e)<br>NO ....2<br>DK ....8 (e) |__|	|__|__|__| AGE<br>DK........... 998
e. NAME:<br>_______________<br>FULL SISTER .... 1 (L16)<br>FULL BROTHER..... 2 (L16)<br>HALF SISTER.......... 3<br>HALF BROTHER .... 4<br>DAUGHTER .... 5 (L16)<br>SON 6 (L16)	MOTHER....... 1<br><br>FATHER .... 2<br><br>DK.................. 8<br><br>|__|	19 |__|__| YEAR<br><br>DK.........98	YES.......1 (f)<br>NO ....2<br>DK ....8 (f) |__|	|__|__|__| AGE<br>DK........... 998

L14.<br><br>Could I please have the name(s) of (your/his/her) (RELATIONSHIP)?	L15.<br>Which parent (do you/does _____) share with (NAME)?	L16.<br><br>In what year was (NAME) born?	L17.<br><br>Is (he/she) alive now?	L18.<br><br>At what age did (he/she) die?
f. NAME: _______________<br>FULL SISTER .... 1 (L16)<br>FULL BROTHER..... 2 (L16)<br>HALF SISTER.......... 3<br>HALF BROTHER .... 4<br>DAUGHTER .... 5 (L16)<br>SON 6 (L16)	MOTHER.......1<br>FATHER ....2<br>DK..................8<br>|__|	19 |__|__| YEAR<br>DK............ 98	YES.......1 (g)<br>NO ....2<br>DK ....8 (g) |__|	|__|__|__| AGE<br>DK...............998
g. NAME: _______________<br>FULL SISTER .... 1 (L16)<br>FULL BROTHER..... 2 (L16)<br>HALF SISTER.......... 3<br>HALF BROTHER .... 4<br>DAUGHTER .... 5 (L16)<br>SON 6 (L16)	MOTHER.......1<br>FATHER ....2<br>DK..................8<br>|__|	19 |__|__| YEAR<br>DK............ 98	YES.......1 (h)<br>NO ....2<br>DK ....8 (h) |__|	|__|__|__| AGE<br>DK...............998
h. NAME: _______________<br>FULL SISTER .... 1 (L16)<br>FULL BROTHER..... 2 (L16)<br>HALF SISTER.......... 3<br>HALF BROTHER .... 4<br>DAUGHTER .... 5 (L16)<br>SON 6 (L16)	MOTHER.......1<br>FATHER ....2<br>DK..................8<br>|__|	19 |__|__| YEAR<br>DK............ 98	YES.......1 (i)<br>NO ....2<br>DK ....8 (i) |__|	|__|__|__| AGE<br>DK...............998
i. NAME: _______________<br>FULL SISTER .... 1 (L16)<br>FULL BROTHER..... 2 (L16)<br>HALF SISTER.......... 3<br>HALF BROTHER .... 4<br>DAUGHTER .... 5 (L16)<br>SON 6 (L16)	MOTHER.......1<br>FATHER ....2<br>DK..................8<br>|__|	19 |__|__| YEAR<br>DK............ 98	YES.......1 (j)<br>NO ....2<br>DK ....8 (j) |__|	|__|__|__| AGE<br>DK...............998
j. NAME: _______________<br>FULL SISTER .... 1 (L16)<br>FULL BROTHER..... 2 (L16)<br>HALF SISTER.......... 3<br>HALF BROTHER .... 4<br>DAUGHTER .... 5 (L16)<br>SON 6 (L16)	MOTHER.......1<br>FATHER ....2<br>DK..................8<br>|__|	19 |__|__| YEAR<br>DK............ 98	YES.......1 (k)<br>NO ....2<br>DK ....8 (k) |__|	|__|__|__| AGE<br>DK...............998

L14.<br><br>Could I please have the name(s) of (your/his/her) (RELATIONSHIP)?	L15.<br>Which parent (do you/does _____) share with (NAME)?	L16.<br><br>In what year was (NAME) born?	L17.<br><br><br>Is (he/she) alive now?	L18.<br><br>At what age did (he/she) die?
k. NAME: ______________<br>FULL SISTER .... 1 (L16)<br>FULL BROTHER..... 2 (L16)<br>HALF SISTER.......... 3<br>HALF BROTHER .... 4<br>DAUGHTER .... 5 (L16)<br>SON 6 (L16)	MOTHER....... 1<br><br>FATHER........ 2<br><br>DK.................. 8<br><br>|__|	19 |__|__| YEAR<br><br>DK............ 98	YES.......1 (l)<br>NO ....2<br>DK ....8 (l) |__|	|__|__|__| AGE<br>DK...............998
l. NAME: ______________<br>FULL SISTER .... 1 (L16)<br>FULL BROTHER..... 2 (L16)<br>HALF SISTER.......... 3<br>HALF BROTHER .... 4<br>DAUGHTER .... 5 (L16)<br>SON 6 (L16)	MOTHER....... 1<br><br>FATHER........ 2<br><br>DK.................. 8<br><br>|__|	19 |__|__| YEAR<br><br>DK............ 98	YES.......1 (m)<br>NO ....2<br>DK ....8 (m) |__|	|__|__|__| AGE<br>DK...............998
m. NAME: ______________<br>FULL SISTER .... 1 (L16)<br>FULL BROTHER..... 2 (L16)<br>HALF SISTER.......... 3<br>HALF BROTHER .... 4<br>DAUGHTER .... 5 (L16)<br>SON 6 (L16)	MOTHER....... 1<br><br>FATHER........ 2<br><br>DK.................. 8<br><br>|__|	19 |__|__| YEAR<br><br>DK............ 98	YES.......1 (n)<br>NO ....2<br>DK ....8 (n) |__|	|__|__|__| AGE<br>DK...............998
n. NAME: ______________<br>FULL SISTER .... 1 (L16)<br>FULL BROTHER..... 2 (L16)<br>HALF SISTER.......... 3<br>HALF BROTHER .... 4<br>DAUGHTER .... 5 (L16)<br>SON 6 (L16)	MOTHER....... 1<br><br>FATHER........ 2<br><br>DK.................. 8<br><br>|__|	19 |__|__| YEAR<br><br>DK............ 98	YES.......1 (o)<br>NO ....2<br>DK ....8 (o) |__|	|__|__|__| AGE<br>DK...............998
o. NAME: ______________<br>FULL SISTER .... 1 (L16)<br>FULL BROTHER..... 2 (L16)<br>HALF SISTER.......... 3<br>HALF BROTHER .... 4<br>DAUGHTER .... 5 (L16)<br>SON 6 (L16)	MOTHER....... 1<br><br>FATHER........ 2<br><br>DK.................. 8<br><br>|__|	19 |__|__| YEAR<br><br>DK............ 98	YES.......1 (p)<br>NO ....2<br>DK ....8 (p) |__|	|__|__|__| AGE<br>DK...............998

L14.<br><br>Could I please have the name(s) of (your/his/her) (RELATIONSHIP)?	L15.<br>Which parent (do you/does _____) share with (NAME)?	L16.<br><br>In what year was (NAME) born?	L17.<br><br><br>Is (he/she) alive now?	L18.<br><br>At what age did (he/she) die?
p. NAME: ______________<br>FULL SISTER .... 1 (L16)<br>FULL BROTHER..... 2 (L16)<br>HALF SISTER.......... 3<br>HALF BROTHER .... 4<br>DAUGHTER .... 5 (L16)<br>SON 6 (L16)	MOTHER....... 1<br><br>FATHER .... 2<br><br>DK.................. 8<br><br>|__|	19 |__|__| YEAR<br><br>DK............ 98	YES.......1 (q)<br>NO ....2<br>DK ....8 (q) |__|	|__|__|__| AGE<br>DK...............998
q. NAME: ______________<br>FULL SISTER .... 1 (L16)<br>FULL BROTHER..... 2 (L16)<br>HALF SISTER.......... 3<br>HALF BROTHER .... 4<br>DAUGHTER .... 5 (L16)<br>SON 6 (L16)	MOTHER....... 1<br><br>FATHER .... 2<br><br>DK.................. 8<br><br>|__|	19 |__|__| YEAR<br><br>DK............ 98	YES.......1 (r)<br>NO ....2<br>DK ....8 (r) |__|	|__|__|__| AGE<br>DK...............998
r. NAME: ______________<br>FULL SISTER .... 1 (L16)<br>FULL BROTHER..... 2 (L16)<br>HALF SISTER.......... 3<br>HALF BROTHER .... 4<br>DAUGHTER .... 5 (L16)<br>SON 6 (L16)	MOTHER....... 1<br><br>FATHER .... 2<br><br>DK.................. 8<br><br>|__|	19 |__|__| YEAR<br><br>DK............ 98	YES.......1 (s)<br>NO ....2<br>DK ....8 (s) |__|	|__|__|__| AGE<br>DK...............998
s. NAME: ______________<br>FULL SISTER .... 1 (L16)<br>FULL BROTHER..... 2 (L16)<br>HALF SISTER.......... 3<br>HALF BROTHER .... 4<br>DAUGHTER .... 5 (L16)<br>SON 6 (L16)	MOTHER....... 1<br><br>FATHER .... 2<br><br>DK.................. 8<br><br>|__|	19 |__|__| YEAR<br><br>DK............ 98	YES.......1 (t)<br>NO ....2<br>DK ....8 (t) |__|	|__|__|__| AGE<br>DK...............998
t. NAME: ______________<br>FULL SISTER .... 1 (L16)<br>FULL BROTHER..... 2 (L16)<br>HALF SISTER.......... 3<br>HALF BROTHER .... 4<br>DAUGHTER .... 5 (L16)<br>SON 6 (L16)	MOTHER....... 1<br><br>FATHER .... 2<br><br>DK.................. 8<br><br>|__|	19 |__|__| YEAR<br><br>DK............ 98	YES... 1 (K19 INTRO)<br>NO .... 2<br>DK .... 8 (K19 INTRO)<br>|__|	|__|__|__| AGE<br>DK...............998

BOX L-3

ASK L19 ABOUT SUBJECT'S MOTHER, FATHER, SPOUSE, SIBLINGS, AND CHILDREN. FOR EACH "YES", GO TO THE APPROPRIATE GRID FOR a, b, c, d, e, f, or g AND ENTER THE NAMES AND RELATIONSHIPS FOR ALL OF THE BLOOD RELATIVES OR SPOUSE WHO HAD THAT CONDITION. THEN, FOR EACH RELATIVE, ASK THE OTHER QUESTIONS IN THE GRID.

SHOW CARD L-1

Now, I'm going to ask you about the list of conditions shown on this card. For each condition, I will ask whether (any of the relatives you have just told me about, including) (your/_____'s) mother and father (and spouse) were ever told by a doctor that he or she had that condition.

L19. Were any of these relatives ever told by a doctor that they had (CONDITION)?

		YES	NO	DK	
a.	Gallbladder cancer?..	1 (L20)	2	8	|__|
b.	Extrahepatic bile duct cancer?	1 (L24)	2	8	|__|
c.	Liver cancer?..	1 (L27)	2	8	|__|
d.	Any other cancer?	1 (L30)	2	8	|__|
e.	Gallstones?..	1 (L34)	2	8	|__|
f.	Hepatitis?	1 (L38)	2	8	|__|
g.	Diabetes?...	1 (L42)	2	8	|__|

A. GALLBLADDER CANCER

L20.<br>Which relatives had gallbladder cancer?<br>(ENTER FIRST NAME AND RELATIONSHIP FOR ALL WHO HAD CONDITION)	L21.<br>How old was (NAME) when it was diagnosed?	L22.<br>Did (NAME) have a cholecystectomy?	L23.<br>What is the name and province of the hospital where it was diagnosed?
a.<br>Name: ____________________________<br>Relationship: ________________________<br>|___|___|	|__|__|__| AGE<br>DON'T KNOW............. 998	YES.........1<br>NO...........2<br>DK...........8 |__|	______________________<br>______________________
b.<br>Name: ____________________________<br>Relationship: ________________________<br>|___|___|	|__|__|__| AGE<br>DON'T KNOW............. 998	YES.........1<br>NO...........2<br>DK...........8 |__|	______________________<br>______________________

RETURN TO L19

B. EXTRAHEPATIC BILE DUCT CANCER

L24.<br>Which relative had bile duct cancer?<br>(ENTER FIRST NAME AND RELATIONSHIP FOR ALL WHO HAD CONDITION)	L25.<br>How old was (NAME) when it was diagnosed?	L26.<br>What is the name and province of the hospital where it was diagnosed?
a.<br>Name: ____________________________<br>Relationship: ________________________<br>|___|___|	|__|__|__| AGE<br>DON'T KNOW............. 998	____________________________<br>____________________________
b.<br>Name: ____________________________<br>Relationship: ________________________<br>|___|___|	|__|__|__| AGE<br>DON'T KNOW............. 998	____________________________<br>____________________________

RETURN TO L19

C. LIVER CANCER

L27. Which relative had liver cancer? (ENTER FIRST NAME AND RELATIONSHIP FOR ALL WHO HAD CONDITION)	L28. How old was (NAME) when it was diagnosed?	L29. What is the name and province of the hospital where it was diagnosed?
a. Name: ____________ Relationship: ____________ |___|___|	|__|__|__| AGE DON'T KNOW............. 998	____________ ____________
b. Name: ____________ Relationship: ____________ |___|___|	|__|__|__| AGE DON'T KNOW............. 998	____________ ____________

RETURN TO L19

D. OTHER CANCER

L30. Which relative had another cancer? (ENTER FIRST NAME AND RELATIONSHIP FOR ALL WHO HAD CONDITION)	L31. What type of cancer did (NAME) have? Where did the cancer start?	L32. How old was (he/she) when it was diagnosed?	L33. What is the name and province of the hospital where it was diagnosed?
a. Name: ____________ Relationship: ____________ |___|___|	SITE/TYPE: ____________ ____________ |__|__|__|__|__|	|__|__|__| AGE DK............... 998	____________ ____________
b. Name: ____________ Relationship: ____________ |___|___|	SITE/TYPE: ____________ ____________ |__|__|__|__|__|	|__|__|__| AGE DK............... 998	____________ ____________
c. Name: ____________	SITE/TYPE: ____________	|__|__|__| AGE	____________

Relationship: ______________________<br>|___|___|	____________<br>|__|__|__|__|__|	DK................ 998	____________________________

RETURN TO L19

E. GALLSTONES

L34.<br>Which relative had gallstones? (ENTER FIRST NAME AND RELATIONSHIP FOR ALL WHO HAD CONDITION)	L35.<br>How old was (NAME) when it was diagnosed?	L36.<br>How was it treated? (CIRCLE ALL THAT APPLY)	L37.<br>What is the name and province of the hospital where it was diagnosed?
a.<br>Name: ____________________<br>Relationship: _______________<br>|___|___|	|__|__|__| AGE<br>DK 998	REMOVAL OF THE GALLBLADDER ....1 |__| MEDICATIONS ....2 |__| A SPECIFIC DIET..................3 |__| OTHER (SPECIFY) ....<br>..6 |__| DON'T KNOW........................8 |__|	____________________<br>____________________<br>____________________
b.<br>Name: ____________________<br>Relationship: _______________<br>|___|___|	|__|__|__| AGE<br>DK 998	REMOVAL OF THE GALLBLADDER ....1 |__| MEDICATIONS ....2 |__| A SPECIFIC DIET..................3 |__| OTHER (SPECIFY) ....<br>..6 |__| DON'T KNOW........................8 |__|	____________________<br>____________________<br>____________________
c.<br>Name: ____________________<br>Relationship: _______________<br>|___|___|	|__|__|__| AGE<br>DK 998	REMOVAL OF THE GALLBLADDER ....1 |__| MEDICATIONS ....2 |__| A SPECIFIC DIET..................3 |__| OTHER (SPECIFY) ....<br>..6 |__| DON'T KNOW........................8 |__|	____________________<br>____________________<br>____________________

RETURN TO L19

F. HEPATITIS

L38.<br>Which relative had hepatitis?<br>(ENTER FIRST NAME AND RELATIONSHIP FOR ALL WHO HAD CONDITION)	L39.<br>How old was (NAME) when it was diagnosed?	L40.<br>What type was it?<br>(CIRCLE ALL THAT APPLY)	L41.<br>What is the name and province of the hospital where it was diagnosed?
a.<br>Name: ____________________<br>Relationship: _______________<br>|___|___|	|__|__|__| AGE<br>DK 998	HEPATITIS A.........................1 |__| HEPATITIS B.........................2 |__| HEPATITIS C.........................3 |__| ACUTE..................................4 |__| CHRONIC..............................5 |__| DON'T KNOW.......................8 |__|	____________________<br>____________________<br>____________________
b.<br>Name: ____________________<br>Relationship: _______________<br>|___|___|	|__|__|__| AGE<br>DK 998	HEPATITIS A.........................1 |__| HEPATITIS B.........................2 |__| HEPATITIS C.........................3 |__| ACUTE..................................4 |__| CHRONIC..............................5 |__| DON'T KNOW.......................8 |__|	____________________<br>____________________<br>____________________
c.<br>Name: ____________________<br>Relationship: _______________<br>|___|___|	|__|__|__| AGE<br>DK 998	HEPATITIS A.........................1 |__| HEPATITIS B.........................2 |__| HEPATITIS C.........................3 |__| ACUTE..................................4 |__| CHRONIC..............................5 |__| DON'T KNOW.......................8 |__|	____________________<br>____________________<br>____________________
d.<br>Name: ____________________<br>Relationship: _______________<br>|___|___|	|__|__|__| AGE<br>DK 998	HEPATITIS A.........................1 |__| HEPATITIS B.........................2 |__| HEPATITIS C.........................3 |__| ACUTE..................................4 |__| CHRONIC..............................5 |__| DON'T KNOW.......................8 |__|	____________________<br>____________________<br>____________________

RETURN TO L19

G. DIABETES

L42. Which relative had diabetes? (ENTER FIRST NAME AND RELATIONSHIP FOR ALL WHO HAD CONDITION)	L43. How old was (NAME) when it was diagnosed?	L44. What type was it?	L45. What is the name and province of the hospital where it was diagnosed?
a. Name: ____________________ Relationship: _______________ |___|___|	|__|__|__| AGE DK 998	INSULIN DEPENDENT (TYPE I)...1 INSULIN INDEPENDENT (TYPE II) ....2 PREGNANCY OR GESTATIONAL3 DON'T KNOW ....8 |__|	____________________ ____________________
b. Name: ____________________ Relationship: _______________ |___|___|	|__|__|__| AGE DK 998	INSULIN DEPENDENT (TYPE I)...1 INSULIN INDEPENDENT (TYPE II) ....2 PREGNANCY OR GESTATIONAL3 DON'T KNOW ....8 |__|	____________________ ____________________
c. Name: ____________________ Relationship: _______________ |___|___|	|__|__|__| AGE DK 998	INSULIN DEPENDENT (TYPE I)...1 INSULIN INDEPENDENT (TYPE II) ....2 PREGNANCY OR GESTATIONAL3 DON'T KNOW ....8 |__|	____________________ ____________________

L46. Are there any illnesses or conditions besides those just discussed that tend to run in (your/_____'s) family?

YES.. 1
NO .... 2 } (BOX L4) |__|
DON'T KNOW .... 8 }

L47. What illnesses or conditions, besides those we have discussed, tend to run in (your/_____'s) family?

a. ____

b. ____

c. ____

d. ____

e. ____

BOX L-4

INTERVIEWER: WHO PROVIDED <u>MOST</u> OF THE RESPONSES IN THIS SECTION?

SUBJECT 1

PROXY... 2
BOTH EQUALLY.......................... 3 |__|

SECTION M. PHYSICAL DEVELOPMENT AND ACTIVITY

Now I will ask you some questions about (your/_____'s) physical development.

M1. As an adult, what (is your/was ______'s) height in centimeters?

|__|__|__| cm
DON'T KNOW .... 998 |__|__|__|

M2. When (you were/______ was) (AGE CATEGORY), how did (your/his/her) height compare with other (girls/boys) (your/his/her) age? (Were you/was he/she) much shorter, somewhat shorter, about the same, somewhat taller, or much taller?

	A. MUCH SHORTER	B. SOMEWHAT SHORTER	C. ABOUT THE SAME	D. SOMEWHAT TALLER	E. MUCH TALLER	F. DON'T KNOW	
a. 9 or 10 (in the 4th or 5th grade)	1	2	3	4	5	8	|__|
b. 12 or 13 (in junior middle school)	1	2	3	4	5	8	|__|
c. 15 or 16 (in senior middle school)	1	2	3	4	5	8	|__|

M3. As an adult, what (is your/was______'s) usual weight in kilograms?

|__|__|__| kg
DON'T KNOW .... 998 |__|__|__|

M4. What (is your/was _____'s) current weight?

|__|__|__| kg
DON'T KNOW .... 998 |__|__|__|

M5. What was (your/_____'s) usual weight 5 years ago?

|__|__|__| kg
DON'T KNOW .... 998 |__|__|__|

ASK EACH AGE GROUP ENDING WITH CURRENT AGE GROUP. ALSO ASK 1990.

	8-9	12-14	20-29	30-39	40-49	50-59	60-69	1990
M6. Now I'd like you to think about (your/______'s) weight. [When (you were/he/she was) about ____,/In 1990] compared to others (your/his/her) age and height, (were you/was he/she):	|__|	|__|	|__|	|__|	|__|	|__|	|__|	|__|
Very thin, ...	1	1	1	1	1	1	1	1
Somewhat thin, ...	2	2	2	2	2	2	2	2
Average,...............................	3	3	3	3	3	3	3	3
Somewhat heavy, or.............	4	4	4	4	4	4	4	4
Very heavy?	5	5	5	5	5	5	5	5
DON'T KNOW	8	8	8	8	8	8	8	8
NOT APPLICABLE	+	+	+	+	+	+	+	+
M7. What was (your/______'s) weight in kilograms [at age ____ / in 1990]?		|__|__|__| KG	|__|__|__| KG	|__|__|__| KG	|__|__|__| KG	|__|__|__| KG	|__|__|__| KG	|__|__|__| KG
DON'T KNOW		998	998	998	998	998	998	998
M8. What was (your/______'s) trouser waist size in centimeters [at age ____ / in 1990]?		|__|__|__| CM	|__|__|__| CM	|__|__|__| CM	|__|__|__| CM	|__|__|__| CM	|__|__|__| CM	|__|__|__| CM
DON'T KNOW		998	998	998	998	998	998	998

M9. What was (your/______'s) highest adult weight? (IF FEMALE SUBJECT: Do not count any times you were pregnant, nursing, or during the six months after a pregnancy.)

|__|__|__|
KG
DON'T KNOW .... 998 |__|__|__|

M10. At what age did (you/______) first reach this weight?

|__|__|
AGE
DON'T KNOW .... 98 |__|__|

M11. For how many years or months (were you/was ______) at this weight?

|__|__| NUMBER
YEARS 1
MONTHS 2
DON'T KNOW .... 988 |__|__|__|

BOX M-1

IF PROXY INTERVIEW, CHECK HERE ☐ AND SKIP TO SECTION M.

M12. When you gain weight, where on your body do you mainly tend to add the weight?

DON'T GAIN WEIGHT	0
AROUND THE CHEST AND SHOULDERS	1
AROUND THE WAIST/STOMACH	2
AROUND THE HIPS AND THIGHS	3
EQUALLY ALL OVER	4
OTHER (SPECIFY) ______________	6
DON'T KNOW	8

|__|

M13. (IF FEMALE SUBJECT: Not counting after a pregnancy), how many times since you were age 20 have you lost as much as 7 kilograms or more and then later gained it back?

|__|__|
TIMES

NONE	00
DON'T KNOW	98

|__|__|

M14. Think about your physical activity before 5 years ago, ignoring any recent changes. How often did you participate in vigorous physical activities such as lap swimming, basketball, gymnastics, running, fast cycling, or aerobics?

SHOW CARD C

A.	DAILY	1
B.	4-6 TIMES PER WEEK	2
C.	2-3 TIMES PER WEEK	3
D.	ONCE PER WEEK	4
E.	1-3 TIMES PER MONTH	5
F.	LESS THAN ONCE PER MONTH OR NEVER	6
G.	DON'T KNOW	8

|__|

M15. Before 5 years ago, how often did you participate in moderate physical activities such as brisk walking, volleyball, dance, table tennis, softball, leisurely cycling, or tai chi?

SHOW CARD D

A.	DAILY	1
B.	4-6 TIMES PER WEEK	2
C.	2-3 TIMES PER WEEK	3
D.	ONCE PER WEEK	4
E.	1-3 TIMES PER MONTH	5
F.	LESS THAN ONCE PER MONTH OR NEVER	6
G.	DON'T KNOW	8

|__|

M16. Before 5 years ago, how often did you climb at least two or more flights of stairs without stopping? A flight should be at least 10 steps.

A.	DAILY	1
B.	4-6 TIMES PER WEEK	2
C.	2-3 TIMES PER WEEK	3
D.	ONCE PER WEEK	4
E.	1-3 TIMES PER MONTH	5
F.	LESS THAN ONCE PER MONTH OR NEVER	6
G.	DON'T KNOW	8

|__|

M17. Before 5 years ago, what method of travel did (you/______) usually use to go between home and work? Did (you/______) usually ...

Walk..	1	
Bicycle..	2	
Ride a moped, or..	3	
Use another method of travel?	6	(SECTION N)
(SPECIFY)___________________________		(SECTION N)
DON'T KNOW	8	(SECTION N)

|__|

REFER TO METHOD OF TRAVEL INDICATED IN M17 WHEN ASKING M18 AND M19.

M18. Before 5 years ago, when (you/______) (METHOD OF TRAVEL) between home and work, how many kilometers did (you/______) travel round-trip using this method of transportation?

|__|__| km

DON'T KNOW 98

|__|__|

M19. Before 5 years ago, when (you/______) (METHOD OF TRAVEL) between home and work, how many times per day, per week, or per month did (you/______) travel round-trip using this method of transportation?

|__|__|	PER DAY................................ 1		
NUMBER	PER WEEK............................. 2		
	PER MONTH.......................... 3		
	DON'T KNOW........................ 988		|__|__|__|

SECTION N. OCCUPATIONAL HISTORY

Next, I will ask about (your/_____'s) occupational history.

N1. (Have you/had______) ever had a job for six months or more? Please include full-time or part-time, paid or unpaid work, and also any periods of self-employment.

YES 1
NO .... 2 } (SECTION P) |__|
DON'T KNOW .... 8 }

I would like some information about the three different types of work that (you/______) did the longest. (ASK N2-N7 FOR THE THREE TYPES OF WORK DONE THE LONGEST.)

	N2. What was the job title for the type of work that (you/______) did the (longest/next longest)?	N3. What were (your/______'s) usual activities or duties as a (JOB TITLE)?	N4. In the place where (you/______) usually worked, what did that part of the company specialize in, that is, what did they make or do?
LONGEST OCCUPATION			
NEXT LONGEST OCCUPATION			
NEXT LONGEST OCCUPATION			

N5.<br>In what year did (you/______) start working as a (JOB TITLE)?	N6.<br>Altogether, how many years did (you/______) work at that type of job?	N7.<br>Would you say (your/______'s) usual physical activity on that job was light, moderately strenuous, or very strenuous?
19 |__|__| START YEAR<br>DON'T KNOW 98	|__|__| # YEARS<br>DON'T KNOW 98	LIGHT .... 1<br>MODERATELY STRENUOUS .... 2<br>VERY STRENUOUS................................ 3<br>DON'T KNOW .... 8 |__|
19 |__|__| START YEAR<br>DON'T KNOW 98	|__|__| # YEARS<br>DON'T KNOW 98	LIGHT .... 1<br>MODERATELY STRENUOUS .... 2<br>VERY STRENUOUS................................ 3<br>DON'T KNOW .... 8 |__|
19 |__|__| START YEAR<br>DON'T KNOW 98	|__|__| # YEARS<br>DON'T KNOW 98	LIGHT .... 1<br>MODERATELY STRENUOUS .... 2<br>VERY STRENUOUS................................ 3<br>DON'T KNOW .... 8 |__|

SECTION P. PHYSICAL MEASUREMENTS

NOTE: EACH MEASUREMENT IS TO BE TAKEN TWICE, AND THE DIFFERENCE BETWEEN THE MEASUREMENTS IS TO BE COMPUTED. IF THE DIFFERENCE EXCEEDS THE TOLERANCE LIMITS GIVEN BELOW, A THIRD MEASUREMENT IS TO BE TAKEN AND RECORDED.

P1. Standing height (cm)

FIRST	SECOND	DIFFERENCE	TOLERANCE	THIRD	
|__|__|__|.|__|	|__|__|__|.|__|	|__|__|__|.|__|	2.0 cm	|__|__|__|.|__|	|__|__|__|.|__|

P2. Waist circumference (cm)

FIRST	SECOND	DIFFERENCE	TOLERANCE	THIRD	
|__|__|__|.|__|	|__|__|__|.|__|	|__|__|__|.|__|	2.0 cm	|__|__|__|.|__|	|__|__|__|.|__|

P3. Hip circumference (cm)

FIRST	SECOND	DIFFERENCE	TOLERANCE	THIRD	
|__|__|__|.|__|	|__|__|__|.|__|	|__|__|__|.|__|	2.0 cm	|__|__|__|.|__|	|__|__|__|.|__|

P4. Weight (kg)

FIRST	SECOND	DIFFERENCE	TOLERANCE	THIRD	
|__|__|__|.|__|	|__|__|__|.|__|	|__|__|__|.|__|	1.0 kg	|__|__|__|.|__|	|__|__|__|.|__|

P5. Earwax examination (EXAM BY OTOSCOPE).

WET.. 1
DRY.. 2
MIXTURE.. 3
REFUSED 4
CANNOT CLASSIFY................................ 5 |__|

This concludes the interview. We would like to thank you very much for your time and effort in answering our questions.
