# Supplementary material for: Diet and biliary tract cancer risk in Shanghai, China
Source: PLoS One. 2017 Mar 13;12(3):e0173935. doi: 10.1371/journal.pone.0173935 (PMC5348031; doi:10.1371/journal.pone.0173935)
Supplement: S2 File — (PDF) [file pone.0173935.s004.pdf]

COVER PAGE

The cover page of the questionnaire is very important. It contains space for a subject identification number, the hospital name and identification number (if applicable), the date and the time the interview began, and your identification number. Unless otherwise specified the items that appear on the cover page are to be completed prior to the interview. Below is a description of these items:

- ID - Record the subject's assigned identification number.

- Hospital ID - This is the name and identification number of the hospital where the case or hospital control is identified. Write the name of the hospital on the line and its three digit identification number in the boxes provided. Note that this identification number is not applicable to population controls.

- Date - Enter the date the interview was conducted in the form year-month-day. Right justify and zero fill, as necessary, for each part of the date. For example, if the interview date was June 12, 1996, record the date as |9|6|-|0|6|-|1|2| in the six spaces provided.

- Time Began - Record the actual time you begin the interview. Also, circle "1" for a.m. or "2" for p.m. Right justify and zero fill as necessary. For example, if you begin asking the subject questions at 09:45 a.m., record the hour and minutes as |0|9|:|4|5| in the four spaces provided and circle "1" for a.m.

- Interviewer ID - Record your two digit interviewer identification number. You will receive this number at training.

QUESTION-BY-QUESTION SPECIFICATIONS
FOR
THE NUTRITION AND HEALTH STUDY IN SHANGHAI QUESTIONNAIRE

SUBJECT INFORMATION SHEET

The subject information sheet contains space for recording the name, address, work unit, and address of the work unit of the subject. There is also space for this same information to be recorded for the subject's next-of-kin. The items that appear on this sheet are to be recorded as completely as possible prior to the interview.

1. Write the subject's full name.

2. Record the district, street, house number, telephone number, and zip code of the subject's residence.

3. Record the name of the subject's work unit.

4. Record the district, street, house number, telephone number with the extension, and zip code of the subject's work unit.

5. Write the full name of the subject's next of kin who is best able to provide accurate information for the subject in the event the subject is permanently unavailable to be interviewed. The next-of-kin may be the subject's mother, father, sister, brother, aunt, uncle, or other close relative.

6. Write the next-of-kin's relationship to the subject.

7. Record the next of kin's address in the same way as for the subject in number 2.

8. Record the name of the next of kin's work unit.

9. Record the address of the next of kin's work unit in the same way as for the subject in number 4.

SECTION A. DEMOGRAPHIC INFORMATION

INTRODUCTION: The introduction is important as it will introduce you to the subject. It also explains the type of questions that will be asked during the interview, and lets the subject know that all answers to these questions will be kept confidential. Read this introduction to the subject verbatim.

Introductory sentences, or lead-in sentences, are printed throughout the questionnaire and should be read to the subject verbatim. These statements provide smooth transitions from one section to another. The lead-in to this section will let the subject know that questions will be asked about his/her background.

A1 Record the sex of the subject. Circle “1” for male or “2” for female. Ask this question only if necessary.

A2 Record the subject’s date of birth in the form year-month-day. Be sure to right justify the numbers for each part of the date, including leading zeros if necessary. If any part of the birth date is estimated, write “est.” to the right of the boxes.

A3 Circle the appropriate code for whether the subject’s date of birth (given in A2) is based on the lunar or Western calendar.

A4 Record the subject’s age at his/her last birthday. Verify that the age reported corresponds to the date of birth (given in A2) by subtracting the date of birth from the current date. Probe, if necessary, to correct any inconsistencies.

A5 Write in the location (province, city, and county) where the subject was born. For subjects born in China, record all items. For subjects born outside of China, record the name of the country.

A6 Circle the code that corresponds to the highest level of schooling the subject completed, regardless of skipped or repeated grades. For example, if the subject completed only some of senior middle school, circle the code for junior middle school since this was the highest level of schooling completed. Any completed post-graduate work would be coded as "above college." If the subject has completed another type of schooling than those specifically listed, code "other" and write the type of schooling specified by the subject on the line provided.

A7 The responses for this question are to be read to the subject in the order listed. Circle the code corresponding to the answer given by the subject.

SECTION B. SMOKING HISTORY

In this section of the questionnaire, questions are being asked about the subject's history of smoking cigarettes.

Lead-in Sentence: Read to the subject as written.

B1 If the subject has ever smoked 100 cigarettes or more at any time in his/her lifetime the answer should be coded "yes," even if the subject is not currently smoking. Note that 100 cigarettes is equivalent to 5 packs. If the answer is "yes," continue with B2. If the answer is "no" or "don't know" skip to Section C.

B2 If the subject ever smoked at least one cigarette per day for a period of at least 6 consecutive months, code "yes" and continue with B3. If "no" or "don't know," skip to section C.

B3 Record the subject's age when he/she first started smoking at least one cigarette per day. If the subject is unable to provide an age but gives the answer in terms of a calendar year, record the year to the left of the question. When the questions are coded later, this year will be used to calculate the subject's age.

B4 If the subject currently smokes at least one cigarette per day, code "yes" and skip to B6. If it is unknown whether the subject is currently smoking at least one cigarette per day, code "don't know" and skip to B6. If the answer is "no," continue with B5.

B5 Record the subject’s age when he/she last stopped smoking cigarettes regularly, that is, stopped smoking at least one cigarette per day. If the answer is given in terms of a calendar year, write the year to the left of the boxes for age.

B6 Record the actual number of years the subject smoked cigarettes or has been smoking cigarettes regularly. This number should not include periods of time when the subject was not smoking or was smoking less than one cigarette per day.

B7 Record the average or usual number of cigarettes the subject smoked per day, considering all of the years that he/she smoked.

SECTION C. BEVERAGE CONSUMPTION

In this section, questions are being asked about the subject’s history of drinking alcoholic beverages and tea. Note, that any question asking whether the subject consumed a particular beverage “on a regular basis” or “regularly” means having consumed one or more drinks of that beverage per week for a period of at least 6 consecutive months. Also, any question referring to “adult life” or “as an adult” means 18 years of age and older.

Lead-in Sentence: Read to the subject as written.

C1 Code "yes" if the subject in his/her entire lifetime, ever drank any type of alcoholic beverage (beer, wine, or liquor) regularly. If the answer is "yes,” continue with C2. If "no" or “don’t know,” skip to C8.

C2 Record the subject’s age when he/she began to drink alcoholic beverages regularly. Probe to get the most accurate response. If the answer is given in terms of a calendar year, write the year to the left of the boxes for age.

C3 We are interested in whether the subject still drinks regularly. If the response is "yes" or “don’t know,” skip to C5. If "no,” continue with C4.

C4 Record the subject's age when he/she stopped drinking alcoholic beverages regularly. If the subject gives the answer in terms of a calendar year, write the year to the left of the boxes for age.

C5 Record the total number of years the subject drank alcoholic beverages regularly. Periods of time when the subject did not drink alcohol at least once per week should not be included in the total.

C6 & C7 These 2 questions are combined into a table to obtain information about 4 different types of alcoholic beverages, numbered 1-4, that the subject may have consumed in his/her adult life.

In C6, record the usual number of bottles or liang of the particular beverage the subject consumed per week during his/her adult life. Also, remember to circle the appropriate code for "Bottles" or "Liang." If necessary, ask the subject to verify whether the number given was for bottles or liang. Then, go to C7 before moving on to the next beverage listed in C6. In C7, record the number of years that the subject consumed this type of beverage (excluding periods of time when he/she was not drinking this beverage). Continue in this manner for each beverage listed.

If the subject did not consume a particular beverage or it is not known if he/she did, circle the appropriate code for either "none" or "don't know," then skip C7 for that beverage and go to the next beverage listed under C6. For the last beverage listed, hard liquor, skip to C8 if the response to C6 is "none" or "don't know."

C8 If the subject ever drank any type of tea regularly in his/her adult life, code "yes" and continue with C12. If "no" or "don't know," skip to C15.

C9 Record the subject's age when he/she started to drink tea regularly.

C10 We are interested in whether the subject currently drinks any type of tea. Circle the appropriate code for the given response.

C11 Record the total number of years the subject has been drinking or drank tea regularly. Periods of time when the subject did not drink tea at least once per week should not be included in the total.

C12 Circle the code for the type of tea that the subject usually or most often drank in his/her adult life. If the subject usually drank green and black tea in equal amounts, circle the code for "both of the above." If another kind of tea was consumed most often, have the subject specify what kind it was. Then, circle the code for "other," and in the space provided write in the type of tea specified by the subject.

C13 We are interested in how the subject usually drank his/her tea. Read the possible responses, "strong," "medium," or "weak," to the subject so that he/she may choose the most appropriate response. Circle the code which corresponds to the answer given.

C14 Record the average number of liang of tea the subject drinks in the period of a week, month, or year, in the boxes provided. Be sure to also circle the code corresponding to the time frame in which the answer was given.

C15 We are interested in whether, during his/her adult life, the subject ever drank ginseng tea or used ginseng regularly. If "yes," continue with C16. If "no" or "don't know," skip the remaining questions in Section C and go to Section D.

C16 Read the possible responses to the subject so he/she may choose the most appropriate answer. Circle the appropriate code for whether the subject usually, or most often, drank ginseng tea (code "1") or used ginseng (code "2"). If the subject drank ginseng tea as often as he/she used ginseng, circle code "3." If the subject did not use ginseng, skip the remaining questions in Section C and go to Section D. Otherwise, continue with C17.

C17 We are interested in the type of ginseng the subject usually had. Read the possible responses to the subject so he/she may choose the most appropriate answer, and circle the corresponding code. If the subject usually had a type of ginseng other than white, red, or foreign, circle the code for "another type of ginseng" and specify the type in the space provided.

C18 Record the usual number of liang of ginseng the subject had in the period of a week, month, or year. Write the number in the boxes provided and circle the code for the time frame in which the answer was given.

SECTION D. MEDICAL HISTORY

In this section of the questionnaire, questions are being asked about medical conditions the subject may have had. Also, questions are asked about medications the subject may have taken.

Lead-in Sentence: Read to the subject as written.

D1-D3 We are interested in medical conditions of the subject's that were diagnosed by a doctor. For each medical condition in D1 (labeled a-t) for which a "yes" response is given, ask D2 and D3 before moving on to the next condition listed under D1. Whenever a "no" or "don't know" response is given, skip D2 and D3 and go immediately to the next condition listed under D1. If a "no" or "don't know" response is given to the last condition listed (item t, Cystic fibrosis), go to D4.

D1 We are interested in medical conditions of the subject's that were diagnosed by a doctor. Record whether the subject was ever diagnosed by a doctor as having a particular medical condition in his/her lifetime. If the subject believes that he/she has a particular condition but was never formally diagnosed by a doctor with the condition, this does not qualify as a "yes" response. If the subject is not familiar with the name of a particular condition, do not define the condition for the subject. Individuals who have been diagnosed by a doctor with the condition should recognize the name. If the subject does not recognize the name, most likely, the condition was never diagnosed by a doctor.

For item d, Hirsutism, you may read the definition given in parentheses next to the name of the condition aloud to the subject. For item r, if the subject has been diagnosed with any other digestive tract disease than those already asked about in items g-q, code "yes," then probe for the name of the condition and write it verbatim on the line provided.

D2 For each condition under D1 for which the subject answers "yes," record the subject's age when he/she was first diagnosed with the condition by a physician. If the answer is given in terms of a calendar year, write the year to the left of the space for age.

D3 For each condition under D1 for which the subject answers "yes," record whether the subject has ever been hospitalized for any reason directly related to the particular condition.

D4-D8 In this set of questions we are interested in medical conditions related to the liver and biliary cirrhosis. For each condition in D4 (labeled a-g), for which a “yes” response is given, ask D5-D8 before moving on to the next condition listed under D4. Whenever a “no” or “don’t know” response is given, skip D5-D8 and go immediately to the next condition listed under D4. If a “no” or “don’t know” response is given to the last condition listed, item g, go immediately to Box D-1. Otherwise, complete D5-D8 before going to Box D-1.

D4 This question is to be asked using the same guidelines as for D1.

For item f, if the subject has been diagnosed with a liver disease other than those already asked about in items a-e, code “yes,” then probe for the name of the condition and write it verbatim on the line provided.

D5 This question is to be asked using the same guidelines as for D2.

D6 This question is to be asked using the same guidelines as for D3.

D7 Record whether the subject has ever been prescribed any medicine (including herbal medicine) for the particular condition, by a doctor or other health care worker. If the response is “no” or “don’t know,” skip D8 and go to the next condition listed under D4.

For a “no” or “don’t know” response to item g in D7, go immediately to Box D-1.

D8 If the subject answers “yes” to D7, probe for the names of all of the medicines the subject took for the particular condition and write them verbatim on the lines provided.

BOX D-1 Refer back to the response given in D4d. If the subject answered “yes” to having chronic hepatitis, ask D9. If the subject answered “no” or “don’t know” to D4d, place a check mark in the box where indicated, skip D9 and go to D10.

D9 If the subject has chronic hepatitis, as indicated by a “yes” response in D4d, circle the appropriate code for whether he/she is also a chronic carrier of hepatitis.

D9a We are interested in the type of chronic hepatitis the subject was diagnosed as having. Circle the appropriate code for all responses that apply.

D10 Record whether the subject was ever diagnosed by a doctor as having gallstones. If "yes," continue with D11. If "no" or "don't know," skip to D28.

D11 Record the subject's age when he/she was first told by a physician that he/she had gallstones. If the answer is given in terms of a calendar year, write the year given to the left of the boxes for age.

D12 We are interested in the methods used to diagnose the subject's gallstones. Read each possible method listed (a-h) to the subject, coding a "yes," "no," or "don't know" response for each. Allow the subject to respond to each method before proceeding to the next method listed. For item h, if the subject was diagnosed using a method other than those listed in a-g, code "yes," then probe for the type of method used and write the answer verbatim on the line provided.

D13 We are interested in whether the subject ever had any of the symptoms, listed as items a through i, specifically associated with him/her having gallstones. Each symptom listed is to be read to the subject. Record a "yes," "no," or "don't know" response from the subject for a particular symptom before moving on to the next one listed.

D14 Record whether the subject has ever been hospitalized due to his/her gallstone condition. If "yes," continue with D15. If the subject answers "no" or "don't know," skip to D18.

D15 Record the number of times in the course of the subject's life that he/she was hospitalized because of gallstones. Remember to right justify and zero fill as necessary when writing the number in the boxes.

D16 Record the date when the subject was hospitalized the first time because of gallstones. Probe to get the most accurate answer.

If the subject is able to give only a partial date, leave the unknown or uncertain part of the date blank, and write what the subject says verbatim in the space near the boxes. For example, if the subject knows the year and month that the hospitalization occurred, but is unable to specify

the day and says "I think it was in the beginning of the month," record the year and month in the appropriate boxes and leave the boxes for the day blank. Then, write what was said by the subject in the space near the boxes.

D17 Record the name of the hospital and its location (province) where the subject was first hospitalized for gallstones.

D18 If the subject has ever been diagnosed by a doctor as having an obstruction of the bile duct because of gallstones, code "yes" and continue with D19. If the subject answers "no" or "don't know," skip to D21.

D19 Record the subject's age when he/she was first told by a doctor that he/she had an obstruction of the bile duct caused by gallstones. If the answer is given in terms of a calendar year, write the year to the left of the boxes for age.

D20 Record the number of times the subject experienced bile duct obstruction caused by gallstones, over the course of his/her life.

D21-D23 These questions are related to surgery the subject may have had involving the gallbladder and/or extrahepatic bile ducts.

Read each of the surgeries to the subject, listed as a-c in D21. For a "yes" response to a particular surgery, ask D22 and D23. In D22, record the complete date that the surgery was performed. Refer to D16 for guidelines regarding the recording of dates. In D23, write the name of the hospital and the province where it is located on the lines provided.

For a "no" or "don't know" response to a particular surgery in D21, skip D22 and D23 and go immediately to the next surgery listed in D21.

D24 We are interested in whether the subject was asked to adhere to a specific diet because of having gallstones.

D25 If the subject was ever prescribed any medicine by a doctor, including herbal medicine, to help dissolve or eliminate his/her gallstones, code “yes” and continue with D26. If “no” or “don’t know,” skip to D27.

D26 Write the name(s) of the medication(s) prescribed to the subject to dissolve his/her gallstones verbatim on the lines provided.

D27 We are interested in whether the subject ever had a catheter (tube) put directly into his/her gallbladder and received an injection of medicine to dissolve the gallstones. Circle the appropriate code.

D28 If the subject was ever diagnosed by a doctor with any gallbladder disease other than gallstones, code “yes” and continue with D29. If “no” or “don’t know,” skip to D31.

D29 & D30 We are interested in the type(s) of gallbladder disease the subject was diagnosed by a doctor as having, other than gallstones. Read to the subject each of the gallbladder diseases listed as items a-d in D29, allowing the subject to respond before proceeding. For any “yes” response, go to D30 and record the subject’s age when he/she was first told about having the disease. For a “no” or “don’t know” response to a particular disease, skip D30 and go to the next disease listed in D29.

For D29d, if the subject indicates that he/she has had any type of gallbladder disease other than gallstones, or those conditions listed as items a-c, probe to identify the specific disease and write it verbatim on the line provided.

D31 & D32 We are interested in whether the subject was ever diagnosed by a doctor as having typhoid fever or paratyphoid fever.

If the subject has been diagnosed with a particular disease in D31, code “yes,” then go to D32 and record his/her age when he/she was first diagnosed with that disease. If the response is “no” or “don’t know” for a particular disease, skip D32 and go to the next disease listed or to D33.

D33 We are interested in whether the subject was ever diagnosed by a physician as being a chronic carrier of typhoid. As a chronic carrier, the individual would harbor the bacteria after having recovered from the disease or because he/she is immune to the disease and disseminate the typhoid bacteria to others, causing their infection.

D34 If the subject, in his/her lifetime, was ever diagnosed by a doctor as having diabetes, code "yes" and continue with D35. All types of diabetes (juvenile, adult-onset, and pregnancy-associated) should be considered in the subject's response. If the response is "no" or "don't know," skip to D47.

D35 Record the subject's age when he/she was first diagnosed with diabetes by a doctor. If the answer is given as a calendar year, write the year to the left of the boxes for age.

D36 We are interested in the type of diabetes the subject was told he/she had when first diagnosed. Do not read the possible types listed aloud to the subject, however, you may probe to obtain the most accurate response.

D37 If the subject ever took insulin to treat his/her diabetes, code "yes" and continue with D38. If the response is "no" or "don't know," skip to D40.

D38 Record the subject's age when he/she was first given insulin. If the answer is given as a calendar year, write the year to the left of the boxes for age.

D39 Record the total number of years that the subject was treated with insulin. Do not include in this number any periods of time during which the subject was not receiving insulin injections. Probe if necessary to get the most accurate response.

D40 If the subject was ever prescribed any type of pill by a health care professional to treat his/her diabetes, code "yes" and continue with D41. If the response is "no" or "don't know," skip to D42.

D41 Circle all codes that apply for each different pill that the subject was treated with for diabetes. If the subject was treated with a pill other than those specifically named, circle the code for "other" and write in the name of the pill that is specified by the subject verbatim.

D42 We are interested in whether the subject was ever admitted to a hospital because of his/her diabetes. This would include hospitalization for treatment, as well as because of a health problem associated with the diabetes. If "yes," continue with D34. If the response is "no" or "don't know," skip to D45.

D43 Record the total number of times that the subject was hospitalized for complications or treatment of diabetes.

D44 Write the name and location (province) of each hospital where the subject was admitted because of his/her diabetes, on the lines provided. Record the year of the hospitalization, in the boxes provided. Probe to get the most accurate response.

Record each hospitalization separately. If more than one hospitalization for diabetes occurred at the same hospital, be sure to write separate entries for these, even if the hospitalizations occurred in the same year. If two or more entries are identical because of this, put a note in the margin explaining the situation.

D45 & D46 We are interested in whether the subject was ever told by a doctor that he/she had complications of diabetes which involved any of the body organs and conditions listed (items a-g) in D45. Only record a "yes" response if the complication was diagnosed by a physician as being a result of the diabetes. If the response is "yes," go to D46 and record whether the subject was hospitalized for the particular condition, before going on to the next organ/condition listed. If the response in D45 is "no" or "don't know," skip D46 and go to the next organ/condition listed in D45.

D47-D50 We are interested in whether the subject has ever had any of the types of gastrointestinal surgery listed as items a-d in D47. If so, we are also interested in the reason for having the surgery, and where and when it took place. If the subject has had a particular type of surgery as indicated by a "yes" response in D47, ask D48-D50 before going to the next surgery listed. If the response is "no" or "don't know," go immediately to the next type of surgery listed in D47.

D47 Circle the appropriate code for the response given by the subject for each surgery listed.

Note that item a, complete or partial gastrectomy, is the surgical removal of all or part of the stomach. If the subject indicates that he/she had all or part of his/her stomach removed, but did not know that this was a gastrectomy, the response should be coded as a "yes." Surgery on the small bowel is equivalent to that on the small intestine. Surgery on the large bowel is equivalent to that on the large intestine or colon, and includes the rectum.

D48 Probe to get the specific reason or condition that led to the subject having the particular surgery. Write the response verbatim on the line provided.

D49 Record the year that the particular surgery took place. If the subject had surgery more than one time on any particular organ listed, record only the year that the first surgery took place. If the answer is given as the subject's age, write the age given to the left of the boxes for the year.

D50 Write the name and province (location) of the hospital where the particular surgery was performed. If the subject had surgery more than one time on any particular organ listed, record only the name of the hospital, and its location, where the surgery took place the first time.

D51 We are interested in whether the subject was ever diagnosed by a physician as having any type of thyroid disease, prior to the past 12 months. Thyroid diseases include Grave's disease or hyperthyroidism, Hashimoto's disease, hypothyroidism, benign adenoma or nodule, thyroid carcinoma, or goiter. If the response is "yes," continue with D52. If the response is "no" or "don't know," skip to D55.

D52 Circle the appropriate code for the specific type of thyroid disease with which the subject was diagnosed. If the subject does not know the name of the thyroid disease, but is able to describe it, write the description verbatim in the space to the left of the responses and do not circle any code.

D53 Record the subject's age when he/she was first told by a doctor that he/she had a thyroid disease. If the answer is given as a calendar year, write the year in the space to the left of the boxes for age.

D54 We are interested in the type of treatment the subject received for his/her thyroid condition. Read each of the possible types of treatments listed (a-c) to the subject. Each of the treatments requires a response from the subject before going to the next one listed.

D55 Record whether the subject was ever told by a physician that he/she had any type of cancer or a tumor, prior to the past 12 months. If "yes," continue with D56. If "no" or "don't know," skip to D59.

D56 Record the subject's age when he/she was first told by a physician that he/she had cancer or a tumor. If the answer is given as a calendar year, write the year to the left of the boxes for age.

D57 We are interested in the part of the body the first diagnosed cancer or tumor was located. Probe to get the most specific answer as possible. For example, if the subject states that he/she had cancer in the abdomen, probe to identify whether the cancer was specifically in the stomach, colon, or pancreas. If the subject does not know specifically, write verbatim what information is obtained.

D58 Write the name and location (province) of the hospital where the subject's cancer or tumor was first diagnosed.

D59-D63 We are interested in different types of medicines (in pill or injection form) that the subject may have used in his/her lifetime. Read to the subject each type of medicine, listed as items a-c, in D59. If a "yes" response is given for a particular type of medicine listed in D59, ask D60-D63 before going on to the next medicine. If a "no" or "don't know" response is given, go immediately to the next medicine listed.

D59 Circle the appropriate code for the response given by the subject for each type of medicine listed. For D59c, if the subject indicates that he/she has taken a medicine for ulcers, probe to get the name of the medicine and write it verbatim on the line provided.

D60 Write verbatim the specific reason or condition for which the subject took the particular medicine.

D61 Record the subject's age when he/she first used the particular medicine. If the subject gives the answer in terms of a calendar year, write the year just below the boxes for age.

D62 Record the subject's age when he/she last used the particular medicine. If the subject gives the answer in terms of a calendar year, write the year just below the boxes for age.

D63 Record the total length of time that the subject took the particular medicine. Do not include periods of time when the medicine was not taken in the total. The response may be given in days, weeks, months, or years--whichever is easiest for the subject to respond. Be sure to circle the code for the time frame used by the subject.

D64 If the subject has ever taken aspirin or any aspirin containing products regularly (at least two times a week for one month or longer), prior to the past 12 months, code “yes” and continue with D65. If the response is “no” or “don’t know,” skip to D69.

We are specifically interested in aspirin and aspirin containing products, examples of which are Bayer aspirin, Bufferin, Ecotrin, Excedrin, Ascriptin, and Alka-Seltzer Antacid and Pain Reliever. Note that other pain relievers or anti-inflammatory drugs, such as acetaminophen (Tylenol) or ibuprofen (Motrin, Advil), are not to be considered in this category.

D65 Record the subject’s age when he/she first started to take aspirin or aspirin-containing products regularly (at least two times a week for one month or longer).

D66 Record the subject’s age when he/she last took aspirin or aspirin-containing products regularly (at least two times a week for one month or longer). If the subject is still using aspirin regularly, at least two or more times a week, circle code “00.”

D67 Record the number of aspirin or aspirin-containing tablets (pills) that the subject usually took each day or each week, during the time when he/she was taking aspirin regularly. Write the number given in the boxes provided, and circle the code for the appropriate time frame.

D68 Record the total number of months or years that the subject took aspirin or aspirin-containing products on a regular basis. Exclude periods of time in the total when aspirin was not taken regularly.

D69 We are interested in the subject’s facial skin type, in general, during his/her adult life. Read each of the possible skin types to the subject before allowing the subject to respond.

You may probe to get the most accurate response. The following guidelines may be helpful in defining each of the skin types listed. If the subject’s facial skin as an adult typically was oily all over, then “oily” should be coded. If it was typically dry all over, “dry” should be coded. If the subject’s facial skin typically had both oily and dry areas, “a combination” should be coded. Or, if it was typically neither oily nor dry, “normal” should be coded.

D70 We are interested in whether the subject has had severe chronic acne during his/her adult years. Acne is a skin disorder caused by inflammation of the skin glands and hair follicles, and is marked by pimples. Do not offer your opinion as to whether the subject's acne condition, if described to you, should be considered a severe chronic case. If the subject has had acne as an adult, he/she must determine whether it was a severe chronic case.

If the subject has had severe chronic acne as an adult, code "yes" and continue with D71. If the response is "no" or "don't know," skip to D73.

D71 If the subject has ever used any medication specifically for treating acne during his/her lifetime, code "yes" and continue with D72. If the response is "no" or "don't know," skip to D73.

Birth control pills are sometimes prescribed to treat acne in females. In this case, only if the subject was prescribed birth control pills specifically to treat her acne condition should "yes" be coded. If the subject was taking birth control pills for the purpose of birth control or to regulate her periods, but the pills in addition helped to control her acne, code a "no" response since the pills were not being taken specifically for treating acne. If the subject was taking birth control pills for the purpose of birth control or to regulate her periods, and also for the purpose of treating acne, code a "yes" response.

D72 Record the total number of days, weeks, months, or years (whichever is easiest for the subject) that the subject used medication for acne. Exclude periods of time in this total when this medication was not taken. Write this number in the boxes provided, and circle the code for the time frame used by the subject.

D73 We are interested in the usual consistency and color of the subject's earwax. Read the possible responses to the subject before allowing him/her to respond. Circle the appropriate code.

D74 We are interested in the subject's usual hair type during his/her adult life--oily, dry, a combination, or normal. Read the possible responses to the subject before allowing him/her to respond. The same definitions, written in D69, for facial skin type can be applied here for hair type.

D75 Record whether the subject believes that during his/her adult life, that he/she sweats (or perspires) more, less, or the same as an average adult of the same sex.

D76 Record whether the subject believes that the palms of his/her hands sweat (or perspire) a lot in the summer months. The subject must make his/her own determination about this. Do not probe or give your own opinion.

D77 We are interested in how frequently during the summer months the subject needs to wipe sweat or perspiration off of his/her face with a handkerchief or other cloth. Read the possible answer categories to the subject before allowing him/her to respond.

D78 We are interested in how the subject believes his/her body odor is, in general, in comparison to other persons of the same age and sex. Read the possible answer categories to the subject before allowing him/her to respond.

D79 Record whether the subject, during his/her adult life, has used a deodorant or antiperspirant product.

SECTION E. DIET HISTORY

This section of the questionnaire contains statements which are to be read verbatim to the subject to introduce and explain what will be asked in a particular set of questions. These statements will also help to clarify for the subject the time period to which each set of questions is referring.

Many of the questions in this section refer to usual "dietary habits," which means what was done most often. Also, many questions refer to the subject's "adult years" which means 18 years of age and older. In addition, many questions in this section refer to the subject's dietary habits before 5 years ago. If the subject's dietary habits changed in the past 5 years, these changes are not to be considered in the answers.

Due to the repetitive pattern of the questions, this section could become tedious for both the interviewer and the subject. However, remember that diet and nutrition are important factors intimately

related to health. Try to keep the questions flowing as smoothly as possible. Show Card A, which is a list of possible answers for certain questions, is to be used at designated questions to remind the subject of the possible responses to the selected questions.

Lead-in Statement: Read to the subject as written.

E1 Record how often the subject eats each different type of food listed (numbered 1-4). Circle the code which corresponds most closely to the answer given by the subject for the particular type of food, before moving on to the next food item listed in the table.

Note that item E1.3, cured foods, are those foods which are preserved with nitrite, not just salt. Examples are Nanjing duck, Ning Bao crabs, dry fish, and ham.

E2 We are interested in how the meat that the subject ate as an adult, before 5 years ago, was usually prepared. Do not include poultry in this category. When reading the question show the subject Card A. Have the subject choose all the answers that apply, and circle the appropriate codes. If any other cooking method was usually used, code “other,” and write the method specified verbatim in the space provided.

If the subject did not eat meat (code “00”) or does not know which cooking method was usually used (code “98”), skip to E7. Otherwise, continue with E3.

E3 Ask this question only if more than one cooking method was circled in E2. We want to know which of the methods circled in E2 was used most often to cook meat. When asking the question, read back to the subject the answers he/she gave in E2 so that one of these is chosen for the answer to E3. Only one answer is acceptable. If the cooking method used most often was that which was specified as “other” in E2, then code “other” and write the method used as it was written in E2.

If only one method was circled in E2, do not ask this question, and skip to E4.

E4 We are interested in whether the subject, before 5 years ago, as an adult, usually ate the fat when eating beef or pork. Do not include bacon or sausage in the category of beef or pork.

Circle the code which corresponds to the subject's response. If the subject never ate beef or pork, circle code "0."

E5 Record the number of times per day, week, month, or year the subject ate vegetables cooked with salt pork, ham hocks, bacon fat, or fat-back. These food items, typically, are cooked with vegetables to add flavoring. Also, circle the code for the time period specified by the subject. The subject may answer using any time period he/she wishes. If vegetables were not eaten this way by the subject, code "never."

E6 Record the number of times per day, week, month, or year the subject ate rice that was mixed with meat drippings (juices or fat from meat). Also, circle the code for the appropriate time period. The subject may answer using any time period he/she wishes. If rice was not eaten this way by the subject, code "never."

E7 Use Show Card A. This question about eating poultry is to be answered in the same manner as question E2. If the subject did not eat poultry (code "00") or does not know which cooking method was usually used (code "98"), skip to E10.

E8 This question is to be answered in the same manner as question E3. If only one method was circled in E7, do not ask this question, and skip to E9.

E9 Record whether the subject most often ate poultry with or without the skin. If poultry was eaten by the subject an equal number of times with the skin as without the skin, code "both equally."

E10 Use Show Card A. This question about eating cooked fish is to be answered in the same manner as E2. If the subject did not eat cooked fish (code "00") or does not know which cooking method was usually used (code "98"), skip to E12.

E11 This question is to be answered in the same manner as E3. If only one method was circled in E10, do not ask this question, and skip to E12.

E12 Consider only meals the subject ate in which meat, poultry, or fish was mixed together with vegetables. We want to know what the usual proportion was of meat, poultry, or fish compared

to the proportion of vegetables in these meals. Read the possible responses to the subject before allowing the subject to respond. Circle code “0” if the subject did not ever eat meat, poultry, or fish mixed together with vegetables.

E13 Consider only meals in which the subject ate vegetables alone, i.e., not mixed with other food items. We are interested in how the vegetables were usually prepared. Read the possible responses to the subject before allowing the subject to respond. If vegetables alone were usually cooked with something other than water or oil, circle code “6” and write verbatim the method specified by the subject. If the subject never ate vegetables by themselves, circle code “0.”

E14 Circle the code corresponding to the type of milk the subject usually drank as an adult, before the past 5 years. Do not read the possible answers to the subject. Probe for the best response. If the subject used to drink a type of milk not listed, circle the code for “other” and write in the type of milk specified by the subject verbatim.

Lead-in Statement: Read the introduction to the subject as written. In this next set of questions, it may not be possible for the subject to tell you exactly how often a food item was eaten by him/her or how much of it was eaten. Probe to get the best approximate answers if necessary. For a food item available only seasonally, have the subject tell you how often the item was eaten when in season and record this response.

E15 & E16 For each of the food items listed in E15 there is a corresponding question in E16. Each item listed in E15 requires a response. In E15, record the number of times per day, week, month, or year that a particular food item was eaten. The answer should be recorded using the time period specified in the subject’s response. If the food item was never eaten, place a check mark in the column labeled “never,” and go to the next food item listed in E15. If the food item was eaten, go to E16, and record how many liang were eaten each time the subject ate the food. If the response is "don't know" to how many liang, probe to find out whether an approximate number can be given. If not, place a check mark in the column labeled "don't know.” Then, go to the next food item listed in E15.

Note that there is a second part to E15.2, numbered E15.2a. If the subject ate salted fish, as indicated in E15.2, ask E15.2a. In E15.2a, record the kind of salty fish eaten most often by

the subject. That is, we are interested in the common name of the fish, such as mackerel or sardine. Otherwise, skip to E15.3.

Also, note that item E15.7, preserved eggs, means eggs that are preserved with lime.

E17 & E18 These questions about different meats, poultry, fish, and beans are to be answered in the same manner as in E15 and E16. For those items which are animal foods, we are only interested in fresh animal foods, not preserved. There are 32 food items listed in E17, each requiring a response. If the subject reports tht a food iten was eaten, ask E18a. If the response is “never” or “don’t know”, skip E18a and go to the next food item listed under E17.

For Items 1-11 only, E18 has two parts (“a” and “b”). For these eleven items, if the subject is able to provide the number of liang eatin, then ask E18b. In E18b, we are interested in whether the weight of the food item given in E18a was the weight before or after cooking the food item. Circle code “1” for before, or code “2” for after, then continue with the next food item in E17. If the answer in E18a is "don't know,” skip E18b, and continue with the next food item in E17.

Note that item E17.3, pig feet, means only the foot without the leg. Also, we are interested in fresh pig feet, not Tipang (i.e. not preserved).

Also note, for items E17.4 - E17.6, fresh pork, do not include pork chops and spareribs in this category. Pork chops and spareribs are asked about separately in E17.1 and E17.2.

E17 & E18 These questions about different meats, poultry, fish, and beans are to be answered in the same manner as in E15 and E16. For those items which are animal foods, we are only interested in fresh animal foods, not preserved. There are 32 food items listed in E17, each requiring a response. If the subject reports tht a food iten was eaten, ask E18a. If the response is “never” or “don’t know”, skip E18a and go to the next food item listed under E17.

For Items 1-11 only, E18 has two parts (“a” and “b”). For these eleven items, if the subject is able to provide the number of liang eatin, then ask E18b. In E18b, we are interested in whether the weight of the food item given in E18a was the weight before or after cooking the food item. Circle code “1” for before, or code “2” for after, then continue with the next food item

in E17. If the answer in E18a is "don't know," skip E18b, and continue with the next food item in E17.

Note that item E17.3, pig feet, means only the foot without the leg. Also, we are interested in fresh pig feet, not Tipang (i.e. not preserved).

Also note, for items E17.4 - E17.6, fresh pork, do not include pork chops and spareribs in this category. Pork chops and spareribs are asked about separately in E17.1 and E17.2.

E17 & E18 These questions about different meats, poultry, fish, and beans are to be answered in the same manner as in E15 and E16. For those items which are animal foods, we are only interested in fresh animal foods, not preserved. There are 32 food items listed in E17, each requiring a response. If the subject reports tht a food iten was eaten, ask E18a. If the response is “never” or “don’t know”, skip E18a and go to the next food item listed under E17.

For Items 1-11 only, E18 has two parts (“a” and “b”). For these eleven items, if the subject is able to provide the number of liang eatin, then ask E18b. In E18b, we are interested in whether the weight of the food item given in E18a was the weight before or after cooking the food item. Circle code “1” for before, or code “2” for after, then continue with the next food item in E17. If the answer in E18a is "don't know," skip E18b, and continue with the next food item in E17.

Note that item E17.3, pig feet, means only the foot without the leg. Also, we are interested in fresh pig feet, not Tipang (i.e. not preserved).

Also note, for items E17.4 - E17.6, fresh pork, do not include pork chops and spareribs in this category. Pork chops and spareribs are asked about separately in E17.1 and E17.2.

E19 & E20 These questions about staple foods are to be answered in the same manner as in E15 and E16. Each of the four food items listed requires a response.

Note, for Items 1 and 2 only, E20 has two parts (“a” and “b”). Refer to the note under E17 and E18 for guidelines.

E21 & E22 These questions about different vegetables are to be answered in the same manner as in E15 and E16. We are interested in fresh vegetables, not preserved. There are 49 food items listed, each requiring a response.

E21 & E22 These questions about different vegetables are to be answered in the same manner as in E15 and E16. We are interested in fresh vegetables, not preserved. There are 49 food items listed, each requiring a response.

E23 & E24 These questions about different kinds of fruit are to be answered in the same manner as in E15 and E16. Each of the 8 items listed requires a response.

Note, for Items 3, 4, 7, and 8, E24 has two parts ("a" and "b"). Refer to the note under E17 and E18 for guidelines. In E24b, however, we are interested in whether the weight (or liang) of the food item given in E24a included the outside skin or peel. If "yes" circle code "1." If "no," circle code "2."

E25 & E26 These questions about types of desserts are to be answered in the same manner as in E15 and E16. Each of the five items listed requires a response. Note that item E24.4, red bean soup, is the same as Azuki bean soup, and that item E24.5, green bean soup, is the same as Mung bean soup.

E27 Record how many liang of each food item was eaten by the subject per month, before 5 years ago. Be sure to emphasize this time period to the subject. If the subject is unable to give an answer in terms of a month, and gives an answer in terms of some other period of time, write the answer given, verbatim, to the left of that food item. Probe, if necessary, to get the most accurate response. If a particular food item was not eaten, write “00” in the boxes. Note that we are specifically asking for how many liang of each food item the subject alone ate, not the whole household.

E28 Record how many liang of each food item was eaten by the subject per month, before 5 years ago. Be sure to emphasize this time period to the subject. If the subject is unable to give an answer in terms of a month, and gives an answer in terms of some other period of time, write the answer given, verbatim, to the left of that food item. Probe, if necessary, to get the most accurate response. If a particular food item was not eaten, write “00” in the boxes. Note that we are specifically asking for how many liang of each food item the subject alone ate, not the whole household.

E29 & E30 These questions about fried foods are to be answered in the same manner as in E15 and E16. Each of the five items requires a response. Note that for items E29.1- E29.3, we are interested in chicken, fish, or pork that is breaded or battered, and then fried.

E31 The subject must determine whether his/her diet changed significantly in the most recent 5 years, compared to before 5 years ago. You may probe, but do not offer any opinions as to what may be considered a significant change in diet. If the answer is "yes,” circle code “1” and continue with E32. If "no,” circle code “2” and skip to E35.

E32 Probe to determine how the subject’s diet changed over the past 5 years. Circle all codes that apply. If the subject’s diet changed other than in one of the ways specifically listed, code “other”, probe to find out how the subject’s diet changed, and write what is said verbatim, on the line provided.

E33 Probe to determine why the subject’s diet changed over the past 5 years. Code only the one response. If the subject provides more than one reason for his/her change in diet, probe for the primary reason his/her diet changed.

E34 Record the year that the subject’s diet changed significantly in the spaces provided. Note that the year given as the answer should be within 5 years of the interview date, since we are interested in a change in diet that has occurred in the past five years, as indicated in E31.

Lead-in Sentence: The next set of questions (E35-E37) are about the subject’s use of vitamins, during his/her adult life, prior to 5 years ago. Read the lead-in to the subject verbatim.

E35-E37 For each type of vitamin listed in E35 (numbered a-k), there is a corresponding question in E36 and E37. If the subject took the particular type of vitamin asked about in E35, then E36 and E37 is asked before going on to the next vitamin listed in E35. If the subject did not take a particular type of vitamin, then go immediately to the next vitamin listed in E35.

E35 If the subject, during his/her adult life, took a particular type of vitamin at least one time a week for one year or more, code "yes" and ask questions E36 and E37. If "no,” go to the next vitamin listed.

Note, in E35a, if the subject had taken multivitamin pills, ask question E35a.1 before going to E36 and E37. Probe to get the specific name of the multi-vitamin taken most often, and write this name verbatim in the space provided in E35a.1.

E36 Record the total number of years that the subject took the particular type of vitamin regularly (at least once a week).

E37 Record the usual number of times per day or per week, before the past 12 months, that the subject took the particular vitamin. Also, circle the code corresponding to the time frame (per day or week) specified by the subject.

E35 If the subject, during his/her adult life, took a particular type of vitamin at least one time a week for one year or more, code "yes" and ask questions E36 and E37. If "no,” go to the next vitamin listed.

Note, in E35a, if the subject had taken multivitamin pills, ask question E35a.1 before going to E36 and E37. Probe to get the specific name of the multi-vitamin taken most often, and write this name verbatim in the space provided in E35a.1.

E36 Record the total number of years that the subject took the particular type of vitamin regularly (at least once a week).

E37 Record the usual number of times per day or per week, before the past 12 months, that the subject took the particular vitamin. Also, circle the code corresponding to the time frame (per day or week) specified by the subject.

SECTION F. MENSTRUATION AND MENOPAUSE HISTORY

This section contains questions on menstruation and menopause.

BOX F-1 Complete this section for female subjects only. If the subject is a male, skip to Section K.

Lead-in Sentence: Read to the subject as written.

F1 Record the subject's age when she had her first menstrual period. It is important to get a best guess if the subject can't remember exactly. Probe using events in her life, for example, "Do you recall what grade in school you were in when you first had your menstrual period?" If after probing she cannot even give an estimated age, circle code "98" for "don't know." If the subject never had a menstrual period, circle code "00" for "never" and skip to F6. Otherwise, continue with F2.

F2 Record the age when the subject's periods became regular. "Regular" means periods came at predictable intervals about once a month, and does not refer to amount of flow. If the subject has trouble recalling this, you will need to probe for the best answer. If after probing she cannot even give an estimated age, circle code "98" for "don't know." If the subject's periods never became regular, circle code "00" and skip to F4. Otherwise, continue with F3.

F3 We are interested in the subject’s usual cycle length during the time that her menstrual periods were regular. Record the response as a single number, e.g., 30 days, or a range of numbers, e.g., 28 to 30 days, whichever fits the response best. If a single number is given, enter the number in the first set of boxes.

F4 Record the year and month of the subject’s last or most recent menstrual period. Probe if necessary, to get the most accurate answer.

BOX Question F5 is only to be asked if the subject has not had a period in the past six months.

F-2 Refer to the response given in F4 to determine this. Skip to Question F6 if it has been less than six months since the subject’s most recent period.

F5 Record the reason the subject has not had a period for at least six months. If the subject states a reason other than menopause, surgery, or pregnancy, circle code “6” for “other” and write in the reason specified by the subject verbatim.

F6 We are interested in whether the subject has had either a partial or total surgical removal of her ovaries or uterus. Read the organs listed to the subject one at a time, allowing the subject to respond before proceeding to the next item listed. Circle the appropriate code indicating "yes" or "no" for each. Note that whether the surgical removal of these reproductive organs was partial or total, the answer would be "yes.”

If the subject does not know whether she had any of these surgically removed, do not probe, and circle “8” for "don't know." If the subject comments about a surgery she had, but is uncertain whether it involved her ovaries or uterus, write what she said verbatim in the space to the left of the responses.

SECTION G. CONTRACEPTIVE HISTORY

This section of the questionnaire asks about birth control methods (family planning, contraception) used by the subject and her partner(s).

BOX G-1 Complete this section for female subjects only. If the subject is a male, skip to Section K.

Lead-in Sentence: Read to the subject as written.

G1 We are interested in whether the subject and any sexual partner she has had, ever used a method of birth control for family planning. If "yes," continue with G2. If "no" or "don't know," circle the appropriate code and skip to G6.

If you sense that the subject does not understand what is meant by birth control or family planning, you might explain by saying that this would be something she or her partner did to prevent pregnancy.

G2 Show CARD B to the subject as you are reading the question. Allow the subject to choose all of the methods of birth control, listed as items 1-6 and written on Card B, that she and a sexual partner ever used. Circle the appropriate codes for each method ever used. If the subject and any of her sexual partners never used any of the types of methods listed, circle code “08” for “none of these methods.”

BOX G-2 Refer to the answer given for G2. If the subject never used birth control pills or oral contraceptives as a method of birth control, skip to G6. If birth control pills were used, continue with G3.

Lead-in Sentence: The next set of questions are specifically about the subject’s use of birth control pills. Read the lead-in to the subject verbatim.

G3-G5 We are interested in the names of the birth control pill(s) used by the subject, the age she began to use each one, and the age she stopped using each one. In the first column (G3) record the subject’s age when she first used birth control pills. In the next column (G4) write in the name of this pill verbatim in the space provided. In the next column (G5), record the subject’s age when she stopped taking this first pill.

If the subject has taken more than one kind of birth control pill, go to the next row, and fill in the information as you did for the first pill.

G6 If the subject ever took birth control pills for a reason other than birth control, code "yes" and continue with G7-G10. If "no" or "don't know", skip to Section H.

G7-G10 These questions are about birth control pills the subject used for a reason other than birth control. Questions G7, G8, and G10 are like Questions G3-G5 and should be asked in the same manner.

G9 is an additional question where the reason for the subject taking the birth control pill(s) is to be recorded. Do not read the possible responses to the subject. Let her tell you the reason, then circle all appropriate codes. If a reason is given which is not to "regulate periods" or for "acne," then circle the code for "other" and write in the reason verbatim in the space provided. Circle code "8" if the subject does not know the reason.

SECTION H. PREGNANCY HISTORY

In this section of the questionnaire, you will obtain information about the subject's pregnancies and about infertility.

BOX H-1 This section is to be completed for female subjects only. If the subject is a male, skip to Section K.

Lead-in Sentence: Read to the subject as written.

H1 If the subject has ever been pregnant, circle code "1" for "yes" and continue with H2. Even if a pregnancy did not result in a live birth, for example, due to a miscarriage or an abortion, the answer would still be "yes." If "no," circle code "2" and skip to H6. If the subject does not know or is uncertain if she was ever pregnant, circle code "8" and skip to H6.

H2 Record the total number of pregnancies the subject has had in the space provided. Include in this number all pregnancies whether or not they resulted in a live birth. Also include a current pregnancy.

Lead-in Statement: Read to the subject as written. It will inform her that the next few questions are about each pregnancy she has had.

H3 & H4 These questions are about the outcome of each pregnancy the subject had and about breast feeding. There is space in the table to record this information for up to 7 pregnancies. A supplemental sheet is provided for additional space if the subject had more than 7 pregnancies.

The pregnancies are numbered across the top of the page and should be recorded in chronological order, first to last. If, however, the subject reports them out of order, cross out the printed pregnancy number and write in the corrected one. For example, if after telling you about her second pregnancy, she remembers one that occurred between the first and second, record it in the place designated for the third pregnancy but change the numbering so it reads:

Preg 1 Preg 3 Preg 2

The current pregnancy should always be the last one entered in the grid.

When asking H3, use the word choices in parentheses to distinguish the pregnancy to which you are referring. The first time you read the question, it would thus be "Was the outcome of your first pregnancy a live birth, stillbirth...?"

If the pregnancy resulted in a “live birth,” circle code “1” and continue with H4. If there is more than one outcome of a pregnancy, for example, a live birth and a stillbirth if the subject was pregnant with twins, circle the corresponding codes for both “live birth” and “still birth” in the same column. Then, ask H4 for the live birth.

If the pregnancy resulted in a live birth as indicated in H3, ask H4, and record the number of months the subject breast fed this child in the boxes provided. If the subject did not breast feed this child, circle code “00.” If the subject does not know how long she breast fed this child, probe to get the best answer. If she does not know whether she breast fed this child, or cannot say for how long, circle code “98” for "don't know.” Continue with H3 and H4 in this manner for each pregnancy.

If a particular pregnancy resulted in anything other than a live birth, circle the most appropriate code number "2-7," and then ask about the next pregnancy. If the subject does not know/remember what the outcome was, circle code "8" and continue with the next pregnancy.

BOX H-2 If the subject had more than 7 pregnancies, go to Supplement Sheet 1 for additional space. Place a check mark in the box where indicated, if this is necessary.

BOX H-3 Check the outcome of each pregnancy listed in H3. If at least one of the subject's pregnancies resulted in a live birth, ask Question H5. If none of the subject's pregnancies resulted in a live birth, skip to H6.

H5. Record the subject's age when she had her first live birth.

Lead-in Sentence: The next set of questions are about difficulties the subject may have had in becoming pregnant or in maintaining pregnancies. Read the lead-in to the next set of questions to the subject verbatim.

H6 We are interested in whether the subject ever went to see a doctor, or went to a clinic or hospital, because she was having difficulties becoming pregnant. If "yes," circle code "1" and continue with H7.

If the subject never had difficulties becoming pregnant, circle code "2" for "no" and skip to H14. Code "no," even if the subject was having difficulties becoming pregnant, but never actually visited a doctor, clinic, or hospital because of this. If the subject does not know, circle code "8" and skip to H14.

H7 Record the subject's age when she first visited a doctor, clinic, or hospital because of having difficulty becoming pregnant.

H8 If a doctor was able to tell the subject why she was having difficulty becoming pregnant, code "yes" and continue with H9. If a doctor was not able to tell her why she was having difficulty becoming pregnant, or the subject does not know, circle the appropriate code and skip to H10.

H9 Ask about the nature of the subject's infertility problem and circle the codes for all responses that apply. If the problem is other than, or in addition to, the 6 problems listed, circle the code for "other" and write in the problem specified by the subject. If the subject does not know the reason for her infertility, circle code "98" for "don't know."

H10 If the subject ever took medications or hormones to help her become pregnant, code "yes" and continue with H11-H13. If "no" or "don't know," circle the appropriate code and skip to H14.

H11-H13 In the column (H11), in the row where "1st Medication" is written, write in the name of the first medication or hormone the subject used to help her become pregnant, even if this medication did not work. In the next column (H12), record the age when the subject started to use this medication/hormone. If the subject gives the answer in terms of the year she started, write that year to the left of the boxes where the age is to be recorded. In the next column (H13), record the total length of time in weeks, months, or years, that the subject took this medication/hormone. Do not include in this total periods of time when the subject was not taking the medication. Remember to circle the appropriate time frame the subject was referring to.

If the subject took more than one medication/hormone to help her become pregnant, these should be recorded in chronological order in the subsequent rows of the table, answering H11-H13 for each one. If the subject took a certain medication on more than one occasion with a lapse of time in between, then record this medication a second time as a separate entry.

H14 We are interested in whether the subject ever sought medical treatment from a doctor, clinic, or hospital, because of difficulty maintaining a pregnancy. If "yes," circle code "1" and continue with H15. If "no" or "don't know," circle the appropriate code and skip to Box H-4. If the subject had difficulty maintaining a pregnancy, but never sought medical treatment because of it, then the answer would be "no."

H15 Record the age when the subject first went to a doctor, clinic, or hospital for medical care because of difficulty maintaining a pregnancy. If the answer is given in terms of a calendar year, write the year to the left of the boxes where age is to be recorded.

H16 We are interested in whether a doctor was able to diagnose why the subject was having difficulty maintaining a pregnancy. If "yes," continue with Question H17. If "no" or "don't know," skip to Question H18.

H17 Probe for the nature of the subject's problem in maintaining a pregnancy, and circle the codes for all responses that apply. If the problem is other than, or in addition to, the 6 problems

listed, circle the code for “other” and write in the problem specified by the subject. If the subject does not know the reason for the difficulty, circle code “8” for "don't know.”

H18 If the subject ever took any medication/hormone to help her maintain a pregnancy, code "yes" and continue with H19-H21. If the answer is "no" or "don't know,” circle the appropriate code and skip to Box H-4.

H19-H21 These questions are about medications/hormones the subject took to maintain a pregnancy. They are to be answered in the same manner as H11-H13.

BOX H-4 Check the subject’s response to Question H1 on page H-1. If the subject was never pregnant or does not know if she ever was, skip to H24. If the answer was "yes,” that the subject was ever pregnant, continue with H22.

H22 If the subject ever took a medication to suppress lactation, code "yes" and continue with H23. If "no" or "don't know,” circle the appropriate code and skip to H24.

H23 Write the name of the medication that the subject took to suppress lactation. If the subject does not remember the name, circle code “8” for "don't know.”

H24 We are interested in whether a doctor diagnosed the subject as having endometriosis. If "yes,” continue with H25. If "no" or "don't know,” skip to Section J.

H25 Record the subject’s age when she was first diagnosed with endometriosis by a doctor. If the answer is given in terms of a calendar year, then write the year to the left of the boxes for age.

H26 Read each of the treatments for endometriosis listed in the answer category to the subject. Pause between each, to allow the subject to respond "yes" or "no" individually to each one. If she had been prescribed a medication to treat endometriosis, have her specify the name of the medication, and write it verbatim in the space provided.

SECTION J. HORMONE MEDICATION HISTORY

This section deals with the use of female hormone medications, specifically estrogen and progesterone.

BOX J-1 This section is to be completed for female subjects only. If the subject is a male, skip to Section K.

Lead-in Sentence: Read to the subject as written.

J1 Estrogen may be given for the relief of menopause or for other reasons such as irregular periods or bone loss. Record whether the subject ever took estrogen for any reason. If "yes," continue with J2. If "no" or "don't know," skip to Section L.

J2 We are interested in the form of estrogen the subject took. Read each category/estrogen form listed to the subject. Pause after each category read to allow the subject to give a response as to whether she ever used that particular form, and circle the appropriate code for "yes," "no," or "don't know." If the subject was given a form of estrogen other than, or in addition to, those specifically named in a-e, code "yes" for "other form" and write verbatim what that form was.

BOX J-2 Check the response to J2a. If estrogen was taken in the form of pills, continue with J3-J8. If not taken as pills, skip to BOX J-4.

ESTROGEN PILLS

J3-J8 This chart is used to record information specifically about the subject's use of estrogen pills. Complete one line of the chart for each episode of pill usage.

J3 Record the age the subject began taking a particular estrogen pill. Probe to get the most accurate response.

J4 Write in the complete name of the estrogen pill taken verbatim. It may be necessary to probe for the dose of the pill.

J5 Code the main reason why the subject took a particular pill. Read the possible responses to the subject so that she can choose the most appropriate reason. If the subject states more than one reason for taking the estrogen, probe to find out the primary reason. Circle only one code for the primary reason.

J6 Record the age the subject stopped taking a particular estrogen pill. Probe to get the most accurate response. If the subject is currently taking this estrogen pill, circle code "00" and do not record an age.

J7 We are interested in whether the subject was also taking a progesterone pill during the time she was taking the estrogen pill. If "yes," continue with J8.

If "no" or "don't know," go back to J3 and record information for the next estrogen pill taken, if applicable. If this was the only or last estrogen pill taken by the subject, then go to Box J-3.

J8 Write the name of the progesterone pill verbatim that the subject took with the estrogen pill. If necessary, probe for the dose of the progesterone taken.

ESTROGEN SHOTS

BOX J-3 If the subject reported in Question J2b that she has used estrogen in the form of a shot or injection, then ask J9-J11. If not, skip to Section L.

J9-J11 This chart is used to record information specifically about estrogen shots the subject received. Complete one line of the chart for each episode of shots received.

J9 Record the subject's age when she first started receiving estrogen shots. If the answer is given in terms of a year, write the year to the left of the boxes for age. Probe if necessary to get the most accurate answer.

J10 Record the subject's age when she stopped receiving estrogen shots. If the answer is given in terms of a year, write the year to the left of the boxes for age. If the subject is currently receiving estrogen shots, circle code "00" and do not record an age. If after probing the subject does not know, circle code "98."

J11 We are interested in the frequency with which the estrogen shots were administered between the ages given in J9 and J10. Record the number of times the shots were received in a day, week, month, or year, whichever is reported by the subject. Be sure to circle the code for the

time period in which the answer is given. If, after probing for the answer the subject does not know, circle code "988."

Skip to Section L for all female subjects.

SECTION K. MALE FERTILITY

This section of the questionnaire is about issues related to male fertility.

BOX K-1 These questions are to be completed for male subjects only. If the subject is a female, skip to Section L.

Lead-in Statement: Read to the subject verbatim.

K1 Record how many live-born children for whom the subject is the natural father. Do not count in this number stepchildren, foster children, or adopted children who were raised by the subject. If the subject did not ever father a live-born child, circle code "00" for "none." If the subject does not know, do not probe since this may be a sensitive issue, and circle code "98" for "don't know."

K2 We are interested in whether the subject ever attempted unsuccessfully to get his wife/partner pregnant, for a period of one year or longer. This is not to include attempts made during the past 12 months. It does not matter whether difficulty with getting pregnant was a result of a male (the subject's) or female problem with fertility.

For example, if the subject and his wife/partner had been trying to achieve pregnancy for 15 months, and 12 of those months were before one year ago and the other 3 months were during the past year, code "yes." If they had tried to achieve pregnancy for 8 months before one year ago, and for another 7 months during the past year, also code "no."

If the answer is "yes," continue with K3. If the answer is "no" or "don't know," skip to K5.

K3 If the subject and his wife/partner had difficulty achieving pregnancy as determined in K2, we want to know if he (the subject) or she ever went to a doctor, clinic, or hospital about this problem. If "yes," continue with K4. If "no" or "don't know," skip to K5.

K4 Probe to find the main reason for the infertility problem (even if pregnancy was achieved eventually). If the problem was only with the wife/partner, circle code “1.” If the problem was only with the subject, circle code “2.” If both the subject and his wife/partner were diagnosed with a fertility problem, circle code “3.” If the subject indicates the problem was other than these, circle code “6” and specify what the reason is/was in the space provided. If the subject does not know what the nature of the problem is/was, circle code “8” for "don't know.”

K5 A vasectomy is a surgical procedure in which the vas deferens of the male is completely or partially removed, making him sterile/infertile. If the subject had a vasectomy, code "yes" and continue with K6. If "no" or "don't know,” skip to Section L.

K6 Record the subject’s age when he had a vasectomy. If the answer is given in terms of the year it was done, write the year to the left of the boxes for age.

SECTION L. FAMILY HISTORY OF DISEASE

In this section of the questionnaire, questions are being asked about the subject’s relatives and their medical history.

BOX
L-1 These instructions require you to turn back to A7 to determine the subject’s marital status. If the subject was ever married (A7=1, 3, 4, 5), read the lead-in sentence just below this box to the subject and begin with L1. If the subject was never married or if it is unknown (A7=2, 6, 8), place a check in the small box and skip to the lead-in statement following L3.

Lead-in Sentence: Read to the subject as written, in accordance with the instructions in Box L-1.

L1 Record the year that the subject’s spouse (husband/wife) was born.

L2 Record whether the subject’s spouse is still alive. If the spouse is deceased, circle code “2” for “no” and continue with L3. If the spouse is still living or if it is unknown whether the

spouse is alive or deceased, circle the appropriate code and skip to the lead-in statement following L3.

L3 Record the age of the subject's spouse at the time of his/her death. If only the year of death is known, write the year to the left of the boxes for age.

Lead-in Statement: Read to the subject as written. This statement is important to let the subject know that the following questions will be referring <u>only</u> to relatives who are related to the subject by blood. This does not include in-laws or adopted children. We are only interested in those relatives who are genetically or biologically related to the subject.

L4 We are interested in whether the subject feels capable of answering questions regarding the health of his/her blood relatives (i.e., biological mother and father, full brothers or sisters, or half-brothers or sisters). If "yes," continue with L5. If "no," skip to Section M.

If the subject feels as if he/she can answer health related questions for some, but not all, of his/her blood relatives, write a note in the margin to explain this, then continue with L5.

L5 Record whether the subject was born as a member of a multiple birth. This would include twins or triplets and all other multiple birth combinations (quadruplets, quintuplets, etc.). If "yes," continue with L6. If "no" or "don't know," skip to L7.

L6 We are interested in whether the subject was a member of an <u>identical</u> or <u>fraternal</u> (non-identical) set of twins or triplets. Identical twins/triplets have identical genetic makeups. Fraternal twins/triplets are no more alike genetically than ordinary brothers and sisters. Circle the appropriate code according to the subject's response.

L7 Record the year that the subject's biological mother was born. Probe if necessary to get the most accurate response. Write "est." next to the year if it is only an estimate. If the subject does not know the year of birth, then write "don't know" in space to the right of the boxes.

L8 Record whether the subject's biological mother is still alive. If she is deceased, circle code "2" for "no" and continue with L9. If she is still living or if it is unknown whether the subject's mother is alive or deceased, circle the appropriate code and skip to L10.

L9 Record the age of the subject's biological mother at the time of her death. Remember to right-justify the number (i.e., 89 years old is coded as 089). If only the year of death is known, write the year to the left of the boxes for age.

L10 Record the year that the subject's biological father was born. Probe if necessary to get the most accurate response. Write "est." next to the year if it is only an estimate. If the subject does not know the year of birth, then write "don't know" in space to the right of the boxes.

L11 Record whether the subject's biological father is still alive. If he is deceased, circle code "2" for "no" and continue with L12. If he is still living or if it is unknown whether the subject's father is alive or deceased, circle the appropriate code and skip to L13.

L12 Record the age of the subject's biological father at the time of his death. Remember to right-justify the number (i.e., 89 years old is coded as 089). If only the year of death is known, write the year to the left of the boxes for age.

L13 We are interested in the number of each type of blood relative listed (a-f) that the subject has, whether they are alive or deceased. Read the question for each type of relative in the order listed, and record a response before moving on to the next one. Write how many of a particular relative that the subject has in the boxes provided, being sure to right-justify. If the subject has none of a particular type of relative or does not know if he/she does, circle the appropriate code and go to the next relative listed.

Note that siblings of the subject's are <u>full</u> sisters or brothers if the subject has both the same biological mother <u>and</u> biological father as his/her sister(s) or brother(s). The subject's siblings are <u>half</u> sisters or brothers if the subject has either the same biological mother <u>or</u> biological father as his/her sister(s) or brother(s), but not both sets of parents are the same.

After recording the appropriate response for item f, go to Box L-2.

Box L-2 Look back at the responses given by the subject in L13 for each type of relative listed in a-f. For each type of relative that the subject has one or more of, as indicated by having a number written in the boxes, go to L14. In L14, record the full names of each of the relatives in a

particular relationship category on separate rows, and circle the appropriate code for that person's relationship to the subject. Then ask L15-L18, as appropriate, for each of the names listed.

Helpful Hint: It is best to complete L14-L18 for a single relationship category before going on to the next one, in order to keep track of where you are and to be certain that no relative is missed by mistake.

L14-L18 Same table appears on pages L-4 to L-7, providing room for listing 20 relatives.

L14 Record the full name of each of the subject's relatives from L13 (a-f) in a separate row. Also, circle the code corresponding to each particular person's relationship to the subject. Refer to the guidelines for Box L-2 for additional help in answering this question.

Only if the relative is the subject's half sister or half brother, L14=3 or 4, continue with L15. Otherwise, skip L15 and go to L16.

L15 For half brothers and half sisters, we are interested in whether the subject and his half brother/sister have the same biological mother <u>or</u> the same biological father in common. Circle the appropriate response and continue with L16.

L16 Record the year in which the relative named in L14 was born. Probe if necessary to get the most accurate response.

L17 Record whether the relative named in L14 is alive or deceased. If the relative is no longer living, continue with L18. If the relative is alive, or if the relative's vital status is not known, skip L18 and go to the next relative listed under L14. If there are no other relatives in the category you are currently inquiring about, go to the next relationship category listed under L13 and complete L14-18 in the same manner as described in the instructions for Box L-2 and L14-18.

L18 Record the age of the relative named in L14 when he/she died. Be sure to right-justify the answer when writing it in the boxes. If the answer is given as a calendar year, write the year in the space to the left of the boxes for age. Continue with the next relative listed under L14.

If there are no other relatives in the category you are currently inquiring about, go to the next relationship category listed under L13 and complete L14-18 in the same manner as described in the instructions for Box L-2 and L14-18.

BOX L-3 L19 is to be asked about the subject's blood relatives including his/her mother, father, siblings, and children, and also his/her spouse. For each "yes" response in L19, you are to go to the grid specifically for the condition asked about. In the appropriate grid, the name of each relative having that condition is to be entered. Then, the other questions in the grid are to be asked for each relative named.

Lead-in Statement: Show the subject Show Card L-1 and read the introduction to the next set of questions to the subject verbatim. The introduction will make the subject aware that the next set of questions are about specific medical conditions (listed on Show Card L-1) that his/her relatives may have had or currently have.

L19 We are interested in whether any of the subject's blood relatives, including his/her biological mother and/or biological father, and also his/her spouse (if the subject was ever married), had ever been diagnosed by a doctor as having any of the medical conditions listed as items a-g. Obtain a response for each condition listed. If one or more of the relatives being considered was ever diagnosed with a particular condition by a doctor, the response should be coded "yes."

GRID A. GALLBLADDER CANCER

L20 In separate rows, write the first name of each of the subject's blood relatives and spouse who has had gallbladder cancer. Also, write what each person's relationship is to the subject. Probe by asking the subject if there is any other relative who has had gallbladder cancer, before going on, to be sure that no relative is missed by mistake. Then, ask L21-L23 for each relative named.

L21 Record the relative's age when he/she was first diagnosed with gallbladder cancer by a physician. Probe to get the most accurate answer possible.

L22 Circle the appropriate code for whether the named relative had a cholecystectomy (i.e. had his/her gallbladder surgically removed).

L23 Record the name and location (province) of the hospital where the relative was first diagnosed with gallbladder cancer. Then ask L21-L23 for the next relative listed under L20. If there

are no further relatives listed, go to the grid for the next medical condition under L19 for which the subject has at least one relative with the condition.

GRID B. EXTRAHEPATIC BILE DUCT CANCER

L24 In separate rows, write the first name of each of the subject's blood relatives and spouse who has had extrahepatic bile duct cancer. Also, write what each person's relationship is to the subject. Probe by asking the subject if there is any other relative who has had extrahepatic bile duct cancer, before going on, to be sure that no relative is missed by mistake. Then, ask L25 and L26 for each relative named.

L25 Record the relative's age when he/she was first diagnosed with bile duct cancer by a physician. Probe to get the most accurate answer possible.

L26 Record the name and location (province) of the hospital where the relative was first diagnosed with bile duct cancer. Then ask L25 and L26 for the next relative listed under L24. If there are no further relatives listed, go to the grid for the next medical condition under L19 for which the subject has at least one relative with the condition.

GRID C. LIVER CANCER

L27 In separate rows, write the first name of each of the subject's blood relatives and spouse who has had liver cancer. Also, write what each person's relationship is to the subject. Probe by asking the subject if there is any other relative who has had liver cancer, before going on, to be sure that no relative is missed by mistake. Then, ask L28 and L29 for each relative named.

L28 Record the relative's age when he/she was first diagnosed with liver cancer by a physician. Probe to get the most accurate answer possible.

L29 Record the name and location (province) of the hospital where the relative was first diagnosed with bile duct cancer. Then ask L28 and L29 for the next relative listed under L27. If there are no further relatives listed, go to the grid for the next medical condition under L19 for which the subject has at least one relative with the condition.

GRID D. OTHER CANCER

L30 In separate rows, write the first name of each of the subject's blood relatives and spouse who has had any cancer other than gallbladder cancer, extrahepatic bile duct cancer, or liver cancer. Also, write what each person's relationship is to the subject. Before going on, probe to be sure that no other blood relative, nor the subject's spouse, is missed by mistake. Then, ask L31- L33 for each relative named.

If the same relative has had more than one type of cancer, write his/her name on separate rows for each different type of cancer. Then, ask L31-L33 for each type of cancer this person has had.

L31 Record the type of cancer the relative had or the location in the body where the cancer started. Write the information verbatim on the lines provided.

L32 Record the relative's age when he/she was first diagnosed by a doctor as having the type of cancer specified in L31. Probe to get the most accurate answer possible.

L33 Record the name and location (province) of the hospital where the relative was first diagnosed with the type of cancer specified in L31. Then ask L31-L33 for the next relative listed under L30. If there are no further relatives listed, go to the grid for the next medical condition under L19 for which the subject has at least one relative with the condition.

GRID E. GALLSTONES

L34 In separate rows, write the first name of each of the subject's blood relatives and spouse who has had gallstones. Also, write what each person's relationship is to the subject. Probe by asking the subject if there is any other relative who has had gallstones, before going on, to be sure that no relative is missed by mistake. Then, ask L35-L37 for each relative named.

L35 Record the relative's age when he/she was first diagnosed with gallstones by a doctor. Probe to get the most accurate answer possible.

L36 We are interested in how the relative's gallstones were treated. Do not read the possible treatments listed to the subject. Circle the code which most accurately describes the type of treatment used for the gallstones. If the treatment was other than surgical removal of the gallbladder, medications, or a specific diet, circle code "4" and ask the respondent to specify the type of treatment the relative received. Write the specified treatment verbatim on the line provided.

L37 Record the name and location (province) of the hospital where the relative was first diagnosed with gallstones. Then ask L35-L37 for the next relative listed under L34. If there are no further relatives listed, go to the grid for the next medical condition under L19 for which the subject has at least one relative with the condition.

GRID F. HEPATITIS

L38 In separate rows, write the first name of each of the subject's blood relatives and spouse who has had hepatitis. Also, write what each person's relationship is to the subject. Probe by asking the subject if there is any other relative who has had hepatitis, before going on, to be sure that no relative is missed by mistake. Then, ask L39-L41 for each relative named.

L39 Record the relative's age when he/she was first diagnosed with hepatitis by a doctor. Probe to get the most accurate answer possible.

L40 We are interested in the type of hepatitis the relative had/has. Do not read the possible treatments listed to the subject. Probe to obtain the most accurate response and circle the appropriate code(s). There may be more than one answer to this question. For example, the relative may have had an acute case of Hepatitis A. Therefore, codes "1" and "3" would be circled.

As an aide for probing, the following definitions are provided:

- Hepatitis A is also known as infectious hepatitis. It is usually an acute, benign condition caused by a virus that does not persist in the blood serum and is transmitted through food and water that has been contaminated with fecal matter.
- Hepatitis B is also known as serum hepatitis because it is caused by a virus that tends to persist in the blood serum. It is transmitted by contact with infected blood.

- An acute disease is characterized by severity, sudden onset, sharp rise, and short course. Whereas, a chronic disease is marked by a long duration, frequent recurrence, and slowly progressing seriousness.

L41 Record the name and location (province) of the hospital where the relative was first diagnosed with hepatitis. Then ask L39-L41 for the next relative listed under L38. If there are no further relatives listed, go to the grid for the next medical condition under L19 for which the subject has at least one relative with the condition.

GRID G. DIABETES

L42 In separate rows, write the first name of each of the subject's blood relatives and spouse who has had diabetes. Also, write what each person's relationship is to the subject. Probe by asking the subject if there is any other relative who has had diabetes, before going on, to be sure that no blood relative, nor the subject's spouse, is missed by mistake. Then, ask L43-L45 for each relative named.

L43 Record the relative's age when he/she was first diagnosed with diabetes by a doctor. Probe to get the most accurate answer possible.

L44 We are interested in whether the type of diabetes the subject's relative had was insulin dependent, also known as Type I or juvenile onset, or whether it was insulin independent, also known as Type II or adult onset. Circle the appropriate code for the subject's response.

L45 Record the name and location (province) of the hospital where the relative was first diagnosed with diabetes by a physician. Ask L43-L45 for each relative listed under L42. Then, continue with L46.

L46 We are interested in whether there are any other illnesses or medical conditions which occur frequently in the subject's family among his/her blood relatives, excluding those conditions already asked about in L19. Circle the appropriate code for the response given. If "yes," continue with L47. If "no" or "don't know," skip to Box L-4.

L47 Write the name of any medical conditions or illnesses which tend to run in the subject's family among his/her blood relatives. Write these verbatim, using a separate line for each condition mentioned. Do not include those conditions already asked about in L19.

BOX
L-4 The interviewer is to determine who answered most of the questions in this section of the questionnaire, Section L-Family History. Circle the appropriate code for whether the subject or a proxy answered the most questions, or both the subject and a proxy answered nearly an equal number of questions.

SECTION M. PHYSICAL DEVELOPMENT AND ACTIVITY

This section of the questionnaire includes questions about height and weight at various ages, and about physical activities.

Lead-in Sentence: Read to the subject as written.

M1 Record the subject's height as an adult in centimeters in the boxes provided. Circle "998" if the response is "don't know."

M2 In this question, we are asking the subject to compare his/her height at particular ages as a child, to other children of the same age and sex. With these questions we are attempting to follow the subject's height throughout puberty.

There are 3 age categories (a-c) asked about, each requiring a response. Read the question the first time, substituting in the first age category (a). Code the answer given by the subject. Read the question 2 more times, each time substituting in the next age category listed (b, then c). Code the appropriate response for each. Probe if necessary to get the most accurate answers. If the subject is unable to remember his/her height for any of the categories asked about, after probing code "don't know."

M3 Record in kilograms what the subject's usual weight has been during his/her entire adult life. This may be difficult for the subject to answer if there have been large fluctuations in his/her adult weight. Therefore, it may be necessary to probe to get the most accurate response. By "adult," we mean 18 years of age and older. If the subject is female, weight during pregnancy should be excluded. Circle "998" if the response is "don't know."

M4 Record the subject's current weight in kilograms. Circle "998" if the response is "don't know."

M5 Record, in kilograms, what the subject's usual weight was 5 years ago. If the subject is female, weight during pregnancy should be excluded. Circle "998" if the response is "don't know."

M6-M8 These questions are about the subject's size and weight at different ages throughout his/her life. Ask M6-M8 for each of the applicable age groups, and for the year 1990, listed at the top of the columns. Note that for the first age group, "8-9" years old, only M6 is asked. If the subject is 34 years old, for example, ask these questions for age categories "8-9," "12-14," "20-29," "30-39," and for "1990." Do not ask these questions for age categories "40-49," "50-59," and "60-69" since the subject has not reached those ages. Circle code "+" for "not applicable" in M6 for each of the inapplicable age groups.

M6 This question should be read, substituting in the appropriate age category or the year 1990. Read the possible responses to the subject each time the question is read so he/she can choose the most appropriate answer, then circle the corresponding code. After completing M6 for ages "8-9," go to the next age category in M6. For all other age categories in M6, ask M7 and M8 before going on to the next age category in M6.

M7 Record the subject's usual weight in kilograms for the particular age category or the year 1990. Probe, if necessary, to get the best answer. If the subject is female, exclude weight during pregnancy. Circle "998" if the response is "don't know." Continue with M8 for the same category.

M8 We are interested in the subject's trouser waist size for the particular age category or for the year 1990. Record the answer in centimeters. Probe, if necessary, to get the best answer. If the subject is female, exclude waist size during pregnancy. Continue with the next category in M6 if any remain, or go to M9.

M9 Record the subject’s highest weight as an adult in kilograms. Read the statement in parentheses to female subjects only, to make them aware that times when pregnant, nursing, or 6 months after pregnancy should be excluded.

M10 Record the subject’s age when he/she first reached his/her highest adult weight. If the answer is given as a year, write the year to the left of the boxes for age.

M11 Record the length of time, in terms of months or years, the subject was at his/her highest weight. Also, circle the appropriate code for years or months.

BOX M-1 If anyone other than the subject is being interviewed (i.e., a proxy), place a check mark in the box where indicated, and skip to Section N. If the subject is being interviewed, continue with M12.

M12 We are interested in the region of the subject’s body where he/she typically puts on weight. That is, the region of the body where the subject’s weight gain can usually be first observed. The answer categories are not to be read to the subject, therefore, it may be necessary to probe for this answer. Circle the code for the most accurate response.

If the subject reports a combination of body regions, probe for the main region where he/she tends to gain weight. If the subject claims he/she does not gain weight, circle code “0” for “don’t gain weight.” If weight is typically put on in another body region than the ones specifically listed, circle the code for “other” and write in the region the subject tells you verbatim. If, when the subject gains weight there is no main region in which the weight gain can be observed, circle the code for “equally all over.”

M13 Record the number or times since he/she was 20 years old, that the subject has lost at least 7 kilograms and then later gained at least 7 kilograms back. Read the phrase in parentheses to female subjects only, followed by the rest of the question. For male subjects, read only the part of the question after the parentheses. Female subjects are not to include in their answer weight that was lost after a pregnancy and then was gained back later.

If the subject gives a range of times as the answer, such as “2 to 6 times,” probe for a single number of times to record. If the subject insists on a range of times, write the range in the

space to the left of the listed responses. If the subject has never lost and regained at least 7 kilograms since the age of 20, circle code “00” for “none.”

M14 We are interested in how frequently the subject participated in vigorous physical activities, before 5 years ago. Recent changes in physical activity are not to be considered. The possible answers are not to be read to the subject. Use SHOW CARD C to help the subject determine what types of activities are considered vigorous. Circle the code for the most appropriate response.

M15 We are interested in how frequently the subject participated in moderate physical activities, before 5 years ago. Recent changes in physical activity are not to be considered. The possible answers are not to be read to the subject. Use SHOW CARD D to help the subject determine what types of activities are considered moderate. Circle the code for the most appropriate response.

M16 We are interested in how frequently the subject, before 5 years ago, climbed 2 or more flights of stairs without stopping. Recent changes in this activity are not to be considered. A flight of stairs consists of at least 10 steps. The possible answers are not to be read to the subject. Circle the code for the most appropriate response.

M17 We are interested in the method of travel that the subject used most often to go between home and work, before 5 years ago. Read the methods listed to the subject so that he/she may choose the most appropriate response, then circle the corresponding code. If a method of travel other than walking, bicycling, or riding a moped was used by the subject most often, circle code “6” and write the method specified by the subject verbatim, on the line provided.

If the response to M17 was “use another method of travel” or “don’t know,” skip to Section N. Otherwise, continue with M18.

M18 Record the number of kilometers the subject traveled round-trip between home and work, before the past 5 years, using the method of transportation specified in M17.

M19 Record the number of times the subject traveled round-trip between home and work, before 5 years ago, in a day, a week, or a month. Record the response using the time frame reported

by the subject. Remember to circle the code corresponding to the time frame in which the answer was given.

SECTION N. OCCUPATIONAL HISTORY

In this section, you will obtain information about the subject's three longest worked occupations.

Lead-in Sentence: Read to the subject as written.

N1 Record whether the subject has ever been employed or had a job for at least six months. We are interested in all jobs, full- or part-time, paid or unpaid, including self-employment, that were held for six months or more. If the subject worked on the family farm without being paid, this would still be considered a job. If the subject had no other job besides "housewife," let the subject decide whether to consider this as a job.

If "yes," continue with Questions N2-N7. If "no" or "don't know," skip to Section P.

N2-N7 In these questions we ask the subject for detailed information about the three different types of jobs (occupations) held by him/her for the longest periods of time. Each of the three rows of the chart is for a separate occupation, beginning with the one that was held the longest, then the next longest, and then the next longest. Each column is a separate question (N2-N7) that is to be asked about each type of job listed. Ask each question about a particular job before going on to the next one.

Note that we are interested in different occupations or types of work held by the subject. If the subject worked for the same employer but had different job titles and duties, these would be considered different occupations. Also, if the subject had the same or similar job title and performed the same or similar duties at more than one company, these would not be considered different occupations.

If a job title and information are recorded out of sequence, for example, the subject forgot to mention a particular job, make a note of this in the left margin. Do not try to erase or correct a mistake of this nature.

This section may require considerable probing. If a subject is having difficulty remembering occupations or when he/she held a particular job, it may be helpful to probe by having the subject relate the jobs to events in his/her life. Examples of events may be marriages, births, deaths, wars, and moves from one location to another.

N2 We are interested in the subject's job title, i.e., what the job was called, for the particular occupation being asked about (the longest, next longest, or next longest). Write the subject's response verbatim in the appropriate space.

If the subject held a job which did not have a specific job title, ask for a description of the type of work. Probe to obtain a job title which reflects the type of work performed as accurately as possible. Note that a one-word entry is almost never adequate. For example, a "clerk" can be a shipping clerk, file clerk, stock clerk, or sales clerk.

The following are examples of adequate and inadequate job title entries.

Inadequate	Adequate
Adjuster	Claim adjuster, machine adjuster, complaint adjuster, insurance adjuster.
Data Processing	Computer programmer, keypunch operator, computer operator, coding clerk.
Engineer	Civil engineer, mechanical engineer, aeronautical engineer.
Equipment Operator	Road grade operator, bulldozer operator, trench operator, city bus driver.
Factory Worker	Electric motor assembler, weaver, loom fixer, knitter, stitcher, punch press operator, spray painter, riveter.
Farmer	Farm owner, fruit picker, cotton chopper, ranch hand.
Foreman/lady	Specify the craft or activity involved, as forelady carpenter, forelady truck driver.
Mechanic	Auto mechanic, dental mechanic, radio mechanic, airplane mechanic, office machine mechanic.
Officer Worker	Typist, secretary, receptionist, comptometer operator, file clerk, bookkeeper, physician's attendant.

Specialist	If the word specialist is reported as part of a job title, be sure that in N3 you get a good description of the actual duties. For example, for a "transportation specialist," the actual duties might be any of the following: "Gives cost estimates of trips," "schedules trains," or "does economic analysis of transportation industry."
Supervisor	Typing supervisor, chief bookkeeper, steward, kitchen supervisor, buyer, route foreman.
Teacher	For elementary school teachers who teach many subjects, an entry such as "4th grade teacher" is satisfactory. For those who specialize, both the subject and grade level should be reported, such as "11th grade physics teacher." College teachers should be recorded by subject and title, such as "English instructor" or "history professor."

N3 We are interested in the subject's usual activities and duties when he/she had a particular job title. Probe for specifics. An answer such as "do office work," is not adequate since it is not specific enough about the duties performed. However, it would be acceptable if the answer was "typing, photocopying, scheduling appointments, and sorting mail." If "ordering supplies" was done only occasionally, because it was someone else's job, then this should not be included in the list of usual activities. Write the subject's description of his/her duties verbatim.

N4 In this question, consider the company where the subject worked most often at the particular type of occupation (job title) performing the job duties specified. We are interested in what the subject's company, or specific division of the company (if the company was involved in different types of activities), specialized in. If the subject regularly spent 75% of his/her time in one division of a company and 25% in a different division performing similar duties, record the specialization of the division where the subject was during the <u>bulk</u> of his/her time. Or, if the subject worked the same occupation for 10 years at one company and for 3 years at a different company, the subject should give a response for the company where he/she worked this occupation the longest time.

We want to know what industry the company or division of the company is involved in, and more specifically, what they make or what they do. Probe to get a complete answer. One-word descriptions are not adequate. Note that the distinction between wholesale, retail, and manufacturing places is very important in classifying industries. These are further discussed below. A response of "manufacturing firm" is <u>not</u> an adequate response. You must probe to

find out what was manufactured. A response of "business" is not adequate. You must probe to find out what kind of business. If a company is a sales organization, probe to find out if it is retail or wholesale. If a company is a “mine,” probe to find out what type of mine. Even when it seems self-explanatory what the company did, going through this process will ensure that you get a response with enough detail.

The following are examples of adequate and inadequate entries for what the subject’s company specialized in.

Inadequate	Adequate
Mine	Coal mine, iron mine, clay mine.
Lumber Company	Logging company, lumber manufacturing company, lumber wholesale outlet, lumber retail outlet.
Radio Company	Radio broadcasting company, radio repair shop, radio manufacturing company, radio wholesale supplier, radio retail store.
School	Elementary school, secondary school, college, university.
Water Company	Water utility, water bottling plant, bottled water wholesale outlet.
Furniture Company	Furniture manufacturer, wholesale furniture store, retail furniture store, furniture refinishing company.
Oil Company	Fuel oil retail sales, oil refinery, oil drilling, oil extraction, out exploration service.
Movie (or motion picture) Company	Movie theater, movie distribution company, motion picture production, motion picture studio.

Use the following additional items as a guideline for recording the responses to N4.

- **Avoid Use of the Word "Company" -** Do not use the word "company" when writing down the response. It does not give useful information. If the subject reports that she works for a metal furniture company, ask "Do they manufacture or do they just sell it"? If they just sell it, ask "Do they sell to other stores (which would be wholesale) or to individuals (which would be retail)”? In such a case the possible replies would be "furniture manufacturer,” "furniture wholesaler,” or "furniture retailer.” Note that where possible, you should specify for furniture manufacturers the major material used -- wood, metal, plastic, etc., but for the selling operation, it is not necessary, since furniture wholesalers and retailers very often sell various types.

- **Multiple Activity Business -** Some firms carry on more than one kind of business or industrial activity. If the activities are carried on at the same location, describe only the major activity of the establishment. For example, employees in a retail salesroom, located at the factory of a company primarily engaged in the manufacturing of women's clothing should be reported as working in "Women's Clothing Manufacturing."

    - If the different activities are carried on at separate locations, describe the activity where the subject works. For example, if the subject worked at a coal mine owned by a large steel manufacturer, report it as "coal mine" rather than as a large steel manufacturer. Report a separate paint factory of a large chemical manufacturer as "paint manufacturing" rather than a large chemical manufacturer.

    - Also, record the activity of the parent organization for research laboratories, warehouses, repair shops, and storage garages, when these kinds of establishments exist primarily to serve their own parent organizations. For example, if a retail department store has a separate warehouse for its own use, the entry for the warehouse employees should be "warehouse for a retail department store."

- **Distinguish Among Manufacturing, Wholesale, Retail and Service -** It is essential to distinguish among manufacturing, wholesale, retail, and service companies. Even though a manufacturing plant sells its products in large lots to other manufacturers, wholesalers, or retailers, it should be reported as a manufacturing company. Use the following as a guide:

    - A wholesale establishment buys, rather than makes, products in large quantities for resale to retailers, industrial users, or to other wholesalers.

    - A retailer sells primarily to individual consumers or users but seldom makes products.

    - Establishments which render services to individuals and to organizations such as hotels, laundries, cleaning and dying shops, advertising agencies, and automobile repair shops are engaged in providing services. Report these as retailers but show the type of services provided, for example, TV and radio repair.

- **Manufacturers' Sales Offices -** Record a separate sales office set up by a manufacturing firm to sell to other business organizations and located away from the factory or headquarters of the firm as "(product) manufacturer's sales office." For example, if a shoe manufacturer located in one city has a sales office in another city, the correct entry for workers in the sales office would be "shoe manufacturer's sales office."

- **Government Organization -** Usually the name of the government agency is adequate, for example, Census Bureau, City Fire Department.

    - If the activity of the government agency is absolutely clear, the name of the agency is sufficient. However, sometimes the name of government agencies are

not fully descriptive of their business or activity. A correct entry for a County Highway Commission might be one or any combination of the following: "County road building," "county road repair," or "county contracting for road building (or repair)." For State Liquor Control Board, the correct entry might be "State licensing of liquor sales," or "State liquor retailer."

- If the business or main activity of a government employer is not clear, you should ask in what part of the organization the subject works and then report that activity. For example, for a City Department of Public Works, a correct entry might be one of the following: "City street repair," "city garbage collection," "city sewage disposal," or "city water supply."

N5 Record the year that the subject started working in the particular occupation/job title. This year may or may not be the year that the subject started working with the employer discussed in N4. If the answer is given as the subject's age, write the age to the left of the boxes for year.

N6 Record the total number of years the subject worked at the particular occupation/job title. If the subject worked at this occupation in more than one company, the answer should include the number of years at each of the different companies. Exclude periods of time when the subject was not working at this type of job. If the subject can only give his/her age when this type of work was stopped, record the age to the left of the boxes for year. In this case, probe to find out if there were any periods of time when the subject was not at this occupation, and make a note in the margin.

If the employment was seasonal, find out how many months of each year that the subject worked in this position and write a note stating this.

If the subject asks you to figure out the answer to N6, be sure to verify it. It is much better to encourage the subject to come up with the answer. This frequently makes them reconsider and possibly revise the year they gave you for N5.

N7 Record the subject's own assessment of whether the work required for this particular occupation was light, moderately strenuous, or very strenuous. The interviewer may probe to get a response, but should not comment or offer an opinion as to what level of physical activity a particular type of work may be considered.

SECTION P. PHYSICAL MEASUREMENTS

Measurement of each subject's standing height, weight, waist, and hips will be obtained. The equipment that is required to perform these measurements is minimal. Standing height and weight are measured using a balance beam scale that is equipped with a height measuring rod. The waist and hip measures are obtained using a standard cloth or paper tape measure, and a non-permanent marking pen. Below are the specific procedures for performing each of these physical measurements.

P1 Standing Height

- Have the subject stand erect on the platform of the balance beam scale with his/her back toward the height measuring rod.
- The subject's feet should be flat with his/her weight evenly distributed on both feet.
- The feet should be pointed slightly outward at a 60° angle with the heels placed together. If the subject's knees prevent him/her from placing his/her heels together, the feet should be separated so that the inside of the knees are in contact, but not overlapping.
- The subject's arms should hang freely by the sides of his/her trunk, with the palms of the hands facing the thighs.
- Have the subject inhale deeply, stand fully erect, and look straight outward without altering the position of the heels.
- The subject's head should be maintained in the Frankfort Horizontal Plane position while the horizontal bar of the measuring rod is positioned at a 90° angle to the measuring rod and placed snugly to the crown of the subject's head with sufficient pressure to compress the hair. Hair ornaments, buns, braids, etc. must be removed to obtain an accurate measurement.

  Note: the Frankfort Horizontal Plane is the position of the head when the upper margin of the ear openings and lower margin of the orbit of the eyes are horizontal.
- Read the subject's height in centimeters from the measuring rod to the nearest 0.1 cm, and record this value in the first set of boxes.
- Repeat this procedure a second time and record the value in the second set of boxes. If the difference between the two height measurements is >2.0 cm, the measurement must be taken a third time following the above procedure, and recorded in the third set of boxes.

P2 Abdominal (Waist) Circumference

This procedure may be carried out with one examiner and the assistance of the subject. If the subject is unable to assist, a second examiner will be necessary in order to obtain an accurate measurement.

- Have the subject in the standing position.
- Stand behind the subject and palpate the right hip area for the high point of the iliac crest. The subject's underclothing must be lowered slightly in order to palpate directly on the hip area.
- With a non-permanent marking pen, mark a horizontal line at the high point of the iliac crest and then cross the line to indicate the midaxillary line of the body.
- Stand on the subject's right side and place the start of the measuring tape on the cross mark, and have the subject place a finger on the tape to hold it in place.
- Place the measuring tape around the subject's trunk in a horizontal plane at the level marked on the right side of the trunk. This is most easily accomplished by walking around the subject with the measuring tape.
- Make sure that the tape is parallel to the floor around the entire circumference of the waist, and that it is snug, but does not compress the skin.
- With the zero end of the tape held under the measurement value, the measurement is made at minimal respiration, when viewing at eye level, to the nearest 0.1 cm. Record this value in centimeters in the first set of boxes.
- Repeat this procedure a second time and record the value in the second set of boxes. If the difference between the two waist measurements is >2.0 cm, the measurement must be taken a third time following the above procedure, and recorded in the third set of boxes.

P3 Buttocks (Hips) Circumference

This procedure may be carried out with one examiner and the assistance of the subject. If the subject is unable to assist, a second examiner will be necessary in order to obtain an accurate measurement.

- Have the subject stand erect with his/her feet together (or as close together as possible) and with his/her weight evenly distributed on both feet.
- Have the subject lower his/her underclothing to below the buttocks.
- Stand on the right side of the subject and, with a non-permanent marking pen, mark a horizontal line at the level of the maximum extension of the buttocks. Place the beginning of the measuring tape on the mark, and have the subject put a finger on the tape to hold it in place.

- Place the measuring tape around the subject's hips in a horizontal plane at the level of the line marked on the right side. This is most easily accomplished by walking around the subject with the measuring tape.

- Make sure that the tape is parallel to the floor around the entire circumference of the hips, and that it is snug, but does not compress the skin.

- With the zero end of the tape held under the measurement value, the measurement is made, when viewing at eye level, to the nearest 0.1 cm. Record this value in centimeters.

- Repeat this procedure a second time and record the value in the second set of boxes. If the difference between the two hip measurements is >2.0 cm, the measurement must be taken a third time following the above procedure and recorded in the third set of boxes.

P4 Weight

- Have the subject stand in the center of the balance beam scale platform, facing forward.

- Move the bottom weight across the notched bar to a position where the bar is nearly balanced. Move the top weight slowly across the bar to the position that completely balances the bar.

- Read the numbers where the weights rest on each bar and add these numbers together to obtain the weight measurement to the nearest 0.1 kg. Record this value in kilograms in the first set of boxes.

- Repeat this procedure a second time and record the value in the second set of boxes. If the difference between the two weight measurements is >1.0 kg, the measurement must be taken a third time following the above procedure, and recorded in the third set of boxes

In addition to the above physical measurements, an examination of the subject's ears, to determine the characteristics of his/her earwax, will be performed. The only piece of equipment required for this examination is an otoscope.

P5 Earwax Examination

<< PROCEDURE TO BE DEVELOPED >>

Circle the code corresponding to whether, based on examination, the subject's earwax was wet (code "1"), dry (code "2"), or a mixture of wet and dry (code "3"). If the subject refuses to have his/her ears examined, circle code "4" for "refused." If the examiner is unable to characterize the subject's earwax, circle code "5" for "cannot classify."

Closing Statement: Read to the subject as written. This statement informs the subject that the interview is completed, and also thanks the subject for his/her time in answering the questions in the questionnaire.

INTERVIEWER COMMENTS

The last page of the questionnaire has space to record the time the interview was completed, the length of time to complete the interview, who responded to the questions, and your opinion of the quality of the interview. Each of these items should be completed immediately after completing the interview.

1 Record the actual time that the interview ended, which would be the time when the closing statement was read to the subject. Also, circle "1" for a.m. or "2" for p.m. Right justify and zero fill as necessary. For example, if the interview ends at 2:30 in the afternoon, record the hours and minutes as |0|2|:|3|0| in the boxes provided, and circle "2" for p.m.

2 Calculate the total number of minutes the interview took to complete by subtracting the time the interview ended from the time the interview began (as recorded on the cover page of the questionnaire). Record the minutes in the boxes provided. Right justify and zero fill as necessary.

3 Circle the appropriate code for whether the interview was completed with the subject alone (code "1"), a proxy alone (code "2"), or the subject with the assistance of a proxy (code "3"). If the interview was completed with the subject alone, skip to question 5. Otherwise, continue with question 4.

4 If the interview was completed with a proxy alone or with the assistance of a proxy, circle the code corresponding to how the proxy was related to the subject. If the proxy was a relative of the subject's, other than those specifically listed, code "other relative" and specify what the proxy's relationship was to the subject on the line provided. If the proxy was not a relative of the subject's (for example, a friend), code "other nonrelative" and specify the relationship of the proxy to the subject on the line provided.

5 We are interested in your assessment of the overall quality of the interview just completed. Circle the appropriate code for whether you felt the responses to the questions were unsatisfactory (code "1"), questionable (code "2"), generally reliable (code "3"), or of high quality (code "4").
